# 30+ years of media analysis of relevance to chronic disease: a scoping review

**DOI:** 10.1186/s12889-020-8365-x

**Published:** 2020-03-20

**Authors:** Samantha Rowbotham, Thomas Astell-Burt, Tala Barakat, Penelope Hawe

**Affiliations:** 1 Menzies Centre for Health Policy, Sydney School of Public Health, University of Sydney, Sydney, Australia; 2 The Australian Prevention Partnership Centre, Sydney, Australia; 3 Population Wellbeing and Environment Research Lab (PowerLab), School of Health and Society, University of Wollongong, Wollongong, Australia; 4 School of Public Health, Peking Union Medical College and The Chinese Academy of Medical Sciences, Beijing, China; 5 National Institute of Environmental Health, Chinese Center for Disease Control and Prevention (China CDC), Beijing, China; 6 O’Brien Institute of Public Health, University of Calgary, Calgary, Canada

**Keywords:** Chronic Disease, Health Communication, Mass Media, Scoping Review

## Abstract

**Background:**

Chronic, non-communicable diseases are a significant public health priority, requiring action at individual, community and population levels, and public and political will for such action. Exposure to media, including news, entertainment, and advertising media, is likely to influence both individual behaviours, and attitudes towards preventive actions at the population level. In recent years there has been a proliferation of research exploring how chronic diseases and their risk factors are portrayed across various forms of media. This scoping review aims to map the literature in this area to identify key themes, gaps, and opportunities for future research in this area.

**Methods:**

We searched three databases (Medline, PsycINFO and Global Health) in July 2016 and identified 499 original research articles meeting inclusion criteria: original research article, published in English, focusing on media representations of chronic disease (including how issues are framed in media, impact or effect of media representations, and factors that influence media representations). We extracted key data from included articles and examined the health topics, media channels and methods of included studies, and synthesised key themes across studies.

**Results:**

Our findings show that research on media portrayals of chronic disease increased substantially between 1985 and 2016. Smoking and nutrition were the most frequent health topics, and television and print were the most common forms of media examined, although, as expected, research on online and social media channels has increased in recent years. The majority of studies focused on the amount and type of media coverage, including how issues are framed, typically using content analysis approaches. In comparison, there was much less research on the influences on and consequences of media coverage related to chronic disease, suggesting an important direction for future work.

**Conclusions:**

The results highlight key themes across media research of relevance to chronic disease. More in-depth syntheses of studies within the identified themes will allow us to draw out the key patterns and learnings across the literature.

## Background

Chronic, non-communicable diseases (hereafter ‘chronic diseases’) such as cancer, diabetes mellitus and cardiovascular disease, are a major contributor to the global burden of disease and are responsible for over 40 million deaths per year [1]. Despite increasing recognition of the urgent need to tackle chronic diseases [2] and growing evidence on both the effectiveness and cost-effectiveness of prevention [3], significant progress has not yet been made.

Chronic diseases are a complex problem, with multifactorial causes that extend beyond individual behaviours and include the social, environmental and socio-economic aspects of the environments in which people live, work and play [4]. Chronic disease prevention therefore requires coordinated, inter-sectoral efforts at the individual, community and population levels [4, 5]. For example, addressing childhood obesity is likely to require a range of interventions, including restricting junk food advertising to children, teaching cookery skills to new parents, providing nutritional information on food labels, changing school canteen menus, improving pricing and availability of fresh food, and reformulating processed foods [6]. Garnering public and political support and momentum for such actions requires a shift away from thinking at the individual level to an appreciation of the social, environmental and cultural drivers for behaviour, and an understanding of the interrelated nature of chronic disease causes, risk factors and solutions.

The public is continually exposed to mass media, including news, entertainment and advertising media, through channels such as television, radio, movies, newspapers, magazines and the internet. Such exposure is likely to play a key role in shaping attitudes and behaviours of relevance to chronic disease prevention [7]. News media lies at the nexus of the public and policy agenda and news coverage of issues and events both shapes and reflects public and political opinion [8]. While print newspapers are considered to be something of a ‘dying industry’, online news media exposure continues to increase, with much of the population having direct access 24 hours a day, 7 days a week, from almost any location [9]. Thus, the news media continues to be a vital social institution and digital technologies have reshaped this industry in recent years. In particular, the emergence of an array of new actors, such as BuzzFeed, The Huffington Post and The Conversation, along with the growth of social media platforms and blogs, has resulted in significant changes in who and what constitutes the news media institution. Further, the ease of sharing content across social networks, as well as the so-called ‘echo-chamber’ effect, have changed the flow of information, including what gets amplified and how. Understanding how these shifts in the media landscape affect the public and political agenda setting process will therefore be of increasing importance going forward.

The study of news media communication occurs within a multidisciplinary paradigm with roots in sociology and political science, and draws heavily on framing theory, which concerns the “holistic study of media effects on individuals and audiences” (p. 423 )[10], focusing on four elements of the communication process: the sender, the receiver, the (informative) message and culture [10]. Framing theory posits that messages are packaged in particular ways to emphasize certain pieces of information and de-emphasise others [11, 12], and particular framings will “promote a particular problem definition, causal interpretation, moral evaluation, and/or treatment recommendation” (p.53 )[11]. Research within this paradigm has revealed that the nature of information conveyed through the media, including what gets reported, the amount of coverage received and the way in which it is represented can have a powerful effect on knowledge, attitudes, and behaviours [12–18]. In addition to shaping societal attitudes towards issues, media coverage is a societal product in itself, such that issue framing is constrained by social structures, values and norms [19, 20]. Thus understanding how issues are framed can provide insights into wider trends in society.

Other forms of media, including entertainment, commercial advertising, and social marketing are also likely to play a role in influencing public attitudes, opinions and behaviours of relevance to chronic disease. For example, commercial advertising through television commercials, online advertising campaigns, and point of sale advertising are used often to influence consumer behaviours that may increase the risk of chronic diseases, such as encouraging consumption of unhealthy foods or alcohol (e.g., [21–23]), and may also encourage the purchase of products or services that promote health, such as commercial weight loss programs or meal plans. Social marketing campaigns may employ mass media channels to encourage healthy behaviours, such as smoking cessation, responsible alcohol consumption, and cancer screening (see, for example [24] for a review of mass media campaigns to change health behaviour). Entertainment media, such as films, television shows and music videos may influence attitudes and behaviours of relevance to chronic disease for example by using plotlines that raise awareness of issues related to chronic disease, or model behaviours such as smoking and alcohol consumption [25].

In recent years there has been a proliferation of media research on issues of relevance to chronic disease (including disease risks, causes and solutions). While such a growth in research is promising both in terms of interest in this field and the potential for new and useful knowledge to emerge, the volume and breadth of evidence can be overwhelming for those who need to access the key messages from this research, such as policy makers and practitioners. In particular, both original research articles and reviews have tended to ‘zoom in’ on specific issues, such as how obesity is portrayed within news media (e.g. [26–29]) or the framing of arguments around smoking restrictions (e.g. [30–35]), and to date, no comprehensive synthesis or mapping of the area as a whole exists.

Within this paper we aim to provide an initial mapping of media research on topics of relevance to chronic disease. In particular, we explore the scope and nature of research on how issues related to chronic disease prevention have been portrayed across various forms of media in order to provide an overview of the key focus areas and highlight gaps and opportunities for future investigation. In doing so we seek to address the following research questions:

- What are the key trends in research on media coverage of chronic diseases?
- How has research on media coverage of chronic diseases changed over time?
- What are the key gaps and opportunities for further research on media coverage of chronic diseases?

## Methods

### Aim

To map existing research examining mass media content of relevance to chronic disease.

### Design

A scoping review was selected as it allows for rapid mapping of the key concepts underpinning a research area and the main sources and types of evidence available [36] and is most appropriate when endeavouring to: examine the extent, range, and nature of research activity; summarize and disseminate research findings; and/or identify gaps in the existing research [37]. The methodology for this scoping review was based on previous the framework outlined by Arksey and O’Malley [37] and ensuing recommendations made by Levac, Colquhoun and O’Brien [38]. For the purpose of this study, a scoping review is defined as a type of research synthesis that aims to “map the literature on a particular topic or research area and provide an opportunity to identify key concepts; gaps in the research; and types and sources of evidence to inform practice, policymaking, and research” (p.2 )[39]. The review included the following five key phases: (1) identifying the research question, (2) identifying relevant studies, (3) study selection, (4) data extraction, and (5) collating, summarizing, and reporting the results. The review was completed in accordance with the PRISMA-ScR checklist [40] and copy of the completed checklist can be found in Additional file 2.

### Search strategy

We searched three electronic databases: MEDLINE (1946–), PsycINFO (1967–), and Global Health (1973–) via OVID in July 2016, to identify studies published in English. As the purpose of this review was to provide an overview of media research of relevance to lifestyle-related chronic diseases (e.g. cardiovascular disease, cancer, and diabetes), and their risk factors (e.g. smoking, alcohol consumption, obesity, physical activity), search terms were constructed across three concepts: topics and issues related to chronic disease (including search terms related to chronic diseases, risk factors, and public health), types of media (including advertising, news, entertainment and social media), and content or framing (see Table 1). Search terms were piloted and refined prior to use, including consultation with experts and checking for capture of studies that the authors expected to be included.

**Table 1:** Search strategy

Search terms	(Public Health/ OR Health promotion/ OR Health Education/ OR Health Policy/ OR Overweight/ OR Obesity/ OR Alcohol Drinking/ OR Binge Drinking/ OR Exercise/ OR Diet/ OR Food habits/ OR Smoking/ OR Smoking cessation/ OR Risk Factors/ OR Diabetes Mellitus, Type 2/ OR Hypertension/ OR Cardiovascular Diseases/ OR Chronic Disease/ OR Cancer) AND (Mass Media/ OR Communications Media/ OR Social Media/ OR television.mp OR radio.mp OR news*.mp OR media.mp OR Marketing/ or Marketing of health services/ or Social marketing/ OR advertis*.mp) AND (framing.mp OR frame.mp OR content analysis.mp)
Search limits	English language

*Note:* / denotes a MeSH heading; .mp denotes a free text search term

**Table 2:** Definitions of media categories within this review

Media type	Definition
News media	Refers to any media that provides news or information, including print media (newspapers, magazines), broadcast news (TV and radio news) and online news sites
Entertainment media	Refers to non-news forms of entertainment, such as music, film, and television shows
Social media	Refers to websites and applications that allow users to create and share content or participate in social networking (e.g. Facebook, Twitter, blogs)
Marketing media	Refers to media channels through which promotional messages are communicated to the public, including both commercial and social marketing

### Study selection

In line with the recommendations of Levac, Colquhoun and O’Brien [38] the criteria for study inclusion were refined through discussion amongst the research team in an iterative manner as the reviewers became more familiar with the research. Studies were included if they reported original research related to media representations of chronic disease, including how issues are framed, the impact or effects of media representations (e.g. on public opinion or behaviour), and factors that influence media representations. Chronic diseases were defined as non-communicable conditions, for which there are a range of lifestyle-related risk factors, and included cardiovascular disease, cancer, and diabetes. Studies were included if they focused on any issues related to chronic disease, including prevalence, causes and risk factors (e.g. obesity, high blood pressure, diet, physical inactivity, alcohol, smoking, social/economic inequality), and/or prevention (including policies and programs). Although mental health issues were not a key focus of our search, a number of articles related to mental health were captured within our search terms. These were included these as they represent an important group of chronic conditions for which media coverage is likely to impact on public and political attitudes towards prevention and treatment. Only original research articles were included; other types of publications, including systematic reviews, meta-analyses, letters, and guidelines were not included within this review. Due to the volume of results returned by the database searches, further searching of grey literature and hand searching of reference lists and journals was beyond the scope of the study.

We included published articles that focused on any form of public media, including news media (e.g. newspapers, magazines, TV news), social media (e.g. Twitter, Facebook, blogs), entertainment media (e.g. TV sitcoms, movies, music videos), and/or advertising and marketing (including commercial advertisements and social marketing) (see Table 2 for definitions of media types). Conference abstracts, dissertations and other unpublished materials were not included within the review.

**Fig. 1:**
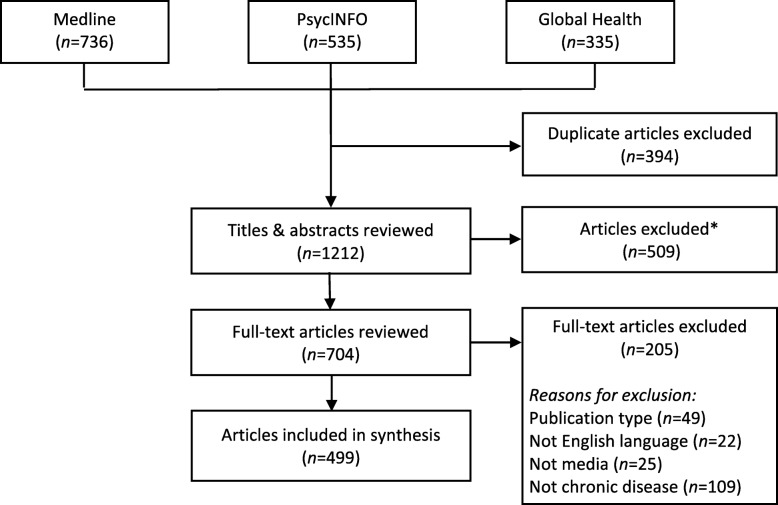
Flow diagram depicting search strategy and review process. * Articles excluded at title and abstract screening stage did not meet inclusion criteria, such as studies of media framing of communicable diseases, studies that did not examine media content or framing, review and theoretical articles, and articles not published in English.

One reviewer (SR) screened article titles and abstracts for eligibility and reviewed the full-text of articles identified as ‘eligible’ or ‘unclear’. For reliability purposes, a second reviewer (TAB) reviewed a random subset of articles on the basis of titles and abstracts (*n*=100) and full-texts (*n*=30). There was a good level of agreement at both stages (title and abstract: 86% agreement; Cohen’s *k*= .71; full-text: 93% agreement; Cohen’s *k*=.84) and all disagreements were discussed and resolved. Figure 1 outlines the flow of articles through the review process.

### Data extraction

A data extraction template was developed in Microsoft Excel to extract key details about included studies. Extracted data included study characteristics, research focus, sample and methods, media types and topics covered. The data extraction form was initially reviewed by the research team and pretested by SR and TAB before use, and was continually refined during the early stages of data extraction. The characteristics of each full-text article were extracted by one reviewer (SR or TB), while a second reviewer (TAB or SR) performed data extraction on a randomly selected subset of full-text articles to check for consistency in information extracted. Comparison of extracted data indicated a high level of consistency and all disagreements were discussed and resolved.

### Data synthesis

Extracted data were imported into NVivo qualitative data analysis software [41] for additional coding and data synthesis. Following the process outlined by Arksey and O'Malley [37], this began with a quantitative, descriptive analysis of the studies included within the review, including the distribution of studies over time, and media type and health topic in order to identify the dominant areas of research and any significant gaps. Following this a thematic approach [42] was employed, in which data were coded inductively to identify key themes in the focus areas and research questions of the included studies, attending to similarities and differences within and across the main media types in a way which accounted for the heterogeneity across studies. Data synthesis was performed by one reviewer (SR) and refined through ongoing discussion with the research team. Due to the volume of studies identified, a comprehensive synthesis of findings across all studies was beyond the scope of the current paper. Instead we have sought to categorise studies according to common themes and present examples of studies and key findings to highlight these.

## Results

### Study characteristics

Four hundred and ninety-nine studies were included in the review. Table 3 provides a description of the included studies and details of the key characteristics of each included study are also provided (see Additional File 1). The majority of studies (*n*=297; 60%) were conducted in the USA, followed by Australia (*n*=52; 10%), Canada (*n*=37; 7%), and the United Kingdom (*n*=31; 6%), and only 13 (3%) studies took a multi-country approach (e.g. a comparative analysis of media coverage across countries). News and information media were the most frequent focus of studies followed by marketing media.

**Table 3:** Description of included studies

Variable	*n*^a^	(%)
Continent		
Africa	4	(0.8)
Asia^b^	31	(6.2)
Australasia	62	(12.4)
Europe	72	(14.3)
North America	331	(66.3)
Central or South America	5	(1.0)
Publication year		
1985-1989	1	(0.2)
1990-1994	9	(1.8)
1995-1999	20	(4.0)
2000-2004	43	(8.6)
2005-2009	125	(25.0)
2010-2014	207	(41.4)
2015-2016	94	(18.7)
Media categories^c^		
News	264	(52.9)
Entertainment	45	(9.2)
Social media	49	(9.8)
Marketing	159	(31.8)
Methods		
Descriptive	446	(89.2)
Experimental	60	(12.0)
Interview, survey or focus group	46	(9.2)
Media sample timeframe		
Less than 1 year	151	(30.1)
1 – 5 years	164	(32.7)
6 – 10 years	55	(11.0)
11 – 15 years	27	(5.4)
More than 15 years	35	(7.0)
Not specified	67	(13.4)
Media channels		
Television	148	(29.7)
Newspapers	179	(35.7)
Magazines	87	(17.3)
Radio	9	(1.8)
Movies	12	(2.4)
Music	6	(1.2)
Online (incl. online news, web pages and social media)	93	(18.6)
Other (incl. billboards, product packaging, constructed messages)	53	(10.6)
Total Articles	**499**	

^a^ Due to some articles being coded more than once within a category, the total within each category may exceed 499.

^b^ Includes 1 study from Turkey

^c^ See Table 2 for definitions of each of the media categories

Studies were categorised according to the approach taken. Descriptive studies were those that involved an analysis (whether qualitative, quantitative or both) of media content, and were the most common study type within the sample (*n*=446). Descriptive studies were most often cross-sectional in nature, i.e., the analysis of news coverage of a particular issue at a particular point in time, although some studies took a longitudinal approach, for example examining patterns in media coverage over time. A smaller number of studies (*n*=60) employed an experimental approach, seeking to test the impact of differences in how chronic diseases were portrayed on a specified variable, e.g. testing the effect of presenting different framings of a news story on public attitudes to chronic disease, and included both lab-based and naturalistic studies.

For studies of media content, the sample timeframe was most often between 0 and 5 years in duration, with a small proportion sampling over a duration exceeding 10 years. Newspapers were the most common media channel examined within our sample, followed by television and online media.

**Fig. 2:**
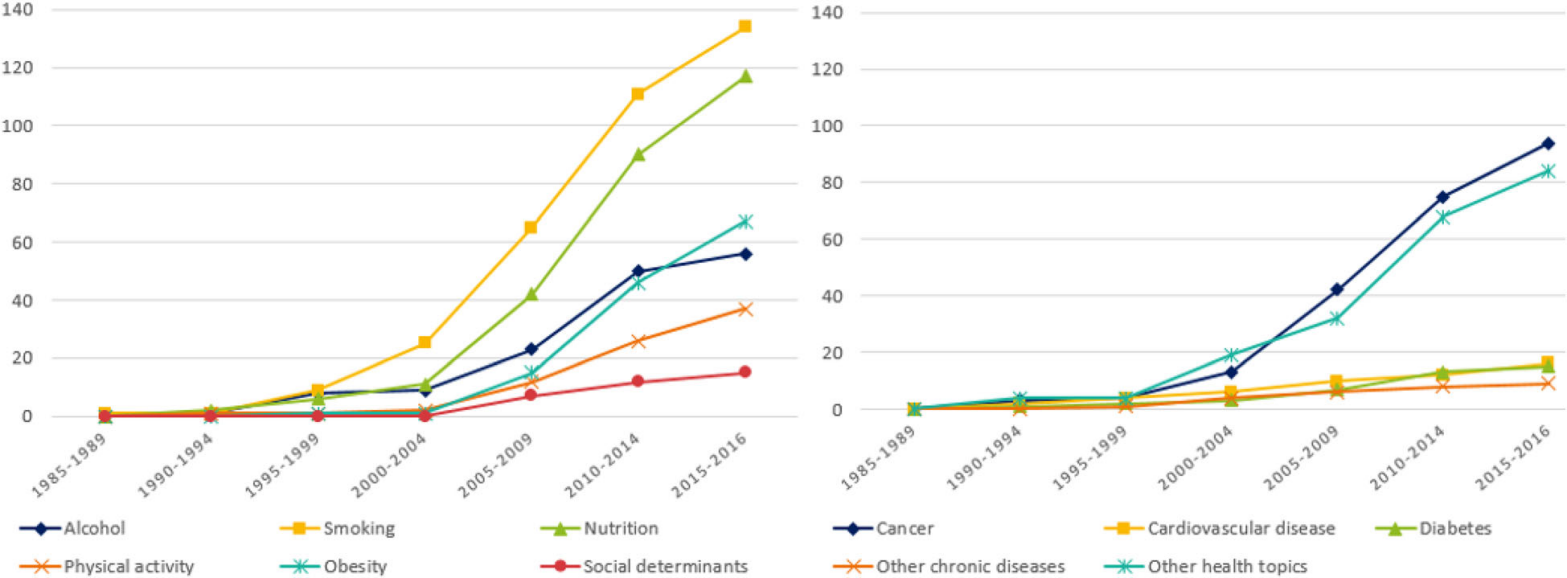
Cumulative frequency of studies over time, by health topic. *Note. The category ‘other health topics’ includes oral health, mental health, child and maternal health.*

The number of studies increased over time. Studies covered a range of health topics related to chronic disease prevention, with the majority of studies (*n*=342; 69%) focusing on behavioural risk factors related to chronic disease, particularly smoking and nutrition. Just over a quarter of studies (*n*=134; 27%) focused on specific chronic diseases, including cancer (*n*=93; 19%), type 2 diabetes (*n*=15; 3%), cardiovascular disease (*n*=16; 3%), and other chronic diseases (e.g. chronic kidney disease, hypertension; *n*=9; 2%). Eighty-three studies (17%) focused on other health topics relevant to chronic disease prevention, such as oral health, mental health, and child and maternal health. The cumulative frequency of studies for each health topic over time is displayed in Fig. 2.

### Synthesis of included studies

**Fig. 3:**
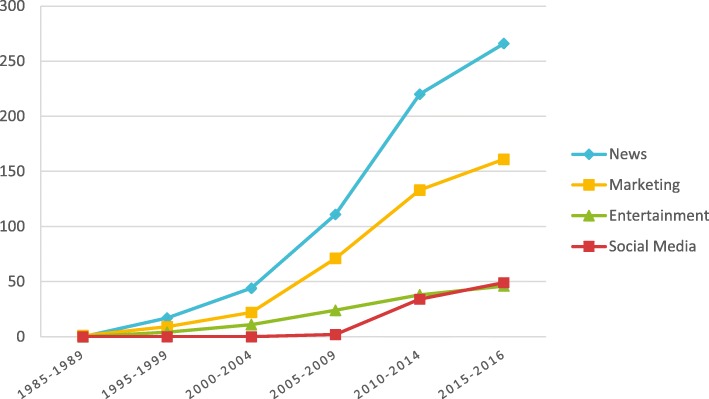
Cumulative frequency of studies over time, by media type. *Note. See Table**1**for definitions of media types used*

Due to the volume of studies in our sample, for the purpose of synthesis we have grouped studies according to four broad media categories: 1) news media, 2) entertainment media, 3) social media, and 4) marketing media (see Table 2 for definitions of the media types used within this study). Mapping of the cumulative frequency of studies over time (see Fig. 3) revealed that news media has remained the most frequent focus of studies, followed by studies of marketing media (including both commercial marketing, e.g. of unhealthy products such as cigarettes, and social marketing, e.g. smoking cessation campaigns). However, in recent years, there has been an increase in the number of studies examining entertainment media such as television dramas, music and film, as well as an increase in studies of social media, such as Facebook and Twitter.

**Fig. 4:**
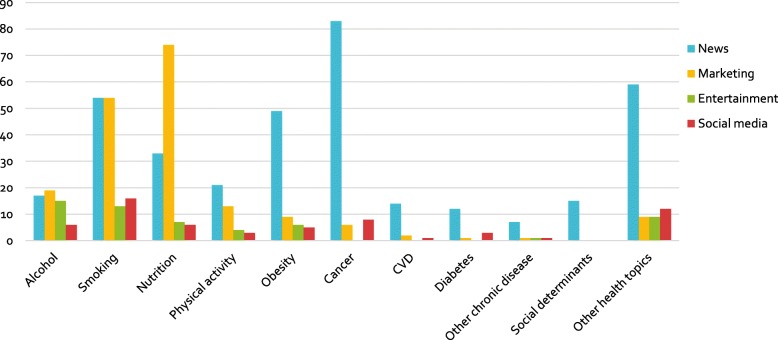
Number of studies per topic by media category. *Note. The category of ‘other health topics’ includes oral health, mental health, child and maternal health, and general health topics.*

The distribution of health topics varied across the categories of media examined (see Fig. 4). While chronic diseases, obesity and other health topics were most frequently examined in the context of news media, nutrition was considered most often in relation to marketing media, and smoking, alcohol, and physical activity were considered at a similar rate in both news and marketing media.

### News media

**Fig. 5:**
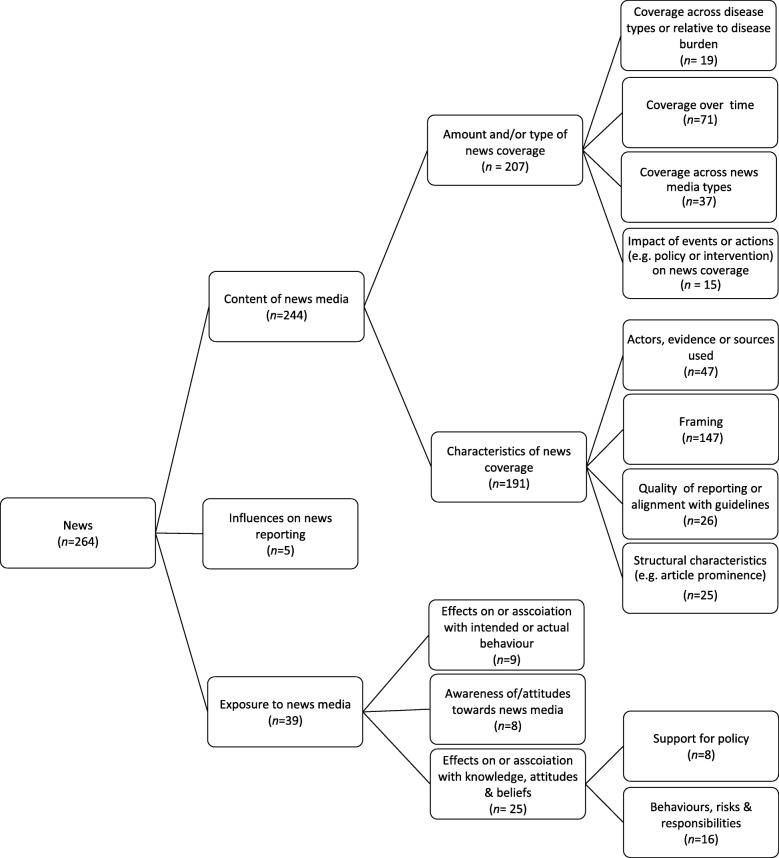
Key themes across news media articles, including number of articles per theme. *Note. Theme groupings are not mutually exclusive and articles are often coded to multiple themes.*

A total of 264 studies reported research on news media. Studies of news media included descriptive analyses of news content, studies of audience exposure to news, and investigation of factors that influence news reporting. Figure 5 provides an overview of the main themes and sub-themes of research within the news media category, and these are summarised in more detail below, along with example studies to illustrate.

#### Content of news media

A large proportion of studies (*n*=244) focused on the *content of news media*, particularly in terms of *the amount and/or type of news coverage* of health issues (*n*=207), and the *characteristics* of such coverage (*n*=191). The majority of studies used content analysis approaches (e.g. [43–46]), with a smaller proportion of studies using other qualitative approaches, such as discourse analysis, to explore the patterns and trends in news media coverage (e.g. [47, 48]).

Of those studies examining the *amount and/or type of news coverage,* a key focus was on *news coverage over time* (*n*=71)*,* particularly in terms of changes in the amount of coverage received and key themes within the coverage (e.g. [26, 28, 29, 32, 48–67])*.* For example, studies have found that the amount of news coverage of obesity [28, 29], cancer [58, 59], and smoking-related harms [32, 60] have increased over time. Other studies examined how the nature of news coverage had changed over time, for example demonstrating temporal changes in predominant themes and framing of tobacco [61, 62], alcohol use [53, 63, 64], obesity [28, 29, 65, 66], social and racial disparities in health [68], and mental health issues [67]. Other studies have used critical analysis methods to track how issues such as second hand-smoke have emerged over time [48].

Another focus area was *the impact of events or actions (e.g. implementation of interventions and policies) on news coverage* (*n*=15; e.g. [34, 69–75]). For example, one study considered how the framing of obesity shifted over the course of a sugar-sweetened beverage reduction media campaign [75], while another considered how news coverage of skin cancer changed following the release of a key public health report on cancer [70].

Nineteen studies compared the *amount of news coverage received by different health topics* (e.g. [68, 76–86]) and/or whether the *amount of news coverage received was proportionate to the burden of the problem* (e.g. [79, 87–92]). For example, two studies demonstrated that news coverage of a range of cancers is underrepresented relative to their population burden [89, 92]. Finally, studies have also considered how *coverage differs across news media*, including differences across news media aimed at different cultural or language groups (e.g. [93–98]), geographical regions (e.g. [99]), and news media types, such as middle market versus quality newspapers (e.g. [81]).

Studies focusing on *characteristics of news coverage* predominantly considered the *framing* of issues related to chronic disease prevention (*n*=147). The synthesis revealed that the most frequent focus was on valence of coverage (i.e. whether issues were framed positively or negatively) (e.g. [94, 99–109]), and responsibility for causes and solutions (e.g. individual versus government or industry responsibility) (e.g. [31, 61, 110–119]), with studies focusing on obesity being particularly prevalent here (e.g. [26, 27, 66, 75, 120–123]). Studies of valence and framing included those examining news coverage of particular behaviours of relevance to chronic disease, such as breastfeeding [94] and smoking [100], as well as those examining support for policy actions, such as regulation to limit sales of sugar sweetened beverages [102], an ‘alcopop tax’ on ready-to-drink spirits in Australia [103], and legislation for plain packaging of tobacco [124]. Examples of other specific types of frames studied included gain versus loss frames (e.g. [125, 126]), thematic (which focus on the broader context) versus episodic frames (which focus on the immediate event or incident and give little or no context) (e.g. [29, 75, 82, 127]) and health versus appearance frames (e.g. [128]).

Twenty-six studies considered the *quality of news media content*, including how well content aligned with guidelines or recommendations (e.g. [94, 129–136]). For example, one study examined the accuracy of information and level of stigmatisation around obesity in newspaper articles [61], while another [137] considered the relationship between the amount of news coverage of food groups compared with the recommended amount of consumption of these foods. Finally, a number of studies also considered *structural characteristics* of news media, including the prominence of articles and use of images (e.g. [71, 112, 138, 139]), while others considered the *actors, evidence or sources used* within news articles (e.g. [80, 119, 140–144]).

#### Factors that influence news reporting

Five studies examined *factors that influence health reporting* in the news media. Two used surveys to examine associations between journalist characteristics such as gender, age, ethnicity and experience, and news story characteristics, such as framing, source utilisation, and news priorities [145, 146]. A third study explored how journalists judge the newsworthiness of stories that report race-specific health disparities and whether informing journalists of audience reactions to different kinds of framing influences these judgements [147]. The remaining two used interviews to explore the barriers faced by journalists when covering health disparities in the media [148], and to seek the opinions of health experts on the problems of dominant obesity-prevention frames (personal responsibility and the environment) and explore alternative frames [149].

#### Exposure to news media

Of the 39 studies examining audience *exposure to news media,* eight focused on *awareness of and/or attitudes towards news media,* including investigations of public awareness of news coverage of chronic disease topics and health promotion campaigns (e.g. [150–152]), attitudes towards news coverage of issues such as obesity [153], factors that drive audience interest in prevention [154], and sociodemographic influences on exposure to news media [155].

A number of studies considered *the effects of exposure to news media on or association with actual or intended behaviours* (*n*=9; e.g. [136, 156–162]), or on *knowledge, attitudes, and beliefs* about the causes, consequences and solutions to a range of health issues (*n*=25, e.g. [49, 88, 120, 147, 159, 160, 163–174]). Such studies often employed experimental designs to test the impact of differences in framing (e.g. negative versus positive, thematic versus episodic, and gain versus loss frames), evidence use, and message salience (e.g. [159, 160, 168, 170, 171, 175]). For example, one study found that participants who read a news article in which obesity was framed in societal (i.e. highlighting the role of the environment), rather than individual terms, were more likely to attribute obesity to social conditions and identify the government, food industry, and marketing sector to be responsible for solving the problem [160]. Other studies examined the relationship between community level news exposure and individual attitudes and behaviours using a combination of content analysis, surveys, interviews, and community-level health data (e.g. [88, 152, 161, 162]). For example using content analysis of local news media coverage of tobacco and community survey data, Smith and colleagues [162] found an association between volume of tobacco related newspaper articles and perceived harms of smoking, perceived peer smoking, disapproval of smoking, and smoking within the past 30 days.

Eight studies considered the *impact of news exposure on attitudes towards public policies* to tackle chronic disease [120, 168–170, 172, 173, 176, 177]. For example, one study found that thematic framing (i.e. incorporating information on context, risk factors, prevention strategies, and social attributions of responsibility), increases support for policy change across a range of health issues, including obesity, smoking and diabetes [168], while another found that a taste-engineering frame (i.e. highlighting strategies used by the food industry to increase consumption), increases support for food and beverage policies [172]. In contrast, individualising the problem of obesity by identifying an individual child within a news story was associated with reduced support for obesity policies, regardless of how causes of obesity were framed [120]. Finally, a study in the US demonstrated that the effect of framing on policy support is mediated by political opinion, with Democrats expressing a higher level of support for a range of public health policies after exposure to a social determinants of health frame, while Republicans expressed a lower level of support following exposure to the same message [170].

### Entertainment media

**Fig. 6:**
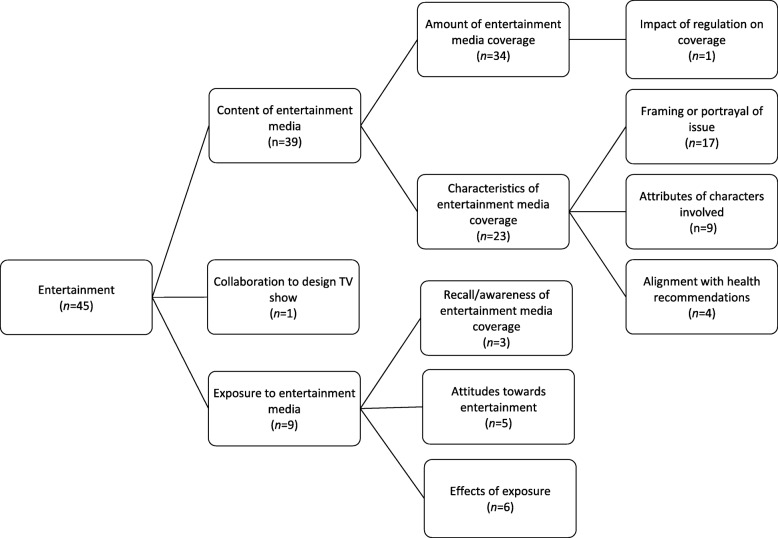
Key themes across entertainment media articles, including number of articles per theme. *Note. Theme groupings are not mutually exclusive and articles are often coded to multiple themes.*

Forty-five studies examined entertainment media, with most focusing on televised entertainment (including reality shows, drama, soaps and documentaries). The majority of studies involved descriptive analyses of entertainment media, and/or investigations into the effects of exposure to entertainment media. Figure 6 provides an overview of the main themes within this media category.

#### Content of entertainment media

Thirty-four studies considered the *amount of coverage* received by health topics (e.g. chronic disease prevention [178, 179];), products (e.g. alcohol, cigarettes, unhealthy food [180–182];) and behaviours (e.g. eating, drinking, smoking, weight stigmatization [183–191];) within entertainment media. One study considered the impact of regulation on the frequency of tobacco placement in movies [192].

Over half of the studies a considered the *characteristics of coverage* in entertainment media (*n*=23), for example whether behaviour is portrayed in positive or negative terms (e.g. [180, 188, 193]), or using message appeal strategies such as sexualisation, glamour or humour (e.g. [182, 185, 194]). For example, one study found that depictions of alcohol in popular music were associated with wealth, sex, and luxury [194]. Four studies considered whether portrayals of food and drinks within entertainment media *aligned with health recommendations*, finding that they often do not [187, 195–197]. Nine studies examined the *attributes of the characters involved* in entertainment media representations, for example in terms of gender, ethnicity, and age (e.g. [186, 198]).

#### Exposure to entertainment media

Of the nine studies that considered exposure to entertainment media, the main focus areas were *audience awareness* of the issues portrayed through entertainment media (*n*=3; e.g. [152, 193, 199]), *audience attitudes* towards portrayal of these issues (*n*=5; e.g. [152, 193, 200, 201]), and the *effects of exposure to entertainment media on attitudes and behaviours* (*n*=6; e.g. [201–203]) For example, one study explored audience awareness of and attitudes towards an online social marketing campaign coupled with a popular TV series which aimed to reduce harmful alcohol consumption [193], while another examined the impact of alcohol portrayals in a television soap on adolescents' attitudes towards alcohol [202].

### Social media

**Fig. 7:**
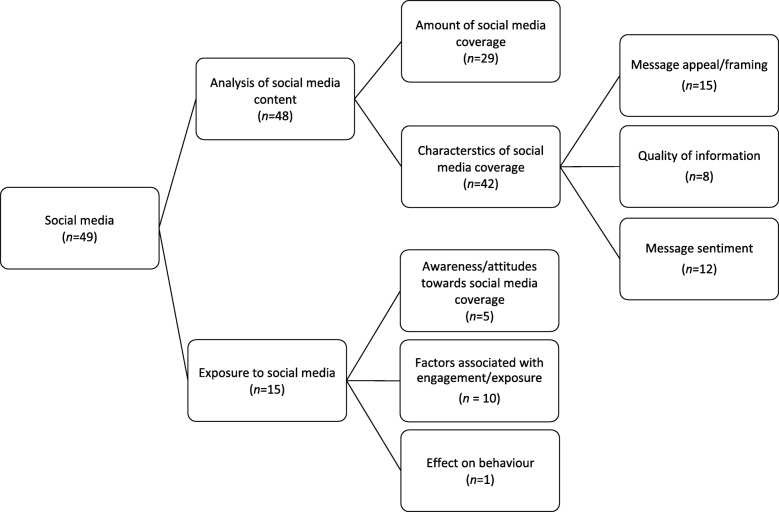
Key themes across social media articles, including number of articles per theme. *Note. Theme groupings are not mutually exclusive and articles are often coded to multiple themes.*

Forty-nine studies examined social media channels including Twitter and YouTube, social networking sites such as Facebook and MySpace, blogs, and online discussion boards. Studies of social media primarily examined the *content of social media* (*n*=48) and/or *factors related to social media exposure* (*n*=14), including levels of social media engagement and the effects of exposure to messages via social media. Figure 7 provides an overview of the main themes of research within this media category.

#### Analysis of social media content

Of the 48 studies that examined the *content* of social media messages, 28 focused on the *amount of coverage of issues* related to chronic disease, and included studies of the number of tweets, blog posts or online comments about a particular issue or topic (e.g. smoking regulation, e-cigarettes, or alcohol use) (e.g. [204–211]). For example, one study examined the number of tweets related to hookah smoking [212], while another examined the frequency of health-related tweets by health professionals on Twitter [211].

Thirty-five studies examined *characteristics of social media content*. These included considerations of how issues such as smoking, alcohol use, cancer and eating disorders are depicted, for example in terms of the key themes in coverage of health topics (e.g. [207, 212–215]), the use of message appeal strategies and images (e.g. [216–220]), and studies of the quality of information conveyed through social media, including whether the information aligned with health recommendations (e.g. [210, 221, 222]). For example one study examined how responsibility and solutions for obesity are framed within YouTube videos [215]. Other studies considered how users talk about issues on social media (e.g. [223–226]), including the valence of messages, including public sentiment towards policy and regulation (e.g. [124, 227, 228]) and health promotion campaigns (e.g. [193, 229]).

#### Exposure to social media

There were three main sub-themes identified within studies of exposure to social media coverage. The first examined audience *awareness of or attitudes towards social media coverage* of issues related to chronic disease (e.g. [221, 229–232]). For example, one study used focus groups and surveys to explore women’s attitudes towards healthy eating blogs and their beliefs and attitudes towards using such blogs to improve their dietary habits [230], while another examined how friends react to adolescents’ portrayals of alcohol on Facebook [232]. The second sub-theme contained studies that examined the *factors associated with exposure to and/or engagement with social media coverage of issues related to chronic disease* (e.g. [207, 232–234]). These included a study of the demographic factors associated with display of alcohol references on MySpace [207], and another examining whether exposure to tobacco content online was associated with smoking status [234]. Finally, one study examined the *effect of exposure to social media messages on behaviour* [235].

### Marketing media

**Fig. 8:**
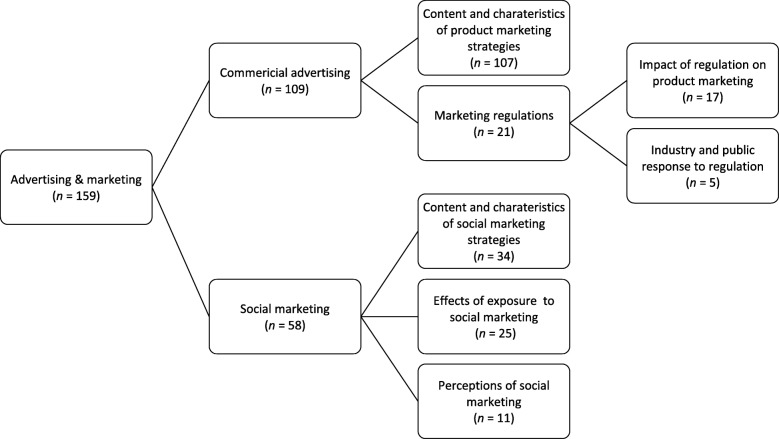
Key themes across commercial advertising and social marketing media articles, including number of articles per theme. *Note. Theme groupings are not mutually exclusive and articles are often coded to multiple themes.*

Overall, 159 studies focused on marketing media, of which the majority concerned commercial marketing (*n*=110), with a smaller proportion concerning social marketing (e.g. health promotion campaigns) (*n*=58). Figure 8 provides an overview of the main themes within this media category.

#### Commercial marketing

Of the 109 studies focused on commercial marketing media, the majority (*n*=107) focused on examining product portrayals within commercial advertisements and product packaging, including frequency of advertisements and *content and characteristics of marketing strategies* (e.g. [236–268]), with the majority of studies focusing on tobacco and food advertising. For example, one study explored cigarette marketing strategies in India by examining cigarette advertising on billboards, storefronts and at point of sale as well as in films, magazines and newspapers [238], while another examined how tobacco companies increase magazine advertising in January and February to pre-empt quitting by providing cues to smoking [239]. Other studies examined how marketing strategies such as physical activity references (e.g. [241, 242, 269]), personal attributes (e.g. [270]), emotional appeals (e.g. [252]), and sexual imagery (e.g. [271]) were used to market products.

Nearly a quarter of studies (*n*=21) focused on marketing regulations, with the majority of these considering the impact of regulation on advertising practices (e.g. [255, 271–278]). For example, one study evaluated the impact of industry self-regulation on television marketing of food to children [276], while another examined adherence to federal and voluntary standards for alcohol advertising in magazines [271]. Both studies found that while advertising regulations resulted in fewer advertisements, industries find ways to circumnavigate such restrictions [271, 276]. Other studies examined industry counter-strategies in response to advertising regulation, such as the use of brand imagery to promote tobacco use in the face of advertising restrictions (e.g. [277, 278]).

#### Social marketing

Fifty-eight studies explored social marketing for health promotion. Of these, 34 involved an analysis of the content and characteristics of social marketing media, such as content analysis of the characteristics of antismoking or physical advertisements [269, 279]. Other studies explored the impact of social marketing strategies on consumer’s attitudes and behaviours, for example using experimental approaches to examine the impact of message framing (e.g. gain- versus loss-framing) on health-related attitudes and behaviours such as seeking smoking cessation support [280], visiting the dentist [281], healthy snack choice [282], and chronic disease risk perception [283]. Eleven studies used focus groups, interviews and/or surveys to explore public perceptions of social marketing strategies (e.g., awareness, recall, liking, and perceived effectiveness of health promotion campaigns) [76, 284–286].

## Discussion

We aimed to explore the scope and nature of research on media coverage of issues related to chronic disease. Research in this area has proliferated over the last three decades, with a particularly steep increase in the number of studies published since 2000. Across the sample, behavioural risk factors for chronic disease, tobacco smoking and nutrition especially, have received the most research attention. The volume of research on media portrayals of nutrition appears to be driven by research on advertising media, where there has been considerable focus on how unhealthy foods are marketed, particularly to children. In contrast, the volume of articles related to smoking seems to be driven by a combination of studies of cigarette marketing and news media representations of smoking. The large proportion of research articles examining media portrayals of smoking is unsurprising when considered in light of the huge shifts in public and political opinion in relation to tobacco control legislation, policy, and program support in recent decades. For example, since the 1970s in Australia, tobacco control advocacy, which is often enacted through news and other media coverage, has resulted in significant gains including advertising bans, increased taxation and banning of smoking in indoor spaces [287]. Much of the pioneering work in media advocacy and framing of public health issues therefore originated in tobacco control, and has paved the way for research into media portrayals of other public health issues [255, 288].

The findings revealed a tendency for studies to focus on single health topics, with those studies that did consider multiple health topics tending to either examine closely related topics, such as nutrition and obesity, or focus on the amount of coverage across different topics [91, 183]. Comparative analyses, such as those considering similarities and differences in media coverage of policies to encourage different health behaviours, such as smoking cessation and weight control [289] or considering the differential effects of framing effects on audience attitudes depending on health topic [168] were few and far between. In addition, there was only a handful of multi-country studies, for example, exploring how obesity was framed within news media in France and the US [290], and the impact of policies around online marketing of food to children across three countries [291]. Comparative approaches across countries and settings allow for exploration of the various contextual and cultural factors that influence media portrayal of issues related to chronic disease prevention, and allow broader insights and generalisations to be drawn. While such approaches may be challenging to undertake (not least when there are language differences to take into account), cross-country policy approaches to chronic disease prevention, such as those within the European Union or driven by the World Health Organisation require cross-country understanding of the media landscape.

The majority of studies in this review have focused on analyses of the *content* of media, with a large proportion of studies in each media category considering the *content and characteristics of media coverage* of a variety of issues (news = 92%, entertainment = 85%, social media = 98%, marketing = 88%). In contrast, a much smaller proportion of studies in each media category were concerned with the impact of *exposure to media* (news = 15%, entertainment = 20%, social media = 31%, marketing = 16%). This difference may reflect the relative ease of describing and analysing media content compared with assessing the impact of exposure to content on factors such as audience attitudes and behaviours. However, while studies of media content are valuable in demonstrating what issues are likely to gain traction within the media and provide important insights into the way that issues are being communicated to the public, it is also critical to understand the impact that such communications have on audiences’ attitudes and behaviours. The effects of message framing on audiences cannot be taken for granted as audiences are not passive receptacles for information. Instead individuals actively engage with messages to a greater or lesser extent, and may accept, reject or negotiate how they interpret information, particularly in light of their existing knowledge, attitudes, beliefs, biases and previous experiences [292]. Understanding the factors that influence message interpretation is crucial in thinking about audience segmentation and targeting, and the range of potential impacts that a single message could have on different groups and across contexts of contrasting social and physical geographies. A good example of this is a study of differences in Republican vs. Democrat voter attitudes towards policy following presentation of the same message [170]. However, studying the effects of exposure to media is challenging, particularly as the social nature of interpreting media messages is difficult to capture through experimental methods, and reactions studied under artificial settings may not provide insights that are generalisable to community-based settings [293, 294]. However, social media platforms may provide us with a natural laboratory in which these kinds of effects could be studied (see below for a discussion of this).

There were also very few studies that consider the factors that influence media reporting of issues related to chronic disease. News reporting can be shaped by personal and professional biases [295], and understanding these biases is vital if we are to move beyond simple description of news stories towards strategies to change the way that issues related to chronic disease are portrayed.

In terms of the types of media that have been studied, news and marketing media have been the most frequent focus of research across the time period, with comparatively fewer studies of social media, such as Twitter, Facebook and YouTube. This is likely to be a historical bias which reflects the relatively recent growth of social media and advances in techniques for the analysis of social media data. In recent years the media landscape has changed, and continues to change rapidly, as people increasingly use social media platforms to access news and entertainment media, as well as to interact with others [9]. An understanding of how issues related to chronic disease are being portrayed and discussed within these social media spaces will be crucial going forwards. In particular, social media platforms represent a more interactive form of media engagement that traditional channels such as newspapers and radio, allowing audiences to share and discuss information in real time, while algorithms such as those used by Facebook use a range of information to target the content that users are exposed to. Social media platforms therefore provide fertile ground for research examining the diffusion and reverberation of information within and across networks, audience discussion and opinions about a range of issues, and provide opportunities for experiments to test how audience react to and interact with different kinds of messages related to chronic disease. There is already pioneering work happening within this space, and we would expect to see a rapid growth in research in these areas in the coming years.

### Limitations

Within this scoping review we have provided a snapshot of the current landscape of research on media portrayals of issues related to chronic disease, highlighting the key focus areas across the field as a whole, and thus going further than previous reviews which have tended to focus on media portrayals of single health topics or media types (e.g. [296, 297]). As a result, this review was necessarily broad and our search strategy reflects this, for example in the decision to use a select subset of key MESH headings to capture articles in each of the topic areas rather than an exhaustive list of key words. As pointed out by one of the reviewers of this article, this may have resulted in the omission of relevant papers that used different terms from those contained in our search strategy. For example, it was noted that the work by Emery and colleagues on Twitter content related to tobacco use [298, 299] was not picked up within our search. However, a *post-hoc* deployment of our search strategy in Medline with the inclusion of additional search terms related to the original search terms for ‘smoking’ (addition terms: Tobacco Smoking/ OR Tobacco/ OR Tobacco, Smokeless/ OR Electronic Nicotine Delivery Systems/ OR Tobacco Products/ OR Vaping/ OR e-cig*.mp OR cigarette.mp OR juul.mp) and ‘social media’ (additional terms: facebook.mp OR twitter.mp OR Instagram.mp OR youtube.mp) only returned an additional 26 and 9 articles respectively (prior to any screening to assess whether these additional studies met the inclusion criteria). Similarly, we recognise that the decision to use ‘content analysis’ as search term (see Table 1) may have resulted in the omission of studies using different approaches such as discourse or textual analysis. However, the use of ‘frame’ and ‘framing’ as alongside ‘content analysis’ (see Table 1) meant that articles that examined framing of chronic disease issues using approaches other than content analysis were still captured within our search. Indeed, a post-hoc re-run of our search strategy with the addition of ‘discourse analysis’ and ‘text analysis’ in Medline, only returned an additional 34 results prior to any screening. As such, while a minority of papers may indeed have been missed as a result of our search strategy, this review still serves as a useful and novel snapshot of the literature, as intended when we set out to undertake a scoping review, and the current search strategy is unlikely to have significantly biased the findings.

The breadth of this review, spanning media coverage of a range of non-communicable diseases and their risk factors, meant that there was an extremely high volume of search results returned and articles included, which had implications for our handling of the data. First, due to the volume of results returned from the databases searches, and the intention for this review to be a ‘rapid mapping’ of key themes in this area, we did not extend the search to include unpublished literature or hand-searching of journals and recognise that this may have led to some studies being missed. Second, while it would have been desirable to have a second reviewer check all references for inclusion and data extraction, the volume of literature precluded this. Instead, we engaged in frequent discussions within the research team to ensure consistency and discuss uncertainties as they arose, and additional reviewers checked randomly selected subsets of data and demonstrated a high level of agreement (see ‘Study selection’). Finally, while more applicable to systematic reviews than scoping reviews, the large number of studies included within our sample meant that critical appraisal of the evidence and assessment of study quality was beyond the scope of this review.

Finally, the volume of studies identified within this review also presented challenges to data synthesis. For example, while we have identified a number of studies examining media portrayals of different policy interventions such as smoking regulation and sugar taxes, a more in depth synthesis of these papers to draw out similarities and differences in how different policies are framed within the news media and how this influences public opinion will be a valuable next step. Another insight that would be important to follow up is how risks, causes and solutions of chronic diseases have been framed across the topic areas in order to identify similarities and differences and the impacts of different framings across topics.

## Conclusions

This scoping review provides a high-level overview of the key topics, approaches and themes across existing research on media coverage of issues related to chronic disease spanning more than thirty years. Taken together, the findings of this review indicate that while there has been a considerable body of research on the amount and type of media coverage of issues related to chronic disease prevention, there has been less focus on the factors that influence the amount and type of media coverage, and the effects of media coverage on public attitudes and behaviours. While an understanding of how issues are framed within the news media is vital to understanding how stories around chronic disease are being told, greater understanding of the factors that influence how issues related to chronic disease prevention get reported and what audiences do with the information is needed going forwards. Further synthesis of study findings across different risk factors, causes and solutions, is also an important next step in order to demonstrate the key insights from the field as a whole that can be applied to aid understanding of future actions. For example, we recently conducted a synthesis of studies of the content and effects of media framing of a range of policy interventions for chronic disease prevention to inform an understanding of the how future policies might be portrayed in the media and responded to by the public [300]. Finally, while not the main focus of our search, we noted a steady increase in recent years in the number of articles considering the social determinants of health in relation to chronic disease prevention, which may represent an important shift towards recognising the key role that such factors play in shaping health.

## Supplementary information

**Additional file 1.** Characteristics of included papers.

**Additional file 2.** Preferred Reporting Items for Systematic reviews and Meta-Analyses extension for Scoping Reviews (PRISMA-ScR) Checklist.

## Data Availability

Not applicable
