# Supplementary material for: 30+ years of media analysis of relevance to chronic disease: a scoping review
Source: BMC Public Health. 2020 Mar 20;20:364. doi: 10.1186/s12889-020-8365-x (PMC7083065; doi:10.1186/s12889-020-8365-x)
Supplement: Supplementary file 1 — Additional file 1. Characteristics of included papers. [file 12889_2020_8365_MOESM1_ESM.docx]

| Author | Year | Country | Media category | | | | Methods | | | Health topic | | | | | | | | | | | Media Channel | | | | | | | | Focus | | | Sample start year | Sample end year | Sample timeframe |
| --- | --- | --- | --- | --- | --- | --- | --- | --- | --- | --- | --- | --- | --- | --- | --- | --- | --- | --- | --- | --- | --- | --- | --- | --- | --- | --- | --- | --- | --- | --- | --- | --- | --- | --- |
|  |  |  | **News** | **Marketing** | **Entertainment** | **Social Media** | **Descriptive** | **Experimental** | **Interview, survey or focus group** | **Alcohol** | **Smoking** | **Nutrition** | **Physical activity** | **Obesity** | **Cancer** | **Cardiovascular disease** | **Diabetes** | **Other chronic diseases** | **Other health topics** | **Social determinants** | **TV** | **Newspapers** | **Magazines** | **Radio** | **Movies** | **Music** | **Online** | **Other** | **Media representations** | **Effects of framing or exposure** | **Influences on reporting** |  |  |  |
| Abbatangelo-Gray, Byrd-Bredbenner and Austin [1] | 2008 | US |  | ✓ |  |  | ✓ |  |  |  |  | ✓ |  |  |  |  |  |  |  |  | ✓ |  |  |  |  |  |  |  | ✓ |  |  | 2003 | 2003 | <1 |
| Abel, Lee and Weeks [2] | 2007 | US |  | ✓ |  |  | ✓ |  |  |  |  |  |  |  | ✓ |  |  |  |  |  |  |  | ✓ |  |  |  |  |  | ✓ |  |  | 2003 | 2006 | 3 |
| Acevedo-Garcia, Barbeau, Bishop, Pan and Emmons [3] | 2004 | US |  | ✓ |  |  | ✓ |  |  |  | ✓ |  |  |  |  |  |  |  |  |  |  |  |  |  |  |  |  | ✓ | ✓ |  |  | 1970 | 2003 | 33 |
| Ackerson and Viswanath [4] | 2010 | US | ✓ |  |  |  | ✓ | ✓ |  |  |  |  |  |  | ✓ |  |  |  |  |  |  | ✓ |  |  |  |  |  |  | ✓ | ✓ |  | 2006 | 2006 | <1 |
| Akintola, Lavis and Hoskins [5] | 2015 | South Africa | ✓ |  |  |  | ✓ |  |  |  |  |  |  |  |  |  |  |  | ✓ | ✓ |  | ✓ |  |  |  |  |  |  | ✓ |  |  | 1997 | 2012 | 15 |
| Al-Naggar and Al-Jashamy [6] | 2011 | Malaysia | ✓ |  |  |  | ✓ |  |  |  |  |  |  |  | ✓ |  |  |  |  |  |  | ✓ |  |  |  |  |  |  | ✓ |  |  | 1997 | 2007 | 10 |
| Alvy and Calvert [7] | 2008 | US |  | ✓ |  |  | ✓ |  |  |  |  | ✓ |  |  |  |  |  |  |  |  |  |  |  |  |  |  | ✓ |  | ✓ |  |  | 2005 | 2005 | <1 |
| Andersson, Bjaras, Tillgren and Ostenson [8] | 2007 | Sweden | ✓ | ✓ |  |  | ✓ |  |  |  |  |  | ✓ |  |  |  |  |  |  |  |  | ✓ |  |  |  |  |  |  | ✓ |  |  | 1997 | 2002 | 5 |
| Andsager and Powers [10] | 2001 | US | ✓ |  |  |  | ✓ |  |  |  |  |  |  |  | ✓ |  |  |  |  |  |  |  | ✓ |  |  |  |  |  | ✓ |  |  | 1990 | 1997 | 7 |
| Andsager, Chen, Miles, Smith and Nothwehr [9] | 2015 | US | ✓ |  |  |  | ✓ |  |  |  |  | ✓ |  | ✓ |  |  |  |  |  |  |  | ✓ |  |  |  |  |  |  | ✓ |  |  | 2011 | 2012 | 1 |
| Arora [11] | 2000 | US |  | ✓ |  |  |  | ✓ |  |  |  |  |  |  |  |  |  |  | ✓ |  |  |  |  |  |  |  |  | ✓ |  | ✓ |  | N/A | N/A | N/A |
| Asbeek Brusse, Fransen and Smit [12] | 2015 | Netherlands |  |  | ✓ |  |  | ✓ | ✓ |  |  |  |  |  |  |  |  |  | ✓ |  | ✓ |  |  |  |  |  |  |  |  | ✓ |  | N/A | N/A | N/A |
| Athanasopoulou and Sakellari [13] | 2015 | Finland, Greece |  |  |  | ✓ | ✓ |  |  |  |  |  |  |  |  |  |  |  | ✓ |  |  |  |  |  |  |  | ✓ |  | ✓ |  |  | 2015 | 2015 | <1 |
| Atkin, Smith, McFeters and Ferguson [14] | 2008 | US | ✓ |  |  |  | ✓ |  |  |  |  |  |  |  | ✓ |  |  |  |  |  | ✓ | ✓ | ✓ |  |  |  |  |  | ✓ |  |  | 2002 | 2004 | 2 |
| Atkinson, Sumnall and Measham [15] | 2011 | UK |  |  | ✓ | ✓ | ✓ |  |  | ✓ |  |  |  |  |  |  |  |  |  |  | ✓ |  |  |  |  |  | ✓ |  | ✓ | ✓ |  | 2009 | 2009 | <1 |
| Aubrey [16] | 2010 | US | ✓ |  |  |  | ✓ | ✓ |  |  |  | ✓ | ✓ | ✓ |  |  |  |  | ✓ |  |  |  | ✓ |  |  |  |  |  | ✓ | ✓ |  | 2003 | 2008 | 5 |
| Aubrey and Hahn [17] | 2016 | US | ✓ |  |  |  | ✓ |  |  |  |  | ✓ | ✓ | ✓ |  |  |  |  | ✓ |  |  |  | ✓ |  |  |  |  |  | ✓ |  |  | 2003 | 2007 | 4 |
| Austin, Pinkleton and Fujioka [18] | 1999 | US |  | ✓ |  |  |  | ✓ |  | ✓ |  |  |  |  |  |  |  |  |  |  | ✓ |  |  |  |  |  |  |  |  | ✓ |  | N/A | N/A | N/A |
| Azar, White, Bland, Livingston, Room, Chikritzhs, Durkin, Gilmore and Wakefield [19] | 2014 | Australia | ✓ |  |  |  | ✓ |  |  | ✓ |  |  |  |  |  |  |  |  |  |  |  | ✓ |  |  |  |  |  |  | ✓ |  |  | 2000 | 2011 | 11 |
| Babooram, Mullan and Sharpe [20] | 2010 | Australia |  | ✓ |  |  |  |  | ✓ |  |  | ✓ | ✓ | ✓ |  |  |  |  |  |  | ✓ |  |  |  |  |  |  | ✓ |  | ✓ |  | N/A | N/A | N/A |
| Bach, Shelton, Moreland-Russell and Israel [21] | 2013 | US | ✓ |  |  |  | ✓ |  | ✓ |  | ✓ |  |  |  |  |  |  |  |  |  |  | ✓ |  |  |  |  |  |  | ✓ |  |  | 2010 | 2011 | 1 |
| Backstrom [22] | 2012 | US |  |  | ✓ |  | ✓ |  |  |  |  |  |  | ✓ |  |  |  |  |  |  | ✓ |  |  |  |  |  |  |  | ✓ |  |  | N/A | N/A | N/A |
| Baek and Mayer [23] | 2010 | US |  | ✓ |  |  | ✓ |  |  |  | ✓ |  |  |  |  |  |  |  |  |  |  |  | ✓ |  |  |  |  |  | ✓ |  |  | 1994 | 2003 | 9 |
| Balbach and Glantz [24] | 1995 | US | ✓ |  |  |  | ✓ |  |  |  | ✓ |  |  |  |  |  |  |  |  |  |  |  | ✓ |  |  |  |  |  | ✓ |  |  | 1989 | 1994 | 5 |
| Balbach, Herzberg and Barbeau [25] | 2006 | US | ✓ |  |  |  | ✓ |  |  |  | ✓ |  |  |  |  |  |  |  |  |  |  |  |  |  |  |  |  | ✓ | ✓ |  |  | N/A | N/A | N/A |
| Bannon and Schwartz [26] | 2006 | US |  | ✓ |  |  |  | ✓ |  |  |  | ✓ |  |  |  |  |  |  |  |  |  |  |  |  |  |  |  | ✓ |  | ✓ |  | N/A | N/A | N/A |
| Bansal, John and Ling [27] | 2005 | India |  | ✓ |  |  | ✓ |  |  |  | ✓ |  |  |  |  |  |  |  |  |  | ✓ | ✓ | ✓ |  | ✓ |  | ✓ | ✓ | ✓ |  |  | 2003 | 2003 | <1 |
| Barker, McNeir, Sameer and Russell [28] | 2014 | UK | ✓ | ✓ |  |  | ✓ |  |  |  |  | ✓ |  |  |  |  |  |  |  |  |  |  | ✓ |  |  |  |  |  | ✓ |  |  | 1950 | 1998 | 48 |
| Barry, Brescoll and Gollust [29] | 2013 | US | ✓ |  |  |  | ✓ | ✓ |  |  |  |  |  | ✓ |  |  |  |  |  |  | ✓ | ✓ | ✓ |  |  |  |  |  | ✓ | ✓ |  | 1999 | 2008 | 9 |
| Barry, Jarlenski, Grob, Schlesinger and Gollust [30] | 2011 | US | ✓ |  |  |  | ✓ |  |  |  |  |  |  | ✓ |  |  |  |  |  |  | ✓ | ✓ | ✓ |  |  |  |  |  | ✓ |  |  | 2000 | 2009 | 9 |
| Basil, Basil and Schooler [31] | 2000 | US |  | ✓ |  |  | ✓ |  |  |  | ✓ |  |  |  |  |  |  |  |  |  |  |  | ✓ |  |  |  |  |  | ✓ |  |  | 1965 | 1995 | 30 |
| Baskin, Herbey, Williams, Ard, Ivankova and Odoms-Young [32] | 2013 | US |  | ✓ |  |  |  |  | ✓ |  |  | ✓ |  | ✓ |  |  |  |  |  |  |  |  |  |  |  |  |  |  |  | ✓ |  | N/A | N/A | N/A |
| Bassett-Gunter, Martin Ginis and Latimer-Cheung [33] | 2013 | Canada |  | ✓ |  |  |  | ✓ |  |  |  |  | ✓ |  |  |  |  |  |  |  |  |  |  |  |  |  |  | ✓ |  | ✓ |  | N/A | N/A | N/A |
| Beaudoin [34] | 2002 | US |  | ✓ |  |  | ✓ |  |  |  | ✓ |  |  |  |  |  |  |  |  |  | ✓ |  |  |  |  |  |  |  | ✓ |  |  | 1991 | 1999 | 8 |
| Bell [35] | 2014 | Canada | ✓ |  |  |  | ✓ |  |  |  | ✓ |  |  |  |  |  |  |  |  |  | ✓ |  |  |  |  |  |  |  | ✓ |  |  | 1980 | 1980 | <1 |
| Bell, Berger, Cassady and Townsend [36] | 2005 | US |  |  | ✓ |  | ✓ |  |  | ✓ |  | ✓ | ✓ |  |  |  |  |  |  |  |  |  |  |  | ✓ |  |  |  | ✓ |  |  | 1991 | 2000 | 9 |
| Belstock, Connolly, Carpenter and Tucker [37] | 2008 | US |  | ✓ |  |  | ✓ |  |  | ✓ | ✓ |  |  |  |  |  |  |  |  |  |  |  | ✓ |  |  |  |  |  | ✓ |  |  | 2003 | 2004 | 1 |
| Berenbaum and Latimer-Cheung [38] | 2014 | Canada |  | ✓ |  |  |  | ✓ |  |  |  |  | ✓ |  |  |  |  |  |  |  |  |  |  |  |  |  |  | ✓ |  | ✓ |  | N/A | N/A | N/A |
| Bergamini, Demidenko and Sargent [39] | 2013 | US |  | ✓ | ✓ |  | ✓ |  |  |  | ✓ |  |  |  |  |  |  |  |  |  |  |  |  |  | ✓ |  |  |  | ✓ |  |  | 1998 | 2009 | 11 |
| Berry, McCarville and Rhodes [40] | 2007 | Canada | ✓ |  |  |  | ✓ |  |  | ✓ | ✓ | ✓ | ✓ | ✓ | ✓ | ✓ | ✓ |  | ✓ |  |  | ✓ |  | ✓ |  |  | ✓ |  | ✓ |  |  | 1999 | 2003 | 4 |
| Berry, Stearns, Courneya, McGannon, Norris, Rodgers and Spence [41] | 2016 | Canada | ✓ | ✓ |  |  | ✓ |  | ✓ |  |  |  |  |  | ✓ | ✓ |  |  |  |  |  | ✓ | ✓ |  |  |  |  |  | ✓ | ✓ |  | 2012 | 2013 | <1 |
| Berry, Wharf-Higgins and Naylor [42] | 2008 | Canada |  | ✓ |  |  | ✓ |  |  |  |  |  | ✓ |  |  |  |  |  |  |  |  |  | ✓ |  |  |  |  |  | ✓ |  |  | 2005 | 2005 | <1 |
| Beullens and Schepers [43] | 2013 | US and Belgium |  |  |  | ✓ | ✓ |  |  | ✓ |  |  |  |  |  |  |  |  |  |  |  |  |  |  |  |  | ✓ |  | ✓ | ✓ |  | N/A | N/A | N/A |
| Bie and Tang [44] | 2015 | China | ✓ |  |  |  | ✓ |  |  |  |  |  |  |  |  |  |  |  | ✓ |  |  | ✓ |  |  |  |  |  |  | ✓ |  |  | 2003 | 2012 | 9 |
| Bigman [45] | 2014 | US | ✓ |  |  |  |  | ✓ |  |  |  |  |  |  | ✓ |  |  |  | ✓ |  |  |  |  |  |  |  |  | ✓ |  | ✓ |  | N/A | N/A | N/A |
| Bissonnette-Maheux, Provencher, Lapointe, Dugrenier, Dumas, Pluye, Straus, Gagnon and Desroches [46] | 2015 | Canada |  |  |  | ✓ |  |  | ✓ |  |  | ✓ |  |  |  |  |  |  |  |  |  |  |  |  |  |  | ✓ |  |  | ✓ |  | 2013 | 2013 | <1 |
| Blake, Kaufman, Lorenzo and Augustson [47] | 2015 | US | ✓ |  |  |  | ✓ |  |  |  | ✓ |  |  |  |  |  |  |  |  |  | ✓ |  |  |  |  |  |  |  | ✓ |  |  | 2008 | 2009 | 1 |
| Bleakley, Romer and Jamieson [48] | 2014 | US |  |  | ✓ |  | ✓ |  |  | ✓ | ✓ |  |  |  |  |  |  |  |  |  |  |  |  |  | ✓ |  |  |  | ✓ |  |  | 1985 | 2010 | 25 |
| Boepple and Thompson [50] | 2014 | US |  |  |  | ✓ | ✓ |  |  |  |  | ✓ | ✓ | ✓ |  |  |  |  |  |  |  |  |  |  |  |  | ✓ |  | ✓ |  |  | N/A | N/A | N/A |
| Boepple, Ata, Rum and Thompson [49] | 2016 | US |  |  |  | ✓ | ✓ |  |  |  |  | ✓ | ✓ | ✓ |  |  |  |  |  |  |  |  |  |  |  |  | ✓ |  | ✓ |  |  | N/A | N/A | N/A |
| Boessen and Maarse [51] | 2008 | Netherlands |  | ✓ |  |  | ✓ |  | ✓ |  | ✓ |  |  |  | ✓ |  |  |  |  |  |  |  |  |  |  |  |  | ✓ | ✓ |  |  | 2005 | 2006 | 1 |
| Bonfiglioli, Hattersley and King [52] | 2011 | Australia | ✓ |  |  |  | ✓ |  |  |  |  | ✓ |  |  |  |  |  |  |  |  | ✓ | ✓ |  |  |  |  |  |  | ✓ |  |  | 2007 | 2007 | <1 |
| Bonfiglioli, Smith, King, Chapman and Holding [53] | 2007 | Australia | ✓ |  |  |  | ✓ |  |  |  |  |  |  | ✓ |  |  |  |  |  |  | ✓ | ✓ |  |  |  |  |  |  | ✓ |  |  | 2005 | 2005 | <1 |
| Bonilha Dubugras, Evans-Lacko and de Jesus Mari [54] | 2011 | Brazil | ✓ |  |  |  | ✓ |  |  |  |  |  |  |  |  |  |  |  | ✓ |  |  | ✓ |  |  |  |  |  |  | ✓ |  |  | 2007 | 2008 | 1 |
| Borra, Earl and Hogan [55] | 1998 | US | ✓ |  |  |  | ✓ |  |  |  |  | ✓ |  |  |  |  |  |  |  |  |  | ✓ |  |  |  |  | ✓ |  | ✓ |  |  | 1995 | 1995 | <1 |
| Bosone, Martinez and Kalampalikis [56] | 2015 | France |  | ✓ |  |  |  | ✓ |  |  |  | ✓ |  |  |  |  |  |  |  |  |  |  |  |  |  |  |  | ✓ |  | ✓ |  | N/A | N/A | N/A |
| Bouman [57] | 2002 | Netherlands |  |  | ✓ |  |  |  | ✓ |  |  |  |  |  |  |  |  |  | ✓ |  | ✓ |  |  |  |  |  |  |  |  |  | ✓ | N/A | N/A | N/A |
| Bragg, Liu, Roberto, Sarda, Harris and Brownell [58] | 2013 | US |  | ✓ |  |  | ✓ |  |  |  |  | ✓ | ✓ |  |  |  |  |  |  |  |  |  |  |  |  |  |  | ✓ | ✓ |  |  | 2010 | 2010 | <1 |
| Brannstrom and Lindblad [59] | 1994 | Sweden | ✓ |  |  |  | ✓ |  | ✓ |  |  |  |  |  |  | ✓ | ✓ |  |  |  | ✓ | ✓ |  | ✓ |  |  |  |  | ✓ | ✓ |  | 1985 | 1990 | 5 |
| Brown-Johnson, Sanders-Jackson and Prochaska [60] | 2014 | US | ✓ |  |  | ✓ | ✓ |  |  |  | ✓ |  |  |  |  |  |  |  | ✓ |  |  |  |  |  |  |  | ✓ |  | ✓ |  |  | 2013 | 2014 | 1 |
| Brownson, Mack, Meegama, Pratt, Brownson, Dean, Dabney and Luke [61] | 1996 | USA | ✓ |  |  |  | ✓ | ✓ |  |  |  |  |  |  |  | ✓ |  |  |  |  |  | ✓ |  |  |  |  |  |  | ✓ |  |  | 1988 | 1993 | 5 |
| Buis and Carpenter [62] | 2009 | US |  |  |  | ✓ | ✓ |  |  |  |  |  |  |  |  |  |  |  | ✓ |  |  |  |  |  |  |  | ✓ |  | ✓ |  |  | 2006 | 2006 | <1 |
| Byrd-Bredbenner [63] | 2004 | US |  |  | ✓ |  | ✓ |  |  |  |  |  |  |  |  |  |  |  | ✓ |  | ✓ |  |  |  |  |  |  |  | ✓ |  |  | 1989 | 1995 | 6 |
| Byrne, Niederdeppe, Avery and Cantor [64] | 2013 | US |  | ✓ |  |  | ✓ |  |  |  |  |  |  |  |  |  |  | ✓ |  |  | ✓ |  | ✓ |  |  |  |  |  | ✓ |  |  | 1994 | 2007 | 13 |
| Caburnay, Kreuter, Cameron, Luke, Cohen, McDaniels, Wohlberg and Atkins [65] | 2008 | US | ✓ |  |  |  | ✓ |  | ✓ |  |  |  |  |  | ✓ |  |  |  | ✓ |  |  | ✓ |  |  |  |  |  |  | ✓ | ✓ |  | 2004 | 2005 | 2 |
| Cai, Yang, Liu, Ma and Liu [66] | 2009 | China | ✓ |  |  |  | ✓ |  |  |  |  |  |  |  | ✓ |  |  |  |  |  |  | ✓ |  |  |  |  |  |  | ✓ |  |  | 2000 | 2007 | 7 |
| Campo and Mastin [67] | 2007 | US | ✓ |  |  |  | ✓ |  |  |  |  |  |  | ✓ |  |  |  |  |  |  |  |  | ✓ |  |  |  |  |  | ✓ |  |  | 1984 | 2004 | 20 |
| Cardador, Hazan and Glantz [68] | 1995 | US | ✓ |  |  |  | ✓ |  |  |  | ✓ |  |  |  |  |  |  |  |  |  |  |  |  |  |  |  |  | ✓ | ✓ |  |  | 1987 | 1992 | 5 |
| Carew, Kutcher, Wei and McLuckie [69] | 2014 | Canada |  |  |  | ✓ | ✓ |  |  |  |  |  |  |  |  |  |  |  | ✓ |  |  |  |  |  |  |  | ✓ |  | ✓ |  |  | 2006 | 2010 | 4 |
| Carroll and Freeman [70] | 2015 | Australia | ✓ |  |  |  | ✓ |  |  |  | ✓ |  |  |  |  |  |  |  |  |  |  |  |  |  |  |  | ✓ |  | ✓ |  |  | 2013 | 2013 | <1 |
| Carter [71] | 2003 | Australia |  | ✓ |  |  | ✓ |  |  |  | ✓ |  |  |  |  |  |  |  |  |  |  |  |  |  |  |  | ✓ |  | ✓ |  |  | N/A | N/A | N/A |
| Cash, Thelwall, Peck, Ferrell and Bridge [72] | 2013 | US |  |  |  | ✓ | ✓ |  |  |  |  |  |  |  |  |  |  |  | ✓ |  |  |  |  |  |  |  | ✓ |  | ✓ |  |  | 2008 | 2008 | <1 |
| Castonguay [73] | 2015 | US |  | ✓ |  |  | ✓ | ✓ | ✓ |  |  | ✓ | ✓ |  |  |  |  |  |  |  | ✓ |  |  |  |  |  |  |  | ✓ | ✓ |  | 2009 | 2013 | 4 |
| Castonguay, McKinley and Kunkel [74] | 2013 | US |  | ✓ |  |  | ✓ |  |  |  |  | ✓ |  |  |  |  |  |  |  |  | ✓ |  |  |  |  |  |  |  | ✓ |  |  | 2009 | 2009 | <1 |
| Champion and Chapman [75] | 2005 | Australia | ✓ |  |  |  | ✓ |  |  |  | ✓ |  |  |  |  |  |  |  |  |  |  | ✓ |  |  |  |  |  |  | ✓ |  |  | 1996 | 2003 | 7 |
| Chan, Patch and Williams [76] | 2005 | Australia |  | ✓ |  |  |  |  | ✓ |  |  | ✓ |  |  |  |  |  |  |  |  |  |  |  |  |  |  |  |  |  | ✓ |  | N/A | N/A | N/A |
| Chang [77] | 2005 | Taiwan |  | ✓ |  |  | ✓ |  | ✓ |  | ✓ |  |  |  |  |  |  |  |  |  |  |  | ✓ |  |  |  |  |  | ✓ |  |  | 2003 | 2003 | <1 |
| Chang [78] | 2006 | Taiwan |  | ✓ |  |  |  | ✓ |  |  | ✓ |  |  |  |  |  |  |  |  |  |  |  |  |  |  |  |  | ✓ |  | ✓ |  | N/A | N/A | N/A |
| Chang [79] | 2007 | Taiwan |  | ✓ |  |  | ✓ |  | ✓ |  | ✓ |  |  |  |  |  |  |  |  |  |  |  | ✓ |  |  |  |  |  | ✓ |  |  | 2001 | 2003 | 2 |
| Chapman, Nicholas and Supramaniam [80] | 2006 | Australia |  | ✓ |  |  | ✓ |  |  |  |  | ✓ |  |  |  |  |  |  |  |  | ✓ |  |  |  |  |  |  |  | ✓ |  |  | 2005 | 2005 | <1 |
| Chen, Eborall and Armstrong [81] | 2014 | UK | ✓ |  |  |  | ✓ |  |  |  |  |  |  |  | ✓ |  |  |  |  |  |  | ✓ |  |  |  |  |  | ✓ |  |  |  | 2010 | 2011 | 2 |
| Choi and Kim [83] | 2011 | Korea |  | ✓ |  |  | ✓ |  |  |  |  | ✓ |  |  |  |  |  |  |  |  | ✓ |  |  |  |  |  |  |  | ✓ |  |  | 2005 | 2007 | 2 |
| Choi, Fabian, Jansen, Lenk and Forster [82] | 2013 | US |  | ✓ | ✓ |  |  |  | ✓ |  | ✓ |  |  |  |  |  |  |  |  |  |  |  | ✓ |  | ✓ |  |  |  |  | ✓ |  | 2010 | 2011 | 1 |
| Christenson, Roberts and Bjork [84] | 2012 | US |  |  | ✓ |  | ✓ |  |  | ✓ |  |  |  |  |  |  |  |  |  |  |  |  |  |  |  | ✓ |  |  | ✓ |  |  | 1968 | 2008 | 40 |
| Chu, Unger, Allem, Pattarroyo, Soto, Cruz, Yang, Jiang and Yang [85] | 2015 | US |  | ✓ |  |  | ✓ |  |  |  | ✓ |  |  |  |  |  |  |  |  |  |  |  |  |  |  |  | ✓ |  | ✓ |  |  | 2014 | 2014 | <1 |
| Chung [86] | 2015 | US |  |  |  | ✓ | ✓ |  |  |  | ✓ |  |  |  |  |  |  |  |  |  |  |  |  |  |  |  | ✓ |  | ✓ | ✓ |  | 2012 | 2012 | <1 |
| Clarke [89] | 1992 | Canada | ✓ |  |  |  | ✓ |  |  |  |  |  |  |  | ✓ | ✓ |  |  | ✓ |  |  |  | ✓ |  |  |  |  |  | ✓ |  |  | 1961 | 1985 | 25 |
| Clarke [90] | 2006 | US & Canada | ✓ |  |  |  | ✓ |  |  |  |  |  |  |  |  |  |  |  | ✓ |  |  |  | ✓ |  |  |  |  |  | ✓ |  |  | 1997 | 2001 | 4 |
| Clarke and Everest [91] | 2006 | Canada | ✓ |  |  |  | ✓ |  |  |  |  |  |  |  | ✓ |  |  |  |  |  |  |  | ✓ |  |  |  |  |  | ✓ |  |  | 2001 | 2010 | 10 |
| Clarke and Mosleh [92] | 2015 | US | ✓ |  |  |  | ✓ |  |  |  |  |  |  |  |  |  |  |  | ✓ | ✓ |  |  | ✓ |  |  |  |  |  | ✓ |  |  | 1990 | 2012 | 22 |
| Clarke and van Amerom [87] | 2008 | US, Canada | ✓ |  |  |  | ✓ |  |  |  |  |  |  |  | ✓ | ✓ |  |  |  | ✓ |  |  | ✓ |  |  |  |  |  | ✓ |  |  | 2001 | 2001 | <1 |
| Clarke, van Amerom and Binns [88] | 2007 | Canada | ✓ |  |  |  | ✓ |  |  |  |  |  |  |  |  | ✓ |  |  |  |  |  |  | ✓ |  |  |  |  |  | ✓ |  |  | 1991 | 2001 | 10 |
| Cobb, Mays and Graham [93] | 2013 | US |  |  |  | ✓ | ✓ |  |  |  | ✓ |  |  |  |  |  |  |  |  |  |  |  |  |  |  |  | ✓ |  | ✓ | ✓ |  | 2005 | 2008 | 3 |
| Cohen, Caburnay, Luke, Rodgers, Cameron and Kreuter [94] | 2008 | US | ✓ |  |  |  | ✓ |  |  |  |  |  |  |  | ✓ |  |  |  |  | ✓ |  | ✓ |  |  |  |  |  |  | ✓ |  |  | 2004 | 2005 | 1 |
| Cohen, Shumate and Gold [95] | 2007 | US |  | ✓ |  |  | ✓ |  |  |  | ✓ |  |  |  |  |  |  |  |  |  | ✓ |  |  |  |  |  |  |  | ✓ |  |  | 2004 | 2004 | <1 |
| Cokkinides, Kirkland, Andrews, Sullivan and Lichtenfeld [96] | 2012 | US | ✓ |  |  |  | ✓ |  |  |  |  |  |  |  | ✓ |  |  |  |  |  |  | ✓ | ✓ |  |  |  |  |  | ✓ |  |  | 2009 | 2009 | <1 |
| Cole-Lewis, Perotte, Galica, Dreyer, Griffith, et al. [97] | 2016 | US |  |  |  | ✓ | ✓ |  |  |  |  |  |  |  | ✓ |  |  |  |  |  |  |  |  |  |  |  | ✓ |  | ✓ |  |  | 2013 | 2014 | 1 |
| Cole-Lewis, Pugatch, Sanders, Varghese, Posada, Yun, Schwarz and Augustson [98] | 2015 | US |  |  |  | ✓ | ✓ |  |  |  | ✓ |  |  |  |  |  |  |  |  |  |  |  |  |  |  |  | ✓ |  | ✓ |  |  | 2013 | 2014 | 1 |
| Cole-Lewis, Varghese, Sanders, Schwarz, Pugatch and Augustson [99] | 2015 | US |  |  |  | ✓ | ✓ |  |  |  | ✓ |  |  |  |  |  |  |  |  |  |  |  |  |  |  |  | ✓ |  | ✓ |  |  | 2013 | 2014 | 1 |
| Coleman, Thorson and Wilkins [100] | 2011 | US | ✓ |  |  |  |  | ✓ |  |  | ✓ |  |  | ✓ |  |  | ✓ |  |  |  |  |  |  |  |  |  |  | ✓ |  | ✓ |  | N/A | N/A | N/A |
| Commers, Visser and De Leeuw [101] | 2000 | Netherlands | ✓ | ✓ |  |  | ✓ |  |  |  |  |  |  |  |  |  |  |  | ✓ |  |  | ✓ |  |  |  |  |  |  | ✓ |  |  | 1998 | 1998 | <1 |
| Connor [102] | 2006 | US |  | ✓ |  |  | ✓ |  |  |  |  | ✓ |  |  |  |  |  |  |  |  | ✓ |  |  |  |  |  |  |  | ✓ |  |  | 2005 | 2005 | <1 |
| Cooper, Burgoon and Roter [103] | 2001 | US | ✓ |  |  |  |  | ✓ |  |  |  |  |  |  |  |  |  | ✓ |  |  |  |  |  |  |  |  |  | ✓ |  | ✓ |  | N/A | N/A | N/A |
| Cooper, Roter and Langlieb [104] | 2000 | US | ✓ |  | ✓ |  |  | ✓ |  |  |  |  |  |  |  |  |  | ✓ | ✓ |  | ✓ |  |  |  |  |  |  |  |  | ✓ |  | N/A | N/A | N/A |
| Cortese, Lewis and Ling [105] | 2009 | US |  | ✓ |  |  | ✓ |  |  |  | ✓ |  |  |  |  |  |  |  |  |  |  |  | ✓ |  |  |  |  |  | ✓ |  |  | 2006 | 2007 | 1 |
| Covello and Peters [106] | 2002 | US | ✓ |  |  |  | ✓ |  | ✓ |  |  |  |  |  | ✓ | ✓ |  | ✓ |  |  | ✓ | ✓ | ✓ | ✓ |  |  |  |  | ✓ | ✓ |  | 1997 | 1997 | <1 |
| Cranwell, Murray, Lewis, Leonardi-Bee, Dockrell and Britton [107] | 2015 | UK |  |  | ✓ |  | ✓ |  | ✓ | ✓ | ✓ |  |  |  |  |  |  |  |  |  |  |  |  |  |  | ✓ | ✓ |  | ✓ | ✓ |  | 2013 | 2014 | <1 |
| Culp, Bell and Cassady [108] | 2010 | US |  | ✓ |  |  | ✓ |  |  |  |  | ✓ |  |  |  |  |  |  |  |  | ✓ |  |  |  |  |  | ✓ |  | ✓ |  |  | 2006 | 2007 | 1 |
| Dal Cin, Worth, Dalton and Sargent [109] | 2008 | US |  |  | ✓ |  | ✓ |  | ✓ | ✓ |  |  |  |  |  |  |  |  |  |  |  |  |  |  | ✓ |  |  |  | ✓ |  |  | 1998 | 2003 | 5 |
| Daykin, Irwin, Kimberlee, Orme, Plant, McCarron and Rahbari [110] | 2009 | UK |  |  | ✓ |  | ✓ |  |  | ✓ |  |  |  |  |  |  |  |  |  |  |  |  |  | ✓ |  |  |  |  | ✓ |  |  | 2007 | 2007 | <1 |
| De Brun, McCarthy, McKenzie and McGloin [111] | 2015 | Ireland | ✓ |  |  |  | ✓ |  |  |  |  |  |  | ✓ |  |  |  |  |  |  |  | ✓ |  | ✓ |  |  | ✓ |  | ✓ |  |  | 2005 | 2009 | 4 |
| De Brun, McKenzie, McCarthy and McGloin [112] | 2012 | Ireland | ✓ |  |  |  | ✓ |  |  |  |  |  |  | ✓ |  |  |  |  |  |  |  | ✓ |  |  |  |  |  |  | ✓ |  |  | 1997 | 2009 | 12 |
| De la Torre-Diez, Diaz-Pernas and Anton-Rodriguez [113] | 2012 | Spain |  | ✓ |  | ✓ | ✓ |  |  |  |  |  |  |  | ✓ |  | ✓ |  |  |  |  |  |  |  |  |  | ✓ |  | ✓ |  |  | 2011 | 2011 | <1 |
| DeJong [114] | 1996 | US | ✓ |  |  |  | ✓ |  |  |  | ✓ |  |  |  |  |  |  |  |  |  |  | ✓ |  |  |  |  |  |  | ✓ |  |  | 1988 | 1995 | 7 |
| DeJong and Atkin [115] | 1995 | US |  | ✓ |  |  | ✓ |  |  | ✓ |  |  |  |  |  |  |  |  |  |  | ✓ |  |  |  |  |  |  |  | ✓ |  |  | 1987 | 1992 | 5 |
| DeJong and Hoffman [116] | 2000 | US |  | ✓ |  |  | ✓ |  |  |  | ✓ |  |  |  |  |  |  |  |  |  | ✓ |  |  |  |  |  |  |  | ✓ |  |  | 1993 | 1996 | 3 |
| Dewe, Ogden and Coyle [117] | 2015 | UK |  | ✓ |  |  | ✓ |  |  |  | ✓ |  |  |  |  |  |  |  |  |  |  |  |  |  |  |  |  | ✓ | ✓ |  |  | 1950 | 2003 | 53 |
| Dixon, Warne, Scully, Dobbinson and Wakefield [118] | 2014 | Australia | ✓ |  |  |  | ✓ |  | ✓ |  |  |  |  |  | ✓ |  |  |  |  |  |  | ✓ |  |  |  |  |  |  | ✓ | ✓ |  | 1994 | 2007 | 14 |
| Dobias, Moyer, McAchran, Katz and Sonnad [119] | 2001 | US | ✓ |  |  |  | ✓ |  |  |  |  |  |  |  | ✓ |  |  |  |  |  |  |  | ✓ |  |  |  |  |  | ✓ |  |  | 1988 | 1994 | 6 |
| Dodgson, Tarrant, Thompson and Young [120] | 2008 | Hong Kong | ✓ |  |  |  | ✓ |  |  |  |  | ✓ |  |  |  |  |  |  | ✓ |  |  | ✓ | ✓ |  |  |  |  |  | ✓ |  |  | 1999 | 2003 | 4 |
| Donaldson, Cohen, Truant, Rutkow, Kanarek and Barry [121] | 2015 | US | ✓ |  |  |  | ✓ |  |  |  |  | ✓ |  |  |  |  |  |  |  |  | ✓ | ✓ |  |  |  |  |  |  | ✓ |  |  | 2012 | 2013 | 1 |
| Donelle and Booth [122] | 2012 | US |  |  |  | ✓ | ✓ |  |  |  |  |  |  |  |  |  |  |  | ✓ |  |  |  |  |  |  |  | ✓ |  | ✓ |  |  | 2009 | 2009 | <1 |
| Donelle, Hoffman-Goetz and Clarke [123] | 2004 | Canada | ✓ |  |  |  | ✓ |  |  |  |  |  |  |  | ✓ |  |  |  |  |  |  | ✓ |  |  |  |  |  |  | ✓ |  |  | 1996 | 2000 | 5 |
| Donelle, Hoffman-Goetz and Clarke [124] | 2005 | Canada | ✓ |  |  |  | ✓ |  |  |  |  |  |  |  | ✓ |  |  |  |  |  |  | ✓ |  |  |  |  |  |  | ✓ |  |  | 1996 | 2000 | 4 |
| Doub, Small and Birch [125] | 2016 | US |  |  |  | ✓ | ✓ |  |  |  |  | ✓ |  |  |  |  |  |  | ✓ |  |  |  |  |  |  |  | ✓ |  | ✓ |  |  | 2013 | 2013 | <1 |
| Duckworth, Halpern, Schutt and Gillespie [126] | 2003 | US | ✓ |  |  |  | ✓ |  |  |  |  |  |  |  | ✓ |  |  |  | ✓ |  |  | ✓ |  |  |  |  |  |  | ✓ |  |  | 1996 | 1997 | 1 |
| Durrant, Rome, Rich, Allred, Emans and Woods [128] | 1997 | US |  |  | ✓ |  | ✓ |  |  |  | ✓ |  |  |  |  |  |  |  |  |  |  |  |  |  |  | ✓ |  |  | ✓ |  |  | 1994 | 1994 | <1 |
| Durrant, Wakefield, McLeod, Clegg-Smith and Chapman [127] | 2003 | Australia | ✓ |  |  |  | ✓ |  |  |  | ✓ |  |  |  |  |  |  |  |  |  |  | ✓ |  |  |  |  |  |  | ✓ |  |  | 2001 | 2001 | <1 |
| Earl and Wellman [129] | 1997 | US | ✓ |  |  |  | ✓ |  |  |  |  | ✓ |  |  |  |  |  |  |  |  | ✓ | ✓ |  |  |  |  |  |  | ✓ |  |  | 1995 | 1995 | <1 |
| Easson, Agarwal, Duda and Bennett [130] | 2014 | Canada | ✓ |  |  |  | ✓ |  |  |  |  |  |  |  |  |  |  |  | ✓ |  |  | ✓ |  |  |  |  |  |  | ✓ |  |  | 2008 | 2012 | 4 |
| Eisenberg, Carlson-McGuire, Gollust and Neumark-Sztainer [131] | 2015 | US |  |  | ✓ |  | ✓ |  | ✓ |  |  |  |  | ✓ |  |  |  |  |  |  | ✓ |  |  |  |  |  |  |  | ✓ |  |  | 2009 | 2010 | 1 |
| Eisenberg, Larson, Gollust and Neumark-Sztainer [132] | 2016 | US |  |  | ✓ |  | ✓ |  | ✓ |  |  | ✓ |  |  |  |  |  |  |  |  | ✓ |  |  |  |  |  |  |  | ✓ |  |  | 2010 | 2010 | <1 |
| Elstad, Sheridan, Lee, Rini, Earp and Brewer [133] | 2014 | US | ✓ |  |  |  | ✓ |  |  |  |  |  |  |  | ✓ |  |  |  |  |  |  | ✓ |  |  |  |  |  |  | ✓ |  |  | 2005 | 2012 | 8 |
| Emond, Smith, Mathur, Sargent and Gilbert-Diamond [134] | 2015 | US |  | ✓ |  |  | ✓ |  |  |  |  | ✓ |  |  |  |  |  |  |  |  | ✓ |  |  |  |  |  |  |  | ✓ |  |  | 2012 | 2013 | 1 |
| Eslick and Eslick [135] | 2009 | US |  |  | ✓ |  | ✓ |  |  |  | ✓ |  |  |  |  |  |  |  |  |  | ✓ |  |  |  |  |  |  |  | ✓ |  |  | 2008 | 2008 | <1 |
| Eyal and Te'eni-Harari [136] | 2016 | Israel |  | ✓ |  |  | ✓ |  |  |  |  | ✓ |  |  |  |  |  |  |  |  | ✓ |  |  |  |  |  |  |  | ✓ |  |  | 2008 | 2013 | 5 |
| Fagan, Lyons and Smyth [137] | 2014 | Ireland | ✓ |  |  |  | ✓ |  |  | ✓ |  |  |  |  |  |  |  |  |  |  |  | ✓ |  |  |  |  |  |  | ✓ |  |  | 2008 | 2009 | 1 |
| Faulkner, Finlay and Roy [138] | 2007 | Canada | ✓ |  |  |  | ✓ |  |  |  |  |  | ✓ |  |  |  |  |  |  |  |  | ✓ |  |  |  |  |  |  | ✓ |  |  | 2004 | 2005 | 1 |
| Fishman, Ten Have and Casarett [139] | 2010 | US | ✓ |  |  |  | ✓ |  |  |  |  |  |  |  | ✓ |  |  |  |  |  |  | ✓ | ✓ |  |  |  |  |  | ✓ |  |  | 2005 | 2007 | 3 |
| Fishman, Ten Have and Casarett [140] | 2012 | US | ✓ |  |  |  | ✓ |  |  |  |  |  |  |  | ✓ |  |  |  |  | ✓ |  | ✓ | ✓ |  |  |  |  |  | ✓ |  |  | 2003 | 2010 | 8 |
| Fogarty and Chapman [141] | 2011 | Australia | ✓ |  |  |  | ✓ |  |  | ✓ |  |  |  |  |  |  |  |  |  |  | ✓ | ✓ |  |  |  |  |  |  | ✓ |  |  | 2008 | 2009 | 1 |
| Fogarty and Chapman [143] | 2012 | Australia | ✓ |  |  |  | ✓ |  |  | ✓ |  |  |  |  |  |  |  |  |  |  | ✓ |  |  |  |  |  |  |  | ✓ |  |  | 2005 | 2010 | 5 |
| Fogarty and Chapman [142] | 2012 | Australia | ✓ |  |  |  | ✓ |  |  | ✓ |  |  |  |  |  |  |  |  |  |  |  | ✓ |  |  |  |  |  |  | ✓ |  |  | 2008 | 2010 | 2 |
| Foley, O'Mahony, Lehane, Cil and Corrigan [144] | 2015 | Ireland |  |  |  | ✓ | ✓ |  |  |  |  |  |  |  | ✓ |  |  |  |  |  |  |  |  |  |  |  | ✓ |  | ✓ |  |  | 2007 | 2013 | 5 |
| Folta, Goldberg, Economos, Bell and Meltzer [145] | 2006 | US |  | ✓ |  |  | ✓ |  |  |  |  | ✓ | ✓ |  |  |  |  |  |  |  | ✓ |  |  |  |  |  |  |  | ✓ |  |  | 2003 | 2003 | <1 |
| Foss and Southwell [146] | 2006 | US |  | ✓ |  |  | ✓ |  |  |  |  | ✓ |  |  |  |  |  |  | ✓ |  |  |  | ✓ |  |  |  |  |  | ✓ |  |  | 1971 | 1999 | 28 |
| Fosu, Wicks, Warren and Wicks [147] | 2013 | US |  | ✓ |  |  | ✓ |  |  |  |  | ✓ |  |  |  |  |  |  |  |  | ✓ |  |  |  |  |  |  |  | ✓ |  |  | 2008 | 2008 | <1 |
| Franko, Rodgers, Lovering, Fernandes, Alfieri, Matsumoto, Accomando and Thompson-Brenner [148] | 2013 | US | ✓ |  |  |  | ✓ |  |  |  |  |  |  | ✓ |  |  |  |  |  |  |  |  | ✓ |  |  |  |  |  | ✓ |  |  | 1996 | 2011 | 15 |
| Frederick, Saguy, Sandhu and Mann [149] | 2016 | USA | ✓ |  |  |  |  | ✓ |  |  |  |  |  | ✓ |  |  |  |  |  |  |  |  |  |  |  |  |  | ✓ |  | ✓ |  | N/A | N/A | N/A |
| Freeman [150] | 2011 | Australia | ✓ |  |  | ✓ | ✓ |  |  |  | ✓ |  |  |  |  |  |  |  |  |  |  |  |  |  |  |  | ✓ |  | ✓ |  |  | 2010 | 2010 | <1 |
| Freeman, Chapman and Storey [151] | 2008 | Australia | ✓ |  |  |  | ✓ |  |  |  | ✓ |  |  |  |  |  |  |  |  |  |  | ✓ |  |  |  |  |  |  | ✓ |  |  | 1995 | 2007 | 12 |
| Frerichs, Andsager, Campo, Aquilino and Dyer [152] | 2006 | US | ✓ |  |  |  | ✓ |  |  |  |  | ✓ |  |  |  |  |  |  | ✓ |  |  |  | ✓ |  |  |  |  |  | ✓ |  |  | 1997 | 2003 | 6 |
| Friedman, Laditka, Laditka and Price [153] | 2011 | US | ✓ |  |  |  | ✓ |  |  |  |  | ✓ | ✓ |  |  |  |  |  | ✓ |  |  |  | ✓ |  |  |  |  |  | ✓ |  |  | 2006 | 2007 | 1 |
| Fu and Yip [154] | 2008 | Hong Kong | ✓ |  |  |  | ✓ |  |  |  |  |  |  |  |  |  |  |  | ✓ |  |  | ✓ |  |  |  |  |  |  | ✓ |  |  | 2004 | 2006 | 2 |
| Galcheva, Iotova and Stratev [155] | 2008 | Bulgaria |  | ✓ |  |  | ✓ |  |  |  |  | ✓ |  |  |  |  |  |  |  |  | ✓ |  |  |  |  |  |  |  | ✓ |  |  | 2007 | 2007 | <1 |
| Gantz and Wang [156] | 2009 | US | ✓ |  |  |  | ✓ |  |  |  |  |  |  |  | ✓ |  |  |  |  |  | ✓ |  |  |  |  |  |  |  | ✓ |  |  | 2004 | 2005 | 1 |
| Gase, Montes, Robles, Tyree and Kuo [157] | 2016 | US | ✓ |  |  |  | ✓ |  | ✓ |  |  | ✓ |  |  |  |  |  |  |  |  | ✓ |  |  | ✓ |  |  | ✓ |  | ✓ | ✓ |  | 2013 | 2014 | 1 |
| Gearhart, Craig and Steed [158] | 2012 | US | ✓ |  |  |  | ✓ |  |  |  |  | ✓ |  |  |  |  |  |  |  |  | ✓ |  |  |  |  |  |  |  | ✓ |  |  | 1995 | 2009 | 14 |
| Geyzen [159] | 2011 | Belgium | ✓ |  |  |  | ✓ |  |  |  |  | ✓ |  |  |  |  |  |  |  |  |  |  | ✓ |  |  |  |  |  | ✓ |  |  | 1945 | 1960 | 15 |
| Ghaznavi and Taylor [160] | 2015 | US |  |  |  | ✓ | ✓ |  |  |  |  |  |  | ✓ |  |  |  |  |  |  |  |  |  |  |  |  | ✓ |  | ✓ |  |  | N/A | N/A | N/A |
| Godbold Kean and Prividera [161] | 2007 | US |  | ✓ |  |  | ✓ |  |  | ✓ |  | ✓ |  |  |  |  |  |  |  |  |  |  | ✓ |  |  |  |  |  | ✓ |  |  | 2004 | 2004 | <1 |
| Gold, Cohen and Shumate [162] | 2008 | US |  | ✓ |  |  | ✓ |  |  |  | ✓ |  |  |  |  |  |  |  |  |  | ✓ |  |  |  |  |  |  |  | ✓ |  |  | 1999 | 2003 | 4 |
| Gollust and Lantz [163] | 2009 | US | ✓ |  |  |  | ✓ |  |  |  |  |  |  |  |  |  | ✓ |  |  | ✓ |  | ✓ |  |  |  |  |  |  | ✓ |  |  | 2005 | 2006 | 1 |
| Gollust, Lantz and Ubel [164] | 2009 | US | ✓ |  |  |  |  | ✓ |  |  |  |  |  |  |  |  | ✓ |  |  | ✓ |  |  |  |  |  |  |  | ✓ |  | ✓ |  | N/A | N/A | N/A |
| Gould, Kleinman, Lake, Forman and Midle [165] | 2014 | US | ✓ |  |  |  | ✓ |  |  |  |  |  |  |  |  |  |  |  | ✓ |  |  | ✓ |  |  |  |  |  |  | ✓ | ✓ |  | 1988 | 1996 | 8 |
| Goulden, Corker, Evans-Lacko, Rose, Thornicroft and Henderson [166] | 2011 | UK | ✓ |  |  |  | ✓ |  |  |  |  |  |  |  |  |  |  |  | ✓ |  |  | ✓ |  |  |  |  |  |  | ✓ |  |  | 1992 | 2008 | 16 |
| Graham, Fang, Moreno, Streiff, Villegas, Munoz, Tercyak, Mandelblatt and Vallone [167] | 2012 | US |  | ✓ |  |  | ✓ | ✓ | ✓ |  | ✓ |  |  |  |  |  |  |  |  |  |  |  |  |  |  |  | ✓ |  | ✓ |  |  | 2009 | 2009 | <1 |
| Grana and Ling [168] | 2014 | US |  | ✓ |  |  | ✓ |  |  |  | ✓ |  |  |  |  |  |  |  |  |  |  |  |  |  |  |  | ✓ |  | ✓ |  |  | 2011 | 2011 | <1 |
| Granner, Sharpe, Burroughs, Fields and Hallenbeck [169] | 2010 | US | ✓ |  |  |  | ✓ |  |  |  |  |  | ✓ |  |  |  |  |  |  |  |  | ✓ |  |  |  |  |  |  | ✓ |  |  | 2003 | 2005 | 2 |
| Greenberg and Wartenberg [170] | 1990 | US | ✓ |  |  |  | ✓ |  |  |  |  |  |  |  | ✓ |  |  |  | ✓ |  | ✓ |  |  |  |  |  |  |  | ✓ |  |  | 1978 | 1987 | 9 |
| Gruber, Thau, Hill, Fisher and Grube [171] | 2005 | US |  |  | ✓ |  | ✓ |  |  | ✓ | ✓ |  |  |  |  |  |  |  |  |  |  |  |  |  |  | ✓ |  |  | ✓ |  |  | 2001 | 2001 | <1 |
| Guenther, Froehlich, Milde, Heidecke and Ruhrmann [172] | 2015 | Germany | ✓ |  |  |  |  | ✓ |  |  |  |  |  |  | ✓ |  |  |  |  |  | ✓ |  |  |  |  |  |  |  |  | ✓ |  | N/A | N/A | N/A |
| Gugsa, Karmarkar, Cheyne and Yamey [173] | 2016 | Bangladesh, Rwanda, South Africa | ✓ |  |  |  | ✓ |  |  |  |  |  |  |  |  |  |  |  | ✓ |  |  | ✓ |  |  |  |  |  |  | ✓ |  |  | 2008 | 2013 | 5 |
| Gunderson, Clements and Benjamin Neelon [174] | 2014 | Honduras |  | ✓ |  |  | ✓ |  |  |  |  | ✓ |  |  |  |  |  |  |  |  | ✓ |  |  |  |  |  |  |  | ✓ |  |  | 2012 | 2012 | <1 |
| Guran, Turan, Akcay, Degirmenci, Avci, Asan, Erdil, Majid and Bereket [175] | 2010 | Turkey |  | ✓ |  |  | ✓ |  |  |  |  | ✓ |  |  |  |  |  |  |  |  | ✓ |  |  |  |  |  |  |  | ✓ |  |  | 2007 | 2007 | <1 |
| Haddock, Hoffman, Taylor, Schwab, Poston and Lando [176] | 2008 | US |  | ✓ |  |  | ✓ |  |  |  | ✓ |  |  |  |  |  |  |  |  |  |  |  | ✓ |  |  |  |  |  | ✓ |  |  | 2005 | 2005 | <1 |
| Haddock, Parker, Taylor, Poston, Lando and Talcott [177] | 2005 | US | ✓ | ✓ |  |  | ✓ |  |  | ✓ | ✓ |  | ✓ |  |  |  |  |  |  |  |  | ✓ | ✓ |  |  |  |  |  | ✓ |  |  | 2001 | 2004 | 3 |
| Hale, Pathipati, Zan and Jethwani [178] | 2014 | US |  |  |  | ✓ | ✓ |  |  |  |  |  |  |  |  |  |  |  | ✓ |  |  |  |  |  |  |  | ✓ |  | ✓ |  |  | 2007 | 2007 | <1 |
| Hamad, Pomeranz, Siddiqi and Basu [179] | 2015 | US | ✓ |  |  |  | ✓ |  |  |  |  |  |  | ✓ |  |  |  |  |  |  |  | ✓ |  |  |  |  |  |  | ✓ |  |  | 2011 | 2012 | 1 |
| Hampl, Wharton, Taylor, Winham, Block and Hall [180] | 2004 | US |  |  | ✓ |  | ✓ |  | ✓ |  |  | ✓ |  |  |  |  |  |  |  |  | ✓ |  |  |  |  |  |  |  |  | ✓ |  | 2003 | 2003 | <1 |
| Hanewinkel and Wiborg [181] | 2007 | Germany |  |  | ✓ |  | ✓ |  |  |  | ✓ |  |  |  |  |  |  |  |  |  | ✓ |  |  |  | ✓ |  |  |  | ✓ |  |  | 2005 | 2005 | <1 |
| Hanewinkel and Wiborg [182] | 2008 | Germany |  |  | ✓ |  | ✓ |  |  |  | ✓ |  |  |  |  |  |  |  |  |  | ✓ |  |  |  |  |  |  |  | ✓ |  |  | 1985 | 2004 | 19 |
| Harris, LoDolce, Dembek and Schwartz [185] | 2015 | US |  | ✓ |  |  | ✓ |  |  |  |  | ✓ |  |  |  |  |  |  |  |  | ✓ |  |  |  |  |  |  |  | ✓ |  |  | 2008 | 2011 | 3 |
| Harris, Moreland-Russell, Choucair, Mansour, Staub and Simmons [183] | 2014 | US |  |  |  | ✓ | ✓ |  |  |  | ✓ |  |  |  |  |  |  |  |  |  |  |  |  |  |  |  | ✓ |  | ✓ |  |  | 2014 | 2014 | <1 |
| Harris, Mueller, Snider and Haire-Joshu [184] | 2013 | US |  |  |  | ✓ | ✓ |  |  |  |  |  |  |  |  |  | ✓ |  |  |  |  |  |  |  |  |  | ✓ |  | ✓ |  |  | 2011 | 2012 | 1 |
| Hartman, Nierkens, Cremer, Verhoeff and Stronks [186] | 2015 | Netherlands | ✓ |  |  |  |  |  | ✓ |  |  | ✓ | ✓ | ✓ |  |  |  |  |  |  |  |  |  |  |  |  |  |  |  | ✓ |  | N/A | N/A | N/A |
| Harwood, Witson, Fan and Wagenaar [187] | 2005 | US | ✓ |  |  |  | ✓ |  |  | ✓ |  |  |  |  |  |  |  |  |  |  |  | ✓ |  |  |  |  |  |  | ✓ |  |  | 1994 | 2003 | 9 |
| Hawkins and Linvill [188] | 2010 | US | ✓ |  |  |  | ✓ |  |  |  |  |  |  | ✓ |  |  |  |  |  |  |  | ✓ |  |  |  |  |  |  | ✓ |  |  | 1996 | 2006 | 10 |
| Hayes, Ross, Gasher, Gutstein, Dunn and Hackett [189] | 2007 | Canada | ✓ |  |  |  | ✓ |  |  |  |  |  |  |  |  |  |  |  | ✓ | ✓ |  | ✓ |  |  |  |  |  |  | ✓ |  |  | 1993 | 2001 | 8 |
| He, Shen, Yin, Xu and Lan [190] | 2014 | China | ✓ |  |  |  | ✓ |  |  |  | ✓ |  |  |  |  |  |  |  |  |  |  | ✓ |  |  |  |  |  |  | ✓ |  |  | 2008 | 2011 | 3 |
| Hellman and Karlsson [191] | 2012 | Finland | ✓ |  |  |  | ✓ |  |  | ✓ |  |  |  |  |  |  |  |  |  |  |  | ✓ |  |  |  |  |  |  | ✓ |  |  | 2003 | 2004 | 1 |
| Hellyer and Haddock-Fraser [192] | 2011 | UK | ✓ |  |  |  | ✓ |  |  |  |  |  |  |  |  | ✓ | ✓ |  |  | ✓ |  | ✓ |  |  |  |  |  |  | ✓ |  |  | 2008 | 2008 | <1 |
| Henderson and Kelly [195] | 2005 | US |  | ✓ |  |  | ✓ |  |  |  |  | ✓ |  |  |  |  |  |  |  |  | ✓ |  |  |  |  |  |  |  | ✓ |  |  | 2003 | 2003 | <1 |
| Henderson, Coveney, Ward and Taylor [193] | 2009 | Australia | ✓ |  |  |  | ✓ |  |  |  |  |  |  | ✓ |  |  |  |  |  |  |  | ✓ |  |  |  |  |  |  | ✓ |  |  | 2006 | 2008 | 2 |
| Henderson, Kitzinger and Green [194] | 2000 | UK | ✓ |  |  |  | ✓ |  |  |  |  | ✓ |  |  |  |  |  |  | ✓ |  | ✓ | ✓ | ✓ |  |  |  |  |  | ✓ |  |  | 1999 | 1999 | <1 |
| Heneghan, Hazan, Halpern and Oliveria [196] | 2007 | US | ✓ |  |  |  | ✓ |  |  |  |  |  |  |  | ✓ |  |  |  |  |  |  | ✓ |  |  |  |  |  |  | ✓ |  |  | 1980 | 2004 | 24 |
| Heuer, McClure and Puhl [197] | 2011 | US | ✓ |  |  |  | ✓ |  |  |  |  |  |  | ✓ |  |  |  |  |  |  |  |  |  |  |  |  | ✓ |  | ✓ |  |  | 2009 | 2009 | <1 |
| Higgins, Naylor, Berry, O'Connor and McLean [198] | 2006 | US | ✓ |  |  |  | ✓ |  |  |  | ✓ | ✓ | ✓ | ✓ | ✓ | ✓ | ✓ | ✓ | ✓ | ✓ | ✓ | ✓ |  | ✓ |  |  | ✓ |  | ✓ |  |  | 1999 | 2003 | 4 |
| Hilbert and Ried [199] | 2009 | Germany | ✓ |  |  |  | ✓ |  |  |  |  |  |  | ✓ |  |  |  |  |  |  |  | ✓ |  |  |  |  |  |  | ✓ |  |  | 2006 | 2006 | <1 |
| Hill and Radimer [200] | 1996 | Australia |  | ✓ |  |  | ✓ |  |  |  |  | ✓ |  |  |  |  |  |  |  |  |  |  | ✓ |  |  |  |  |  | ✓ |  |  | 1992 | 1992 | 1 |
| Hilton and Hunt [201] | 2010 | UK | ✓ |  |  |  | ✓ |  |  |  |  |  |  |  | ✓ |  |  |  |  |  |  | ✓ |  |  |  |  |  |  | ✓ |  |  | 2008 | 2009 | 1 |
| Hilton, Patterson and Teyhan [202] | 2012 | UK | ✓ |  |  |  | ✓ |  |  |  |  |  |  | ✓ |  |  |  |  |  |  |  | ✓ |  |  |  |  |  |  | ✓ |  |  | 1996 | 2010 | 14 |
| Hilton, Wood, Bain, Patterson, Duffy and Semple [203] | 2014 | UK | ✓ |  |  |  | ✓ |  |  |  | ✓ |  |  |  |  |  |  |  |  |  |  | ✓ |  |  |  |  |  |  | ✓ |  |  | 2004 | 2014 | 10 |
| Hilton, Wood, Patterson and Katikireddi [204] | 2014 | UK | ✓ |  |  |  | ✓ |  |  | ✓ |  |  |  |  |  |  |  |  |  |  |  | ✓ |  |  |  |  |  |  | ✓ |  |  | 2001 | 2012 | 11 |
| Himes and Thompson [205] | 2007 | US |  |  | ✓ |  | ✓ |  |  |  |  |  |  | ✓ |  |  |  |  |  |  | ✓ |  |  |  | ✓ |  |  |  | ✓ |  |  | 1984 | 2004 | 20 |
| Hinnant, Oh, Caburnay and Kreuter [206] | 2011 | US | ✓ |  |  |  |  | ✓ |  |  |  |  |  |  | ✓ |  |  |  |  | ✓ |  |  |  |  |  |  |  | ✓ |  | ✓ | ✓ | N/A | N/A | N/A |
| Hoffman-Goetz, Shannon and Clarke [207] | 2003 | Canada | ✓ |  |  |  | ✓ |  |  |  |  |  |  |  | ✓ | ✓ | ✓ |  |  |  |  | ✓ |  |  |  |  |  |  | ✓ |  |  | 1996 | 2010 | 14 |
| Hoffner and Ye [208] | 2009 | US | ✓ |  |  |  |  | ✓ |  |  |  |  |  |  | ✓ |  |  |  |  |  |  |  |  |  |  |  |  | ✓ |  | ✓ |  | N/A | N/A | N/A |
| Holland, Blood, Thomas and Lewis [209] | 2015 | Australia | ✓ |  |  |  |  |  | ✓ |  |  |  |  | ✓ |  |  |  |  |  |  |  |  |  |  |  |  |  |  |  | ✓ |  | N/A | N/A | N/A |
| Holland, Blood, Thomas, Lewis, Komesaroff and Castle [210] | 2011 | Australia | ✓ |  |  |  | ✓ |  |  |  |  |  |  | ✓ |  |  |  |  |  |  | ✓ | ✓ |  |  |  |  |  |  | ✓ |  |  | 2008 | 2008 | <1 |
| Holmes [212] | 2009 | Canada | ✓ |  |  |  | ✓ |  |  |  |  |  |  | ✓ |  |  |  |  |  |  |  | ✓ |  |  |  |  |  |  | ✓ |  |  | 1996 | 2006 | 10 |
| Holmes-Rovner and Charles [211] | 2003 | UK | ✓ |  |  |  | ✓ |  |  |  |  |  |  |  | ✓ |  |  |  |  |  |  | ✓ |  |  |  |  |  | ✓ | ✓ |  |  | 2000 | 2000 | <1 |
| Holton, Lee and Coleman [213] | 2014 | US | ✓ |  |  |  | ✓ | ✓ |  |  |  |  |  |  | ✓ |  |  |  | ✓ |  |  |  |  |  |  |  | ✓ |  | ✓ | ✓ |  | 2010 | 2011 | 1 |
| Huang, Mehta and Wong [214] | 2012 | Singapore |  | ✓ |  |  | ✓ |  |  |  |  | ✓ |  |  |  |  |  |  |  |  | ✓ |  |  |  |  |  |  |  | ✓ |  |  | 2007 | 2007 | <1 |
| Hubbell and Dearing [215] | 2003 | US | ✓ |  |  |  | ✓ |  |  |  |  |  |  |  |  |  |  |  | ✓ |  |  | ✓ |  |  |  |  |  |  | ✓ |  |  | 1993 | 1997 | 4 |
| Hurley, Kosenko and Brashers [216] | 2011 | US | ✓ |  |  |  | ✓ |  |  |  |  |  |  |  | ✓ |  |  |  |  |  |  |  |  |  |  |  | ✓ |  | ✓ |  |  | 2008 | 2008 | <! |
| Hurley, Riles and Sangalang [217] | 2014 | US | ✓ |  |  |  | ✓ |  |  |  |  |  |  |  | ✓ |  |  |  |  |  |  |  |  |  |  |  | ✓ |  | ✓ |  |  | 2008 | 2008 | <1 |
| Jahns, Payne, Whigham, Johnson, Scheett, Hoverson and Kranz [218] | 2014 | US | ✓ |  |  |  | ✓ |  |  |  |  |  |  |  | ✓ |  |  |  |  |  |  |  |  |  |  |  | ✓ |  | ✓ |  |  | 2008 | 2008 | <1 |
| Jahns, Payne, Whigham, Johnson, Scheett, Hoverson and Kranz [218] | 2014 | US |  | ✓ |  |  | ✓ |  |  |  |  | ✓ |  |  |  |  |  |  |  |  |  | ✓ |  |  |  |  |  | ✓ | ✓ |  |  | 2009 | 2009 | <1 |
| Jarlenski and Barry [219] | 2013 | US | ✓ |  |  |  | ✓ |  |  |  |  | ✓ |  |  |  |  |  |  |  |  | ✓ | ✓ |  |  |  |  |  |  | ✓ |  |  | 1998 | 2008 | 10 |
| Jawad, Bakir, Ali, Jawad and Akl [220] | 2015 | UK | ✓ |  |  |  | ✓ |  |  |  | ✓ |  |  |  |  |  |  |  |  |  |  |  |  |  |  |  | ✓ |  | ✓ |  |  | 2011 | 2012 | 1 |
| Jenkin, Signal and Thomson [221] | 2011 | New Zealand |  |  |  |  | ✓ |  |  | ✓ | ✓ | ✓ |  | ✓ |  |  |  |  |  |  |  |  |  |  |  |  |  | ✓ | ✓ |  |  | N/A | N/A | N/A |
| Jenssen, Klein, Salazar, Daluga and DiClemente [222] | 2009 | US |  | ✓ |  | ✓ | ✓ |  |  |  | ✓ |  |  |  |  |  |  |  |  |  |  |  |  |  |  |  | ✓ |  | ✓ | ✓ |  | 2004 | 2004 | <1 |
| Jeong, Gilmore, Bleakley and Jordan [223] | 2014 | US | ✓ |  |  |  | ✓ |  |  |  |  |  |  | ✓ |  |  |  |  |  |  | ✓ | ✓ |  | ✓ |  |  | ✓ |  | ✓ |  |  | 2010 | 2011 | 1 |
| Jernigan and Wright [224] | 1996 | US |  |  |  |  | ✓ |  |  | ✓ | ✓ |  |  |  |  |  |  |  |  |  |  |  |  |  |  |  |  | ✓ | ✓ |  |  | N/A | N/A | N/A |
| Johnson and Johnson [225] | 1993 | UK |  |  | ✓ |  | ✓ |  |  |  |  |  |  |  |  |  |  |  | ✓ |  | ✓ |  |  |  |  |  |  |  | ✓ |  |  | 1988 | 1989 | 1 |
| Jones, Merrick and Beasley [227] | 2016 | Australia | ✓ |  |  |  | ✓ |  |  |  |  |  |  |  |  |  |  |  | ✓ |  | ✓ | ✓ | ✓ |  |  |  |  |  | ✓ |  |  | 2012 | 2012 | <1 |
| Jones-Webb, Baranowski, Fan, Finnegan and Wagenaar [226] | 1997 | US | ✓ |  |  |  | ✓ |  |  | ✓ |  |  |  |  |  |  |  |  |  |  |  | ✓ |  |  |  |  |  |  | ✓ |  |  | 1993 | 1995 | 2 |
| Kaczynski, Havitz and McCarville [228] | 2005 | Canada | ✓ |  |  |  |  | ✓ |  |  |  |  | ✓ |  |  |  |  |  |  |  |  |  |  |  |  |  |  | ✓ |  | ✓ |  | N/A | N/A | N/A |
| Kalin and Fung [229] | 2013 | US | ✓ |  |  |  | ✓ |  |  |  |  |  |  | ✓ |  |  |  |  |  |  | ✓ |  |  |  |  |  |  |  | ✓ |  |  | 2007 | 2009 | 2 |
| Kang and Lin [230] | 2015 | Korea |  | ✓ |  |  |  | ✓ |  |  | ✓ |  |  |  |  |  |  |  |  |  |  |  |  |  |  |  |  | ✓ |  | ✓ |  | N/A | N/A | N/A |
| Kang, Gearhart and Bae [231] | 2010 | US | ✓ |  |  |  | ✓ |  |  |  |  |  |  |  |  |  |  |  | ✓ |  | ✓ |  |  |  |  |  |  |  | ✓ |  |  | 1984 | 2008 | 24 |
| Karupaiah, Chinna, Mee, Mei and Noor [232] | 2008 | Malaysia |  | ✓ |  |  | ✓ |  |  |  |  | ✓ |  |  |  |  |  |  |  |  | ✓ |  |  |  |  |  |  |  | ✓ |  |  | 2006 | 2006 | <1 |
| Kees [233] | 2011 | US |  | ✓ |  |  |  | ✓ |  |  |  | ✓ |  | ✓ |  |  |  |  |  |  |  |  |  |  |  |  |  | ✓ |  | ✓ |  | N/A | N/A | N/A |
| Keller and Schulz [234] | 2011 | Switzerland |  | ✓ |  |  | ✓ |  |  |  |  | ✓ |  |  |  |  |  |  |  |  | ✓ |  |  |  |  |  |  |  | ✓ |  |  | 2006 | 2006 | <1 |
| Kelly and Chapman [235] | 2007 | Australia | ✓ |  |  |  | ✓ |  |  |  |  | ✓ |  |  |  |  |  |  |  |  |  |  | ✓ |  |  |  |  |  | ✓ |  |  | 2006 | 2006 | <1 |
| Kennedy and Bero [236] | 1999 | US | ✓ |  |  |  | ✓ |  |  |  | ✓ |  |  |  |  |  |  |  |  |  |  | ✓ | ✓ |  |  |  |  |  | ✓ |  |  | 1981 | 1994 | 13 |
| Kent, Dubois and Wanless [238] | 2011 | Canada |  | ✓ |  |  | ✓ |  | ✓ |  |  | ✓ |  |  |  |  |  |  |  |  | ✓ |  |  |  |  |  |  |  | ✓ |  | ✓ | 2009 | 2009 | <1 |
| Kent, Dubois, Kent and Wanless [237] | 2013 | Canada |  | ✓ |  |  | ✓ |  |  |  |  | ✓ |  |  |  |  |  |  |  |  |  |  |  |  |  |  | ✓ |  | ✓ |  |  | 2009 | 2009 | <1 |
| Kerr [239] | 2014 | US |  |  | ✓ |  | ✓ |  |  | ✓ |  |  |  |  |  |  |  |  |  |  | ✓ |  |  |  |  |  |  |  | ✓ |  |  | 2003 | 2008 | 5 |
| Kesten, Cohn and Ogilvie [240] | 2014 | UK | ✓ |  |  | ✓ | ✓ |  |  |  |  |  | ✓ |  |  |  |  |  | ✓ |  |  | ✓ |  |  |  |  | ✓ |  | ✓ |  |  | 2004 | 2012 | 8 |
| Kim and Lennon [244] | 2006 | Korea |  | ✓ |  |  | ✓ |  |  |  |  | ✓ |  |  |  |  |  |  |  |  |  |  | ✓ |  |  |  |  |  | ✓ |  |  | 2001 | 2001 | <1 |
| Kim and Niederdeppe [246] | 2014 | US |  |  |  |  |  | ✓ |  |  | ✓ |  |  |  |  |  |  |  |  |  | ✓ |  |  |  |  |  |  |  |  | ✓ |  | N/A | N/A | N/A |
| Kim and Willis [245] | 2007 | US | ✓ |  |  |  | ✓ |  |  |  |  |  |  | ✓ |  |  |  |  |  |  | ✓ | ✓ |  |  |  |  |  |  | ✓ |  |  | 1995 | 2004 | 9 |
| Kim, Hou, Han and Himelboim [242] | 2016 | US |  |  |  | ✓ | ✓ |  |  |  |  |  |  |  | ✓ |  |  |  |  |  |  |  |  |  |  |  | ✓ |  | ✓ |  |  | 2011 | 2011 | <1 |
| Kim, Kumanyika, Shive, Igweatu and Kim [241] | 2010 | US | ✓ |  |  |  | ✓ |  |  |  |  |  |  |  |  |  |  |  | ✓ | ✓ |  | ✓ |  |  |  |  |  |  | ✓ |  |  | 1996 | 2005 | 9 |
| Kim, Paek and Lynn [243] | 2010 | US |  |  |  | ✓ | ✓ |  |  |  | ✓ |  |  |  |  |  |  |  |  |  |  |  |  |  | ✓ |  | ✓ |  | ✓ |  |  | 2006 | 2007 | 1 |
| Klos, Greenleaf, Paly, Kessler, Shoemaker and Suchla [247] | 2015 | US |  |  | ✓ |  | ✓ |  |  |  |  | ✓ | ✓ | ✓ |  |  |  |  |  |  | ✓ |  |  |  |  |  |  |  | ✓ |  |  | 2010 | 2012 | 2 |
| Knox, Biddle, Esliger, Piggin and Sherar [248] | 2015 | US, Canada, Australia |  | ✓ |  |  | ✓ |  |  |  |  |  | ✓ |  |  |  |  |  |  |  |  |  |  |  |  |  |  | ✓ | ✓ |  |  | 2014 | 2014 | <1 |
| Konfortion, Jack and Davies [249] | 2014 | UK | ✓ |  |  |  | ✓ |  |  |  |  |  |  |  | ✓ |  |  |  |  |  |  | ✓ |  |  |  |  |  |  | ✓ |  |  | 2011 | 2012 | 1 |
| Kostygina, Hahn and Rayens [250] | 2014 | US |  | ✓ |  |  | ✓ |  | ✓ |  | ✓ |  |  |  |  |  |  |  |  |  |  |  |  |  |  |  |  |  | ✓ |  |  | N/A | N/A | N/A |
| Krauss, Sowles, Moreno, Zewdie, Grucza, Bierut and Cavazos-Rehg [251] | 2015 | US |  |  |  | ✓ | ✓ |  |  |  | ✓ |  |  |  |  |  |  |  |  |  |  |  |  |  |  |  | ✓ |  | ✓ |  |  | 2014 | 2014 | <1 |
| Krishen and Bui [252] | 2015 | US |  | ✓ |  |  |  | ✓ |  |  |  | ✓ |  |  |  |  |  |  |  |  |  |  |  |  |  |  |  | ✓ |  | ✓ |  | N/A | N/A | N/A |
| Kromm, Smith and Singer [253] | 2007 | US | ✓ |  |  |  | ✓ |  |  |  |  |  |  |  | ✓ |  |  |  |  |  |  | ✓ |  |  |  |  |  |  | ✓ |  |  | 2005 | 2005 | <1 |
| Kuiper, Frantz, Cotant, Babb, Jordan and Phelan [254] | 2013 | US | ✓ |  |  |  | ✓ |  |  |  | ✓ |  |  |  |  |  |  |  |  |  |  | ✓ |  |  |  |  | ✓ |  | ✓ |  |  | 2010 | 2010 | <1 |
| Kunkel, Castonguay and Filer [255] | 2015 | US |  | ✓ |  |  | ✓ |  |  |  |  | ✓ |  |  |  |  |  |  |  |  | ✓ |  |  |  |  |  |  |  | ✓ |  |  | 2014 | 2014 | <1 |
| Kurko, Linden, Kolstela, Pietila and Airaksinen [256] | 2015 | Finland |  |  |  | ✓ | ✓ |  |  |  | ✓ |  |  |  |  |  |  |  |  |  |  |  |  |  |  |  | ✓ |  | ✓ |  |  | 2007 | 2012 | 5 |
| Kye, Kwon, Kim, Shim, Kim, Cho, Jung and Park [257] | 2015 | Korea | ✓ |  |  |  | ✓ |  |  |  |  |  |  |  | ✓ |  |  |  |  |  | ✓ | ✓ |  |  |  |  |  |  | ✓ |  |  | 2008 | 2012 | 5 |
| Lando, Michaud, Poston, Jahnke, Williams and Haddock [258] | 2015 | US | ✓ |  |  |  | ✓ |  | ✓ |  | ✓ |  |  |  |  |  |  |  |  |  |  | ✓ |  |  |  |  |  |  | ✓ |  |  | 2010 | 2011 | 1 |
| Lapinski [259] | 2006 | US | ✓ |  |  |  | ✓ |  |  |  |  | ✓ |  | ✓ |  |  |  |  | ✓ |  |  |  |  |  |  |  | ✓ |  | ✓ |  |  | 2003 | 2003 | <1 |
| Larson, Long, Slater, Bettinghaus and Read [260] | 2009 | US | ✓ |  |  |  | ✓ |  |  |  |  |  |  |  | ✓ |  |  |  |  |  | ✓ | ✓ |  |  |  |  |  |  | ✓ |  |  | 2002 | 2003 | 1 |
| Lascu, Manrai, Manrai and Amissah [261] | 2013 | US, France, Spain |  | ✓ |  |  | ✓ |  |  |  |  | ✓ |  |  |  |  |  |  |  |  |  |  |  |  |  |  | ✓ |  | ✓ |  |  | N/A | N/A | N/A |
| Lavack [262] | 1999 | US |  | ✓ |  |  | ✓ |  |  | ✓ |  |  |  |  |  |  |  |  |  |  | ✓ |  |  |  |  |  |  |  | ✓ |  |  | 1980 | 1994 | 14 |
| Lawhon and Herrick [263] | 2013 | South Africa | ✓ |  |  |  | ✓ |  |  | ✓ |  |  |  |  |  |  |  |  |  |  |  | ✓ |  |  |  |  |  |  | ✓ |  |  | 2007 | 2011 | 4 |
| Lee and Cheng [270] | 2010 | US |  | ✓ |  |  | ✓ |  |  |  | ✓ |  |  |  |  |  |  |  |  |  | ✓ |  |  |  |  |  |  |  | ✓ |  |  | 2007 | 2007 | <1 |
| Lee and Gispanski [269] | 2016 | US |  |  | ✓ |  | ✓ |  |  |  |  | ✓ |  |  |  |  |  |  |  |  | ✓ |  |  |  |  |  |  |  | ✓ |  |  | 2009 | 2010 | 1 |
| Lee and Len-Rios [266] | 2014 | US | ✓ |  |  |  | ✓ |  |  |  |  |  |  | ✓ |  |  |  |  |  |  |  | ✓ |  |  |  |  |  |  | ✓ |  |  | 2004 | 2007 | 3 |
| Lee, Agnew-Brune, Clapp and Blosnich [267] | 2014 | US |  |  | ✓ |  | ✓ |  |  |  | ✓ |  |  |  |  |  |  |  |  |  |  |  |  |  | ✓ |  |  |  | ✓ |  |  | 2000 | 2011 | 11 |
| Lee, Choi, Quilliam and Cole [268] | 2009 | US |  | ✓ |  |  | ✓ |  |  |  |  | ✓ |  |  |  |  |  |  |  |  |  |  |  |  |  |  | ✓ |  | ✓ |  |  | 2006 | 2006 | <1 |
| Lee, Lee, Park, Willis and Cameron [265] | 2013 | US | ✓ |  |  |  | ✓ |  |  |  |  |  |  |  |  |  |  |  | ✓ |  | ✓ |  |  |  |  |  |  |  | ✓ |  |  | N/A | N/A | N/A |
| Lee, Long, Slater and Song [264] | 2014 | US |  |  |  | ✓ | ✓ |  |  |  |  |  |  |  |  |  |  |  | ✓ |  |  |  |  |  |  |  | ✓ |  | ✓ |  |  | 2012 | 2012 | <1 |
| Lee, Long, Slater and Song [264] | 2014 | US | ✓ |  |  |  | ✓ |  |  |  |  |  |  |  | ✓ |  |  |  |  |  | ✓ |  |  |  |  |  |  |  | ✓ |  |  | 2002 | 2003 | 1 |
| Lemal, Custers and Bulck [271] | 2010 | Belgium |  |  | ✓ |  | ✓ |  |  |  |  |  |  |  |  |  |  |  | ✓ |  | ✓ |  |  |  |  |  |  |  | ✓ |  |  | 2007 | 2007 | <1 |
| Lemmens, Vaeth and Greenfield [272] | 1999 | US | ✓ |  |  |  | ✓ |  |  | ✓ |  |  |  |  |  |  |  |  |  |  |  | ✓ |  |  |  |  |  |  | ✓ |  |  | 1985 | 1991 | 6 |
| Leshner and Cheng [274] | 2009 | US |  | ✓ |  |  |  | ✓ |  |  | ✓ |  |  |  |  |  |  |  |  |  | ✓ |  |  |  |  |  |  |  |  | ✓ |  | N/A | N/A | N/A |
| Leshner, Bolls and Wise [273] | 2011 | US |  | ✓ |  |  |  | ✓ |  |  | ✓ |  |  |  |  |  |  |  |  |  |  |  |  |  |  |  |  | ✓ |  | ✓ |  | N/A | N/A | N/A |
| Lewis and Hill [275] | 1998 | UK |  | ✓ |  |  | ✓ | ✓ |  |  |  | ✓ |  | ✓ |  |  |  |  |  |  | ✓ |  |  |  |  |  |  |  | ✓ | ✓ |  | 1996 | 1996 | <1 |
| Lima and Siegel [276] | 1999 | US | ✓ |  |  |  | ✓ |  |  |  | ✓ |  |  |  |  |  |  |  |  |  |  | ✓ |  |  |  |  |  |  | ✓ |  |  | 1997 | 1998 | 1 |
| Lindsay, Thomas, Lewis, Westberg, Moodie and Jones [277] | 2013 | Australia |  | ✓ |  |  | ✓ |  |  |  |  | ✓ |  |  |  |  |  |  |  |  | ✓ |  |  |  |  |  |  |  | ✓ |  |  | 2012 | 2012 | <1 |
| Lingas [278] | 2013 | US | ✓ |  |  |  | ✓ |  |  |  |  |  |  |  |  |  |  |  | ✓ |  |  | ✓ |  |  |  |  |  |  | ✓ |  |  | 2001 | 2001 | <1 |
| Liu, Liu, Xiao, Cai and Xu [279] | 2010 | China | ✓ |  |  |  | ✓ |  |  |  |  |  |  |  | ✓ |  |  |  |  |  |  | ✓ |  |  |  |  |  |  | ✓ |  |  | 2000 | 2007 | 7 |
| LoDolce, Harris and Schwartz [280] | 2013 | US |  | ✓ |  |  | ✓ |  |  |  |  | ✓ |  |  |  |  |  |  |  |  | ✓ |  |  |  |  |  |  |  | ✓ | ✓ |  | 2008 | 2009 | 1 |
| Long, Slater and Lysengen [281] | 2006 | US | ✓ |  |  |  | ✓ |  |  |  | ✓ |  |  |  |  |  |  |  |  |  | ✓ | ✓ | ✓ |  |  |  |  |  | ✓ |  |  | 2002 | 2003 | 1 |
| Love, Sterns, Spreen and Wysocki [282] | 2006 | US | ✓ |  |  |  | ✓ |  | ✓ |  |  | ✓ |  |  |  |  |  |  |  |  |  | ✓ |  |  |  |  |  |  | ✓ |  |  | 1995 | 2004 | 9 |
| Ludwick, Rushing and Biordi [283] | 1994 | US | ✓ |  |  |  | ✓ |  |  |  |  |  |  |  | ✓ |  |  |  |  |  |  |  | ✓ |  |  |  |  |  | ✓ |  |  | 1986 | 1991 | 5 |
| Ma, Fleisher, Gonzalez and Edwards [284] | 2004 | US | ✓ | ✓ |  |  | ✓ |  |  |  |  |  |  |  | ✓ |  |  |  |  |  |  | ✓ |  | ✓ |  |  |  |  | ✓ |  |  | 2001 | 2002 | 2 |
| MacKenzie, Chapman and Holding [285] | 2011 | Australia | ✓ |  |  |  | ✓ |  |  |  |  |  |  |  | ✓ |  |  |  |  |  | ✓ |  |  |  |  |  |  |  | ✓ |  |  | 2005 | 2009 | 4 |
| MacKenzie, Chapman, Holding and McGeechan [286] | 2007 | Australia | ✓ |  |  |  | ✓ |  |  |  |  |  |  |  | ✓ |  |  |  |  |  | ✓ | ✓ |  |  |  |  |  |  | ✓ |  |  | 2003 | 2006 | 3 |
| MacKenzie, Chapman, Holding and Stiven [287] | 2010 | Australia | ✓ |  |  |  | ✓ |  |  |  |  |  |  |  | ✓ |  |  |  |  |  | ✓ |  |  |  |  |  |  |  | ✓ |  |  | 2005 | 2007 | 2 |
| Mackenzie, Chapman, Johnson, McGeechan and Holding [288] | 2008 | Australia | ✓ |  |  |  | ✓ |  |  |  |  |  |  |  | ✓ |  |  |  |  |  | ✓ |  |  |  |  |  |  |  | ✓ |  |  | 2005 | 2008 | 3 |
| MacKenzie, Imison, Chapman and Holding [289] | 2008 | Australia | ✓ |  |  |  | ✓ |  |  |  |  |  |  |  | ✓ |  |  |  |  |  | ✓ | ✓ |  |  |  |  |  |  | ✓ |  |  | 2007 | 2008 | 1 |
| Mackenzie, Johnson, Chapman and Holding [290] | 2009 | Australia | ✓ |  |  |  | ✓ |  |  |  | ✓ |  |  |  |  |  |  |  |  |  | ✓ |  |  |  |  |  |  |  | ✓ |  |  | 2005 | 2007 | 2 |
| MacLean, Sweeting, Walker, Patterson, Raisanen and Hunt [291] | 2015 | UK | ✓ |  |  |  | ✓ |  |  |  |  | ✓ |  | ✓ |  |  |  |  | ✓ |  |  | ✓ |  |  |  |  |  |  | ✓ |  |  | 2002 | 2012 | 10 |
| Magzamen, Charlesworth and Glantz [292] | 2001 | US | ✓ |  |  |  | ✓ |  |  |  | ✓ |  |  |  |  |  |  |  |  |  |  | ✓ |  |  |  |  |  |  | ✓ |  |  | 1997 | 1998 | 1 |
| Maher, Wilson and Signal [293] | 2005 | New Zealand |  | ✓ |  |  | ✓ |  |  |  |  | ✓ |  |  |  |  |  |  |  |  |  |  |  |  |  |  |  | ✓ | ✓ |  |  | 2004 | 2005 | 1 |
| Mainland, Shaw and Prier [294] | 2015 | Canada | ✓ | ✓ |  |  | ✓ |  |  |  |  |  |  | ✓ |  |  |  |  |  |  |  |  | ✓ |  |  |  |  |  | ✓ |  |  | 2009 | 2009 | <1 |
| Major [295] | 2009 | US | ✓ |  |  |  |  | ✓ |  |  |  |  |  | ✓ | ✓ |  |  |  |  |  |  |  |  |  |  |  |  | ✓ |  | ✓ |  | N/A | N/A | N/A |
| Malone, Wenger and Bero [296] | 2002 | US | ✓ |  |  |  | ✓ |  | ✓ |  | ✓ |  |  |  |  |  |  |  |  |  |  | ✓ |  |  |  |  |  |  | ✓ |  |  | 1995 | 1999 | 4 |
| Manganello, Clegg Smith, Sudakow and Summers [297] | 2013 | US |  | ✓ |  |  | ✓ |  |  |  |  | ✓ |  |  |  |  |  |  |  |  |  |  | ✓ |  |  |  |  |  | ✓ |  |  | 2008 | 2008 | <1 |
| Mannien, van den Brandhof, McIntyre and Hiller [298] | 2002 | Australia | ✓ |  |  |  | ✓ |  |  |  |  | ✓ |  |  |  |  |  |  | ✓ |  |  | ✓ |  |  |  |  |  |  | ✓ |  |  | 1996 | 1999 | 3 |
| Martin-Biggers, Yorkin, Aljallad, Ciecierski, Akhabue, et al. [299] | 2013 | US |  | ✓ |  |  | ✓ |  |  |  |  | ✓ |  |  |  |  |  |  |  |  |  | ✓ |  |  |  |  |  | ✓ | ✓ |  |  | 2011 | 2011 | <1 |
| Martinez, Johnston-Robledo, Ulsh and Chrisler [300] | 2000 | US | ✓ |  |  |  | ✓ |  |  |  |  |  |  |  |  |  |  |  | ✓ |  |  |  | ✓ |  |  |  |  |  | ✓ |  |  | 1980 | 1998 | 18 |
| Mastin and Campo [301] | 2006 | US | ✓ | ✓ |  |  | ✓ |  |  |  |  |  |  | ✓ |  |  |  |  |  |  |  |  | ✓ |  |  |  |  |  | ✓ |  |  | 1984 | 2003 | 19 |
| McCauley, Blake, Meissner and Viswanath [302] | 2013 | US | ✓ |  |  |  |  |  | ✓ |  |  |  |  |  |  |  |  |  | ✓ |  |  |  |  |  |  |  |  |  |  |  | ✓ | N/A | N/A | N/A |
| McCaw, McGlade and McElnay [303] | 2014 | US & UK | ✓ |  |  |  | ✓ |  |  |  |  |  |  |  |  |  |  |  | ✓ |  |  | ✓ |  |  |  |  |  |  | ✓ |  |  | 2003 | 2012 | 9 |
| McDaniel, Offen, Yerger, Forsyth and Malone [304] | 2015 | US | ✓ |  |  |  | ✓ |  |  |  | ✓ |  |  |  |  |  |  |  |  |  |  | ✓ | ✓ |  |  |  | ✓ |  | ✓ |  |  | 1995 | 2011 | 16 |
| McGee and Ketchel [306] | 2006 | New Zealand |  |  | ✓ |  | ✓ |  |  |  | ✓ |  |  |  |  |  |  |  |  |  | ✓ |  |  |  |  |  |  |  | ✓ |  |  | 2004 | 2004 | <1 |
| McGee, Bang and Marsh [305] | 2014 | New Zealand | ✓ |  |  |  | ✓ |  |  |  | ✓ |  |  |  |  |  |  |  |  |  |  | ✓ |  |  |  |  |  |  | ✓ |  |  | 2011 | 2012 | 1 |
| McGee, Ketchel and Reeder [307] | 2007 | New Zealand |  | ✓ | ✓ |  | ✓ |  |  | ✓ |  |  |  |  |  |  |  |  |  |  | ✓ |  |  |  |  |  |  |  | ✓ |  |  | 2004 | 2004 | <1 |
| McHiza, Temple, Steyn, Abrahams and Clayford [308] | 2013 | South Africa |  | ✓ |  |  | ✓ |  |  |  |  | ✓ |  |  |  |  |  |  |  |  | ✓ |  |  |  |  |  |  |  | ✓ |  |  | 2011 | 2011 | <1 |
| McIntosh [309] | 2000 | US | ✓ |  |  |  | ✓ |  |  |  |  | ✓ |  |  |  |  |  |  |  |  |  |  | ✓ |  |  |  |  |  | ✓ |  |  | 1940 | 1999 | 59 |
| McLeod, Wakefield, Chapman, Smith and Durkin [310] | 2009 | Australia | ✓ |  |  |  | ✓ |  |  |  | ✓ |  |  |  |  |  |  |  |  |  |  | ✓ |  |  |  |  |  |  | ✓ |  |  | 1995 | 2005 | 10 |
| McWhirter and Hoffman-Goetz [311] | 2015 | North America | ✓ |  |  |  | ✓ |  |  |  |  |  |  |  | ✓ |  |  |  |  |  |  |  | ✓ |  |  |  |  |  | ✓ |  |  | 2001 | 2012 | 11 |
| McWhirter and Hoffman-Goetz [312] | 2015 | US, Canada | ✓ |  |  |  | ✓ |  |  |  |  |  |  |  | ✓ |  |  |  |  |  |  |  | ✓ |  |  |  |  |  | ✓ |  |  | 2007 | 2012 | 6 |
| McWhirter and Hoffman-Goetz [313] | 2015 | Canada | ✓ |  |  |  | ✓ |  |  |  |  |  |  |  | ✓ |  |  |  |  |  |  |  | ✓ |  |  |  |  |  | ✓ |  |  | 2000 | 2012 | 13 |
| McWhirter and Hoffman-Goetz [314] | 2016 | US | ✓ |  |  |  | ✓ |  |  |  |  |  |  |  | ✓ |  |  |  |  |  |  |  | ✓ |  |  |  |  |  | ✓ |  |  | 2000 | 2012 | 13 |
| McWhirter, Hoffman-Goetz and Clarke [315] | 2012 | Canada | ✓ |  |  |  | ✓ |  |  |  |  |  |  |  | ✓ |  |  |  |  |  |  |  | ✓ |  |  |  |  |  | ✓ |  |  | 2005 | 2010 | 5 |
| Mejia, Dorfman, Cheyne, Nixon, Friedman, Gottlieb and Daynard [316] | 2014 | US | ✓ |  |  |  | ✓ |  |  |  | ✓ |  |  |  |  |  |  |  |  |  |  | ✓ |  |  |  |  |  |  | ✓ |  |  | 1966 | 1991 | 25 |
| Mekemson, Glik, Titus, Myerson, Shaivitz, Ang and Mitchell [317] | 2004 | US |  |  | ✓ |  | ✓ |  |  |  | ✓ |  |  |  |  |  |  |  |  |  |  |  |  |  | ✓ |  |  |  | ✓ |  |  | 1991 | 2000 | 9 |
| Menashe and Siegel [318] | 1998 | US | ✓ |  |  |  | ✓ |  |  |  | ✓ |  |  |  |  |  |  |  |  |  |  | ✓ |  |  |  |  |  |  | ✓ |  |  | 1985 | 1996 | 11 |
| Mercurio and Eliott [319] | 2011 | Australia | ✓ |  |  |  | ✓ |  |  |  |  |  |  |  | ✓ |  |  |  |  |  |  | ✓ |  |  |  |  |  |  | ✓ |  |  | 1998 | 2007 | 10 |
| Mocarski and Bissell [320] | 2016 | US |  |  | ✓ |  | ✓ |  |  |  |  |  | ✓ | ✓ |  |  |  |  |  |  | ✓ |  |  |  |  |  |  |  | ✓ |  |  | 2004 | 2012 | 8 |
| Moodie, Angus and Ford [321] | 2014 | US |  | ✓ |  |  | ✓ |  |  |  | ✓ |  |  |  |  |  |  |  |  |  |  |  |  |  |  |  |  | ✓ | ✓ |  |  | 2004 | 2011 | 7 |
| Moore and Rideout [322] | 2007 | US |  | ✓ |  |  | ✓ |  |  |  | ✓ |  |  |  |  |  |  |  |  |  |  |  | ✓ |  |  |  | ✓ |  | ✓ |  |  | 2005 | 2005 | <1 |
| Moreno, Briner, Williams, Brockman, Walker and Christakis [323] | 2010 | US |  |  |  | ✓ | ✓ |  |  | ✓ |  |  |  |  |  |  |  |  |  |  |  |  |  |  |  |  | ✓ |  | ✓ |  |  | 2008 | 2008 | <1 |
| Moreno, Christakis, Egan, Brockman and Becker [324] | 2012 | US |  |  |  | ✓ | ✓ |  | ✓ | ✓ |  |  |  |  |  |  |  |  |  |  |  |  |  |  |  |  | ✓ |  | ✓ | ✓ |  | 2009 | 2010 | 1 |
| Moreno, Kacvinsky, Pumper, Wachowski and Whitehill [325] | 2013 | US |  |  |  | ✓ | ✓ |  | ✓ | ✓ |  |  |  |  |  |  |  |  |  |  |  |  |  |  |  |  | ✓ |  | ✓ |  |  | 2012 | 2012 | <1 |
| Morgenstern, Schoeppe, Campbell, Braam, Stoolmiller and Sargent [326] | 2015 | US |  | ✓ |  |  | ✓ |  |  | ✓ |  |  |  |  |  |  |  |  |  |  | ✓ |  |  |  |  |  |  |  | ✓ |  |  | 2009 | 2011 | 2 |
| Moriarty, Jensen and Stryker [327] | 2010 | US | ✓ |  |  |  | ✓ |  |  |  |  |  |  |  | ✓ |  |  |  |  |  |  | ✓ |  |  |  |  |  |  | ✓ |  |  | 2003 | 2003 | 1 |
| Morrison, Sutton and Mebane [328] | 2006 | US | ✓ |  |  |  | ✓ |  |  |  | ✓ |  |  |  |  |  |  |  |  |  |  | ✓ |  |  |  |  |  |  | ✓ |  |  | 2001 | 2004 | 3 |
| Moshrefzadeh, Rice, Pederson and Okoli [329] | 2013 | Canada | ✓ |  |  |  | ✓ |  |  |  | ✓ |  |  |  |  |  |  |  |  |  |  | ✓ |  |  |  |  |  |  | ✓ |  |  | 2010 | 2011 | 1 |
| Movahhed, Seifi, Rashed Mohassel, Dorri, Khorakian and Mohammadzadeh [330] | 2014 | Iran |  | ✓ |  |  | ✓ |  |  |  |  | ✓ |  |  |  |  |  |  | ✓ |  | ✓ |  |  |  |  |  |  |  | ✓ |  |  | 2012 | 2012 | <1 |
| Myhre, Saphir, Flora, Howard and Gonzalez [331] | 2002 | US | ✓ |  |  |  | ✓ |  |  | ✓ |  |  |  |  |  |  |  |  |  |  |  | ✓ |  |  |  |  |  |  | ✓ |  |  | 1997 | 1998 | 1 |
| Myrick, Holton, Himelboim and Love [332] | 2016 | US |  |  |  | ✓ | ✓ |  |  |  |  |  |  |  | ✓ |  |  |  |  |  |  |  |  |  |  |  | ✓ |  | ✓ |  |  | 2011 | 2013 | 2 |
| Nagelhout, Putte, Vries, Crone, Fong and Willemsen [333] | 2012 | Netherlands | ✓ |  |  |  | ✓ | ✓ |  |  | ✓ |  |  |  |  |  |  |  |  |  |  | ✓ |  |  |  |  |  |  | ✓ | ✓ |  | 2008 | 2009 | 1 |
| Nagler, Bigman, Ramanadhan, Ramamurthi and Viswanath [334] | 2016 | US | ✓ |  |  |  | ✓ |  |  |  |  |  |  |  | ✓ |  |  |  |  | ✓ |  | ✓ |  |  |  |  |  |  | ✓ |  |  | 2010 | 2011 | <1 |
| Nan, Zhao, Yang and Iles [335] | 2015 | US |  | ✓ |  |  |  | ✓ |  |  | ✓ |  |  |  |  |  |  |  |  |  |  |  |  |  |  |  |  | ✓ |  | ✓ |  | N/A | N/A | N/A |
| Nelson, Pederson, Mowery, Bailey, Sevilimedu, London, Babb and Pechacek [336] | 2015 | US | ✓ |  |  |  | ✓ |  |  |  | ✓ |  |  |  |  |  |  |  |  |  | ✓ | ✓ |  |  |  |  |  |  | ✓ |  |  | 2004 | 2010 | 6 |
| Nicholls [337] | 2012 | UK |  | ✓ |  |  | ✓ |  |  | ✓ |  |  |  |  |  |  |  |  |  |  |  |  |  |  |  |  | ✓ |  | ✓ |  |  | 2011 | 2011 | <1 |
| Nicholson, Kreuter, Lapka, Wellborn, Clark, Sanders-Thompson, Jacobsen and Casey [338] | 2008 | US | ✓ |  |  |  |  | ✓ |  |  |  |  |  |  | ✓ |  |  |  |  | ✓ |  |  |  |  |  |  |  | ✓ |  | ✓ |  | N/A | N/A | N/A |
| Niederdeppe, Davis, Farrelly and Yarsevich [339] | 2007 | US |  | ✓ |  |  | ✓ | ✓ | ✓ |  | ✓ |  |  |  |  |  |  |  |  |  | ✓ |  |  |  |  |  |  |  | ✓ | ✓ |  | 1999 | 2003 | 4 |
| Niederdeppe, Farrelly and Wenter [340] | 2007 | US | ✓ |  |  |  | ✓ |  |  |  |  | ✓ |  | ✓ |  |  |  |  |  |  | ✓ | ✓ |  |  |  |  |  |  | ✓ |  |  | 1998 | 2001 | 3 |
| Niederdeppe, Fowler, Goldstein and Pribble [341] | 2010 | US | ✓ |  |  |  | ✓ |  | ✓ |  |  |  |  |  | ✓ |  |  |  |  |  | ✓ | ✓ |  |  |  |  |  |  | ✓ | ✓ |  | 2012 | 2012 | <1 |
| Niederdeppe, Gollust, Jarlenski, Nathanson and Barry [342] | 2013 | US | ✓ |  |  |  | ✓ | ✓ |  |  |  |  |  |  | ✓ |  |  |  |  |  | ✓ | ✓ |  |  |  |  |  |  | ✓ | ✓ |  | 2009 | 2011 | 2 |
| Niederdeppe, J., R. Avery, S. Byrne and T. Siam | 2016 | US |  | ✓ |  |  | ✓ |  |  |  | ✓ |  |  |  |  |  |  |  |  |  | ✓ |  |  |  |  |  |  |  | ✓ | ✓ |  | 1998 | 2004 | 6 |
| Niederdeppe, Lee, Robbins, Kim, Kresovich, et al. [343] | 2014 | US | ✓ |  |  |  | ✓ |  |  |  | ✓ |  |  |  |  |  |  |  |  |  |  | ✓ |  |  |  |  |  |  | ✓ |  |  | 2002 | 2002 | <1 |
| Niederkrotenthaler, Voracek, Herberth, Till, Strauss, Etzersdorfer, Eisenwort and Sonneck [344] | 2010 | Austria | ✓ |  |  |  | ✓ |  |  |  |  |  |  |  |  |  |  |  | ✓ |  |  | ✓ |  |  |  |  |  |  | ✓ | ✓ |  | 2005 | 2005 | <1 |
| Nixon, Mejia, Cheyne, Wilking, Dorfman and Daynard [345] | 2015 | US | ✓ |  |  |  | ✓ |  |  |  |  | ✓ |  | ✓ |  |  |  |  |  |  |  | ✓ |  |  |  |  |  |  | ✓ |  |  | 2000 | 2012 | 12 |
| No, Kelly, Devi, Swinburn and Vandevijvere [346] | 2014 | New Zealand |  | ✓ |  |  | ✓ |  |  |  |  | ✓ |  |  |  |  |  |  |  |  |  |  | ✓ |  |  |  |  |  | ✓ |  |  | 2012 | 2014 | 2 |
| O'Connor and Casey [347] | 2001 | Ireland | ✓ |  |  |  | ✓ |  |  |  |  |  |  |  |  |  |  |  | ✓ |  |  | ✓ |  |  |  |  |  |  | ✓ |  |  | 1999 | 1999 | <1 |
| Okuhara, Ishikawa, Okada and Kiuchi [348] | 2014 | Japan | ✓ |  |  |  | ✓ |  |  |  |  |  |  |  | ✓ |  |  |  |  |  |  | ✓ |  |  |  |  |  |  | ✓ |  |  | 2013 | 2013 | <1 |
| Ortiz, Zimmerman and Adler [349] | 2016 | US | ✓ |  |  |  |  | ✓ |  |  |  |  |  | ✓ |  |  |  |  |  |  |  |  |  |  |  |  |  | ✓ |  | ✓ |  | N/A | N/A | N/A |
| Ortiz, Zimmerman and Gilliam [350] | 2015 | US | ✓ |  |  |  |  |  | ✓ |  |  |  |  | ✓ |  |  |  |  |  |  |  |  |  |  |  |  |  |  | ✓ |  |  | N/A | N/A | N/A |
| Outley and Taddese [351] | 2006 | US |  | ✓ |  |  | ✓ |  |  |  |  | ✓ | ✓ |  |  |  |  |  |  |  | ✓ |  |  |  |  |  |  |  | ✓ |  |  | 2005 | 2005 | <1 |
| Paek, Hove and Jeon [352] | 2013 | Korea |  |  |  | ✓ | ✓ |  |  |  | ✓ |  |  |  |  |  |  |  |  |  |  |  |  |  |  |  | ✓ |  | ✓ | ✓ |  | N/A | N/A | N/A |
| Paek, Kim and Hove [356] | 2010 | US |  |  |  | ✓ | ✓ |  |  |  | ✓ |  |  |  |  |  |  |  |  |  |  |  |  |  |  |  | ✓ |  | ✓ |  |  | N/A | N/A | N/A |
| Paek, Kim, Hove and Huh [353] | 2014 | US |  |  |  | ✓ | ✓ |  |  |  | ✓ |  |  |  |  |  |  |  |  |  |  |  |  |  |  |  | ✓ |  | ✓ |  |  | 2007 | 2011 | 4 |
| Paek, Reid, Choi and Jeong [354] | 2010 | US |  | ✓ |  |  | ✓ |  |  |  | ✓ |  |  |  |  |  |  |  |  |  |  |  | ✓ |  |  |  |  |  | ✓ |  |  | 1954 | 2003 | 49 |
| Paek, Reid, Jeong, Choi and Krugman [355] | 2012 | US |  | ✓ |  |  | ✓ |  |  |  | ✓ |  |  |  |  |  |  |  |  |  |  |  | ✓ |  |  |  |  |  | ✓ |  |  | 1954 | 2003 | 49 |
| Page and Brewster [357] | 2007 | US |  | ✓ |  |  | ✓ |  |  |  |  | ✓ |  |  |  |  |  |  |  |  | ✓ |  |  |  |  |  |  |  | ✓ |  |  | 2004 | 2005 | 1 |
| Park and Morton [360] | 2015 | US |  | ✓ |  |  |  | ✓ |  | ✓ |  |  |  |  |  |  |  |  |  |  |  |  |  |  |  |  |  | ✓ |  | ✓ |  | N/A | N/A | N/A |
| Park and Reber [358] | 2010 | US | ✓ |  |  |  | ✓ |  |  |  |  |  |  |  | ✓ | ✓ | ✓ |  |  |  |  | ✓ |  |  |  |  |  |  | ✓ |  |  | 2005 | 2006 | 1 |
| Park, Reber and Chon [359] | 2016 | US |  |  |  | ✓ | ✓ |  |  |  |  |  |  |  | ✓ | ✓ | ✓ |  |  |  |  |  |  |  |  |  | ✓ |  | ✓ |  |  | 2013 | 2013 | <1 |
| Parke, Ashcroft, Brown, Marteau and Seale [361] | 2013 | UK | ✓ |  |  |  | ✓ |  |  |  |  |  |  |  |  |  |  |  | ✓ |  |  | ✓ |  |  |  |  | ✓ |  | ✓ |  |  | 2005 | 2010 | 5 |
| Patterson, Katikireddi, Wood and Hilton [362] | 2015 | UK & Scotland | ✓ |  |  |  | ✓ |  |  | ✓ |  |  |  |  |  |  |  |  |  |  |  | ✓ |  |  |  |  |  |  | ✓ |  |  | 2004 | 2014 | 10 |
| Patterson, Semple, Wood, Duffy and Hilton [363] | 2015 | UK | ✓ |  |  |  | ✓ |  |  |  | ✓ |  |  |  |  |  |  |  |  |  |  | ✓ |  |  |  |  |  |  | ✓ |  |  | 2005 | 2012 | 7 |
| Pendleton, Smith and Roberts [364] | 1991 | UK |  |  | ✓ |  | ✓ |  |  | ✓ |  |  |  |  |  |  |  |  |  |  | ✓ |  |  |  |  |  |  |  | ✓ |  |  | 1988 | 1988 | <1 |
| Peng and Tang [365] | 2010 | China | ✓ |  |  |  | ✓ |  |  |  |  |  |  |  |  |  |  |  | ✓ |  |  | ✓ |  |  |  |  |  |  | ✓ |  |  | 2007 | 2007 | <1 |
| Phoutthakeo, Otsuka, Ito, Sayamoungkhoun, Kounnavong and Jimba [366] | 2014 | Laos |  | ✓ |  |  | ✓ |  | ✓ |  |  | ✓ |  |  |  |  |  |  | ✓ |  | ✓ |  |  |  |  |  |  |  | ✓ | ✓ |  | 2010 | 2010 | <1 |
| Pinsky and Silva [367] | 1999 | Brazil |  | ✓ |  |  | ✓ |  |  | ✓ |  |  |  |  |  |  |  |  |  |  | ✓ |  |  |  |  |  |  |  | ✓ |  |  | 1992 | 1993 | 1 |
| Poston, Haddock, Jahnke, Hyder and Jitnarin [368] | 2015 | US | ✓ |  |  |  | ✓ |  |  | ✓ | ✓ | ✓ | ✓ | ✓ | ✓ |  |  |  | ✓ |  |  | ✓ |  |  |  |  |  |  | ✓ |  |  | 2012 | 2012 | <1 |
| Potter, Sheeshka and Valaitis [369] | 2000 | Canada | ✓ | ✓ |  |  | ✓ |  |  |  |  | ✓ |  |  |  |  |  |  | ✓ |  |  |  | ✓ |  |  |  |  |  | ✓ |  |  | 1945 | 1995 | 50 |
| Potvin Kent, Dubois and Wanless [370] | 2011 | Canada |  | ✓ |  |  | ✓ |  |  |  |  | ✓ |  |  |  |  |  |  |  |  | ✓ |  |  |  |  |  |  |  |  | ✓ | ✓ | 2009 | 2009 | <1 |
| Potvin Kent, Dubois and Wanless [371] | 2012 | Canada |  | ✓ |  |  | ✓ |  |  |  |  | ✓ |  |  |  |  |  |  |  |  | ✓ |  |  |  |  |  |  |  | ✓ | ✓ |  | 2009 | 2009 | <1 |
| Potvin Kent, Martin and Kent [372] | 2014 | Canada |  | ✓ |  |  | ✓ |  |  |  |  | ✓ |  |  |  |  |  |  |  |  | ✓ |  |  |  |  |  |  |  | ✓ |  |  | 2006 | 2011 | 5 |
| Pounders, Lee and Mackert [373] | 2015 | US |  | ✓ |  |  |  | ✓ |  |  |  |  |  |  | ✓ | ✓ |  |  |  |  |  |  |  |  |  |  |  | ✓ |  | ✓ |  | N/A | N/A | N/A |
| Pribble, Goldstein, Fowler, Greenberg, Noel and Howell [374] | 2006 | US | ✓ |  |  |  | ✓ |  |  |  |  |  |  |  |  |  |  |  | ✓ |  | ✓ |  |  |  |  |  |  |  | ✓ |  |  | 2002 | 2002 | <1 |
| Pribble, Goldstein, Majersik, Barsan, Brown and Morgenstern [375] | 2006 | US | ✓ |  |  |  | ✓ |  |  |  |  |  |  |  |  |  |  |  | ✓ |  | ✓ |  |  |  |  |  |  |  | ✓ |  |  | 2002 | 2002 | <1 |
| Price and Grann [376] | 2012 | US | ✓ |  |  |  | ✓ |  |  |  |  |  |  |  |  |  |  |  | ✓ |  |  |  |  |  |  |  | ✓ |  | ✓ |  |  | 2010 | 2010 | <1 |
| Primack, Nuzzo, Rice and Sargent [377] | 2012 | US |  |  | ✓ |  | ✓ |  |  | ✓ |  |  |  |  |  |  |  |  |  |  |  |  | ✓ |  |  | ✓ |  |  | ✓ |  |  | 2005 | 2007 | 2 |
| Puhl, Peterson and Luedicke [378] | 2013 | US |  | ✓ |  |  | ✓ |  | ✓ |  |  |  |  | ✓ |  |  |  |  |  |  |  |  |  |  |  |  |  | ✓ | ✓ | ✓ |  | N/A | N/A | N/A |
| Puhl, Peterson, DePierre and Luedicke [379] | 2013 | US | ✓ |  |  |  | ✓ |  |  |  |  |  |  | ✓ |  |  |  |  |  |  |  |  |  |  |  |  | ✓ |  | ✓ |  |  | 2010 | 2010 | <1 |
| Pujazon-Zazik, Manasse and Orrell-Valente [380] | 2012 | US |  |  |  | ✓ | ✓ |  |  | ✓ | ✓ |  |  |  |  |  |  |  |  |  |  |  |  |  |  |  | ✓ |  | ✓ |  |  | 2009 | 2009 | <1 |
| Rachul and Caulfield [381] | 2015 | Canada | ✓ |  |  |  | ✓ |  |  |  |  |  |  |  |  |  |  |  | ✓ |  |  | ✓ |  |  |  |  |  |  | ✓ |  |  | 2003 | 2012 | 9 |
| Ramanadhan, Mendez, Rao and Viswanath [382] | 2013 | US |  |  |  | ✓ | ✓ |  |  |  |  |  |  |  |  |  |  |  | ✓ |  |  |  |  |  |  |  | ✓ |  | ✓ |  |  | 2011 | 2012 | 1 |
| Ramos and Navas [383] | 2015 | Spain |  | ✓ |  |  | ✓ |  |  |  |  | ✓ |  |  |  |  |  |  |  |  | ✓ |  |  |  |  |  |  |  | ✓ |  |  | 2013 | 2013 | <1 |
| Rayens, Butler, Wiggins, Kostygina, Langley and Hahn [384] | 2016 | US |  | ✓ |  |  |  | ✓ | ✓ |  | ✓ |  |  |  |  |  |  |  |  |  |  | ✓ | ✓ |  |  |  |  | ✓ |  | ✓ |  | 2010 | 2011 | 1 |
| Reinau, Meier, Blumenthal and Surber [385] | 2016 | Germany, Switzerland | ✓ |  |  |  | ✓ |  |  |  |  |  |  |  | ✓ |  |  |  |  |  |  | ✓ | ✓ |  |  |  |  |  | ✓ |  |  | 2012 | 2013 | 1 |
| Rhoades and Jernigan [386] | 2013 | US |  | ✓ |  |  | ✓ |  |  | ✓ |  |  |  |  |  |  |  |  |  |  |  |  | ✓ |  |  |  |  |  | ✓ |  |  | 2003 | 2007 | 5 |
| Rhodes, Roskos-Ewoldsen, Eno and Monahan [387] | 2009 | US |  | ✓ |  |  | ✓ |  |  |  | ✓ |  |  |  |  |  |  |  |  |  | ✓ |  |  |  |  |  |  |  | ✓ |  |  | 1998 | 2003 | 5 |
| Ribisl, Lee, Henriksen and Haladjian [388] | 2003 | US |  | ✓ |  |  | ✓ |  |  |  | ✓ |  |  |  |  |  |  |  |  |  |  |  |  |  |  |  | ✓ |  | ✓ |  |  | 1999 | 1999 | <1 |
| Riker, Butler, Ricks, Record, Begley, Anderson and Hahn [389] | 2015 | US |  | ✓ |  |  | ✓ |  | ✓ |  | ✓ |  |  |  |  |  |  |  |  |  |  |  |  |  |  |  |  |  | ✓ | ✓ |  | N/A | N/A | N/A |
| Riles, Sangalang, Hurley and Tewksbury [390] | 2015 | US | ✓ |  |  |  | ✓ | ✓ |  |  |  |  |  |  | ✓ |  |  |  |  |  |  |  |  |  |  |  | ✓ | ✓ | ✓ | ✓ |  | 2008 | 2012 | 4 |
| Rissel, Bonfiglioli, Emilsen and Smith [391] | 2010 | Australia | ✓ |  |  |  | ✓ |  |  |  |  |  | ✓ |  |  |  |  |  |  |  |  | ✓ |  |  |  |  |  |  | ✓ |  |  | 1998 | 2008 | 10 |
| Roberts, Pettigrew, Chapman, Quester and Miller [392] | 2013 | Australia |  | ✓ |  |  | ✓ |  |  |  |  | ✓ |  |  |  |  |  |  |  |  | ✓ |  |  |  |  |  |  |  | ✓ |  |  | 2010 | 2010 | <1 |
| Robinson, Callister and Jankoski [393] | 2008 | US |  |  | ✓ |  | ✓ |  |  |  |  |  |  | ✓ |  |  |  |  |  |  | ✓ |  |  |  |  |  |  |  | ✓ |  |  | 2005 | 2005 | <1 |
| Rose, Friedman, Marquez and Fernandez [394] | 2013 | US | ✓ |  |  |  | ✓ |  |  |  |  |  | ✓ |  |  |  |  |  |  |  |  |  | ✓ |  |  |  |  |  | ✓ |  |  | 2009 | 2010 | 1 |
| Roseman, Poor and Stephenson [395] | 2014 | US |  |  | ✓ |  | ✓ |  |  |  |  | ✓ |  |  |  |  |  |  |  |  | ✓ |  |  |  |  |  |  |  | ✓ |  |  | 2007 | 2007 | <1 |
| Saguy, Gruys and Gong [396] | 2010 | US & France | ✓ |  |  |  | ✓ |  |  |  |  |  |  | ✓ |  |  |  |  |  |  |  | ✓ |  |  |  |  |  |  | ✓ |  |  | 1995 | 2005 | 10 |
| Schneider, McGovern, Lynch and Brown [397] | 2013 | US |  |  |  | ✓ | ✓ |  |  |  |  | ✓ |  |  |  |  |  |  |  |  |  |  |  |  |  |  | ✓ |  | ✓ |  |  | 2010 | 2011 | 1 |
| Schulz and Hartung [398] | 2011 | Switzerland | ✓ |  |  |  | ✓ |  |  |  |  | ✓ |  |  |  |  |  |  | ✓ |  |  | ✓ | ✓ |  |  |  |  |  | ✓ |  |  | 2003 | 2005 | 2 |
| Scully, Macken, Leddin, Cullen, Dunne and Gorman [401] | 2015 | Ireland |  | ✓ |  |  | ✓ |  |  |  |  | ✓ |  |  |  |  |  |  |  |  | ✓ |  |  |  |  |  |  |  | ✓ |  |  | 2010 | 2010 | <1 |
| Scully, Makin, Maloney and Wakefield [399] | 2014 | Australia | ✓ |  |  |  | ✓ |  |  |  |  |  |  |  | ✓ |  |  |  |  |  |  | ✓ |  |  |  |  |  |  | ✓ |  |  | 2001 | 2012 | 12 |
| Scully, Wakefield and Dixon [400] | 2008 | Australia | ✓ |  |  |  | ✓ |  |  |  |  |  |  |  | ✓ |  |  |  |  |  |  | ✓ |  |  |  |  |  |  | ✓ |  |  | 1993 | 2006 | 13 |
| Segar, Updegraff, Zikmund-Fisher and Richardson [402] | 2012 | US |  | ✓ |  |  |  | ✓ |  |  |  |  | ✓ |  |  |  |  |  |  |  |  |  |  |  |  |  |  | ✓ |  | ✓ |  | N/A | N/A | N/A |
| Seidenberg, Rodgers, Rees and Connolly [403] | 2012 | US |  |  |  | ✓ | ✓ |  |  |  | ✓ |  |  |  |  |  |  |  |  |  |  |  |  |  |  |  | ✓ |  | ✓ |  |  | 2009 | 2009 | <1 |
| Sepe and Glantz [404] | 2002 | US |  | ✓ |  |  | ✓ |  |  |  | ✓ |  |  |  |  |  |  |  |  |  |  | ✓ |  |  |  |  |  |  | ✓ |  |  | 1994 | 1999 | 5 |
| Shea and Chapman [405] | 2001 | Australia | ✓ |  |  |  | ✓ |  |  |  |  |  |  |  |  |  |  |  | ✓ |  |  | ✓ |  |  |  |  |  |  | ✓ |  |  | 1988 | 1999 | 11 |
| Shim, Kim, Kye and Park [406] | 2016 | Korea | ✓ |  |  |  | ✓ |  |  |  |  |  |  |  | ✓ |  |  |  |  |  | ✓ | ✓ |  |  |  |  |  |  | ✓ |  |  | 2008 | 2012 | 5 |
| Shugart [407] | 2011 | US | ✓ |  |  |  | ✓ |  |  |  |  |  |  | ✓ |  |  |  |  |  |  | ✓ | ✓ | ✓ |  |  |  |  |  | ✓ |  |  | 2008 | 2009 | 1 |
| Shugart [408] | 2013 | US | ✓ |  |  |  | ✓ |  |  |  |  |  |  | ✓ |  |  |  |  |  |  | ✓ | ✓ |  |  |  |  |  |  | ✓ |  |  | 2009 | 2012 | 3 |
| Silver Wallace and Leenders [409] | 2004 | US | ✓ |  |  |  | ✓ |  |  |  |  |  | ✓ |  |  |  |  |  |  |  | ✓ |  |  |  |  |  |  |  | ✓ |  |  | 1970 | 2001 | 31 |
| Simunaniemi, Sandberg, Andersson and Nydahl [410] | 2011 | Sweden |  |  |  | ✓ | ✓ |  |  |  |  | ✓ |  |  |  |  |  |  |  |  |  |  |  |  |  |  | ✓ |  | ✓ |  |  | 2009 | 2009 | <1 |
| Siu [411] | 2009 | US | ✓ |  |  |  | ✓ |  |  |  | ✓ |  |  |  |  |  |  |  |  |  |  | ✓ |  |  |  |  |  |  | ✓ |  |  | 1964 | 1998 | 34 |
| Sixsmith and Furnham [412] | 2010 | UK |  | ✓ |  |  | ✓ |  |  |  |  | ✓ |  |  |  |  |  |  |  |  | ✓ |  |  |  |  |  |  |  | ✓ |  |  | 2008 | 2008 | <1 |
| Slater, Long, Bettinghaus and Reineke [413] | 2008 | US | ✓ |  |  |  | ✓ |  |  |  |  |  |  |  | ✓ |  |  |  |  |  | ✓ | ✓ | ✓ |  |  |  |  |  | ✓ |  |  | 2002 | 2003 | 1 |
| Sloane, Wilson and Imlach Gunasekara [414] | 2013 | New Zealand |  |  | ✓ |  | ✓ |  |  | ✓ |  |  |  |  |  |  |  |  |  |  |  |  |  |  |  | ✓ |  |  | ✓ |  |  | 2005 | 2010 | 5 |
| Smith [416] | 2007 | US | ✓ |  |  |  | ✓ |  |  |  | ✓ |  |  |  |  |  |  |  |  |  |  | ✓ |  |  |  |  |  |  | ✓ |  |  | 1989 | 2005 | 16 |
| Smith [417] | 2012 | US |  | ✓ |  |  | ✓ |  |  |  | ✓ | ✓ |  |  |  |  |  |  | ✓ |  |  |  |  |  |  |  | ✓ |  | ✓ |  |  | 2007 | 2009 | 2 |
| Smith and Bonfiglioli [415] | 2015 | Australia | ✓ |  | ✓ |  |  |  | ✓ |  |  |  | ✓ |  |  |  |  |  |  |  |  |  |  |  |  |  |  |  |  | ✓ |  | N/A | N/A | N/A |
| Smith and Wakefield [421] | 2005 | US | ✓ |  |  |  | ✓ |  |  |  | ✓ |  |  |  |  |  |  |  |  |  |  | ✓ |  |  |  |  |  |  | ✓ |  |  | 2001 | 2001 | <1 |
| Smith, Cukier and Jernigan [419] | 2014 | US |  | ✓ |  |  | ✓ |  |  | ✓ |  |  |  |  |  |  |  |  |  |  |  |  | ✓ |  |  |  |  |  | ✓ |  |  | 2008 | 2010 | 2 |
| Smith, McLeod and Wakefield [420] | 2005 | Australia | ✓ |  |  |  | ✓ | ✓ |  |  | ✓ |  |  |  |  |  |  |  |  |  |  | ✓ |  |  |  |  |  |  | ✓ | ✓ |  | 2001 | 2003 | 2 |
| Smith, Offen and Malone [418] | 2005 | US |  | ✓ |  |  | ✓ |  |  |  | ✓ |  |  |  |  |  |  |  |  |  |  |  | ✓ |  |  |  |  |  | ✓ |  |  | 1990 | 2000 | 10 |
| Smith, Wakefield, Terry-McElrath, Chaloupka, Flay, Johnston, Saba and Siebel [422] | 2008 | US | ✓ |  |  |  | ✓ |  |  |  | ✓ |  |  |  |  |  |  |  |  |  |  | ✓ |  |  |  |  |  |  | ✓ |  |  | 2001 | 2003 | 2 |
| Snider [423] | 2010 | US | ✓ |  |  |  | ✓ |  |  |  |  |  |  | ✓ |  |  |  |  |  |  |  |  | ✓ |  |  |  |  |  | ✓ |  |  | 1974 | 2009 | 35 |
| Soo, Letona, Chacon, Barnoya and Roberto [424] | 2016 | Guatemala |  | ✓ |  |  | ✓ |  |  |  |  | ✓ |  |  |  |  |  |  |  |  |  |  |  |  |  |  |  | ✓ | ✓ |  |  | 2013 | 2013 | <1 |
| Spencer, Russell and Barker [425] | 2014 | UK | ✓ | ✓ |  |  | ✓ |  |  |  |  | ✓ |  | ✓ |  |  |  |  |  |  |  |  | ✓ |  |  |  |  |  | ✓ |  |  | 1999 | 2011 | 12 |
| Squiers, Holden, Dolina, Kim, Bann and Renaud [426] | 2011 | US | ✓ |  |  | ✓ | ✓ | ✓ |  |  |  |  |  |  | ✓ |  |  |  |  |  |  | ✓ |  |  |  |  | ✓ |  | ✓ | ✓ |  | 2009 | 2010 | 1 |
| Stefanik-Sidener [427] | 2013 | US | ✓ |  |  |  | ✓ |  |  |  |  |  |  |  |  |  | ✓ |  |  |  | ✓ |  |  |  |  |  |  |  | ✓ |  |  | 2000 | 2010 | 10 |
| Stellefson, Chaney, Ochipa, Chaney, Haider, Hanik, Chavarria and Bernhardt [428] | 2014 | US |  |  |  | ✓ | ✓ |  |  |  |  |  |  |  |  |  |  | ✓ |  |  |  |  |  |  |  |  | ✓ |  | ✓ |  |  | 2012 | 2012 | <1 |
| Sterling, Fryer, Majeed and Duong [429] | 2015 | US |  | ✓ |  |  | ✓ |  |  |  | ✓ |  |  |  |  |  |  |  |  |  |  | ✓ |  |  |  |  |  |  | ✓ |  |  | 2011 | 2012 | <1 |
| Stitt and Kunkel [430] | 2008 | US |  | ✓ |  |  | ✓ |  |  |  |  | ✓ |  |  |  |  |  |  |  |  | ✓ |  |  |  |  |  |  |  | ✓ |  |  | 2005 | 2005 | <1 |
| Story and Faulkner [431] | 1990 | US |  | ✓ | ✓ |  | ✓ |  |  |  |  | ✓ |  |  |  |  |  |  |  |  | ✓ |  |  |  |  |  |  |  | ✓ |  |  | 1988 | 1988 | <1 |
| Street [432] | 2004 | Australia |  | ✓ |  |  | ✓ |  |  |  | ✓ |  |  |  |  |  |  |  |  |  |  |  | ✓ |  |  |  |  |  | ✓ |  |  | 1946 | 2004 | 58 |
| Stryker, Fishman, Emmons and Viswanath [433] | 2009 | US | ✓ |  |  |  | ✓ |  |  |  |  |  |  |  | ✓ |  |  |  |  |  |  | ✓ |  |  |  |  |  |  | ✓ |  |  | 2003 | 2003 | <1 |
| Stryker, Moriarty and Jensen [434] | 2008 | US | ✓ |  |  |  | ✓ | ✓ |  |  |  |  |  |  | ✓ |  |  |  |  |  |  | ✓ |  |  |  |  |  |  | ✓ | ✓ |  | 2003 | 2003 | <1 |
| Stryker, Solky and Emmons [435] | 2005 | US | ✓ |  |  |  | ✓ |  |  |  |  |  |  |  | ✓ |  |  |  |  |  |  | ✓ |  |  |  |  |  |  | ✓ |  |  | 1979 | 2003 | 24 |
| Sukumaran, Diwakar and Shastry [436] | 2012 | India |  | ✓ |  |  | ✓ |  |  |  |  | ✓ |  |  |  |  |  |  |  |  | ✓ |  |  |  |  |  |  |  | ✓ |  |  | 2010 | 2011 | 1 |
| Sun, Krakow, John, Liu and Weaver [437] | 2016 | US | ✓ |  |  |  |  | ✓ |  |  |  |  |  | ✓ |  |  |  |  |  |  |  |  |  |  |  |  |  | ✓ |  | ✓ |  | N/A | N/A | N/A |
| Syed-Abdul, Fernandez-Luque, Jian, Li, Crain, et al. [438] | 2013 | Taiwan |  |  |  | ✓ | ✓ |  |  |  |  |  |  | ✓ |  |  |  |  | ✓ |  |  |  |  |  |  |  | ✓ |  | ✓ |  |  | 2011 | 2011 | <1 |
| Szklo and Coutinho [439] | 2010 | Brazil |  | ✓ |  |  | ✓ | ✓ |  |  | ✓ |  |  |  |  |  |  |  |  |  |  |  |  |  |  |  |  | ✓ | ✓ | ✓ |  | 2008 | 2008 | <1 |
| Team and Markovic [440] | 2006 | Australia |  | ✓ |  |  | ✓ |  |  |  |  |  |  |  | ✓ |  |  |  |  |  |  |  |  |  |  |  | ✓ |  | ✓ |  |  | N/A | N/A | N/A |
| Thackeray, Burton, Giraud-Carrier, Rollins and Draper [441] | 2013 | US |  |  |  | ✓ | ✓ |  |  |  |  |  |  |  | ✓ |  |  |  |  |  |  |  |  |  |  |  | ✓ |  | ✓ |  |  | 2012 | 2012 | <1 |
| Theberge [442] | 1991 | Canada | ✓ |  |  |  | ✓ |  |  |  |  |  | ✓ |  |  |  |  |  |  |  |  | ✓ | ✓ |  |  |  |  |  | ✓ |  |  | 1977 | 1987 | 10 |
| Thomas, Olds, Pettigrew, Yeatman, Hyde and Dragovic [443] | 2014 | Australia |  | ✓ |  |  | ✓ | ✓ | ✓ |  |  |  |  | ✓ |  |  |  |  |  |  | ✓ |  |  |  |  |  |  |  | ✓ | ✓ |  | 2011 | 2012 | 1 |
| Thompson, Flores, Ebel and Christakis [444] | 2008 | US |  | ✓ |  |  | ✓ |  |  |  |  | ✓ |  |  |  |  |  |  |  |  | ✓ |  |  |  |  |  |  |  | ✓ |  |  | 2006 | 2006 | <1 |
| Thompson, Robinson, Cusella and Shellabarger [445] | 2000 | US |  |  | ✓ |  | ✓ |  |  |  |  |  |  |  |  |  |  |  | ✓ |  | ✓ |  |  |  |  |  |  |  | ✓ |  |  | 1985 | 1999 | 14 |
| Thornley, Signal and Thomson [446] | 2010 | New Zealand |  | ✓ |  |  | ✓ |  |  |  |  | ✓ |  |  |  |  |  |  |  |  |  |  |  |  |  |  | ✓ |  | ✓ |  |  | 2005 | 2006 | 1 |
| Thrasher, Kim, Rose, Navarro, Craft, Davis and Biggers [447] | 2014 | US | ✓ |  |  |  | ✓ |  |  |  | ✓ |  |  |  |  |  |  |  |  |  |  | ✓ |  |  |  |  |  |  | ✓ |  |  | 2006 | 2010 | 4 |
| Till and Niederkrotenthaler [448] | 2014 | US, Austria | ✓ |  |  |  | ✓ |  |  |  |  |  |  |  |  |  |  |  | ✓ |  |  |  |  |  |  |  | ✓ |  | ✓ |  |  | 2013 | 2013 | <1 |
| Timberlake, Pechmann, Tran and Au [449] | 2011 | US |  | ✓ |  |  | ✓ |  |  |  | ✓ |  |  |  |  |  |  |  |  |  |  | ✓ | ✓ |  |  |  |  |  | ✓ |  |  | 2007 | 2010 | 3 |
| Toll, Salovey, O'Malley, Mazure, Latimer and McKee [450] | 2008 | US |  | ✓ |  |  |  | ✓ |  |  | ✓ |  |  |  |  |  |  |  |  |  |  |  |  |  |  |  |  | ✓ |  | ✓ |  | N/A | N/A | N/A |
| Tong, Chapman, Sainsbury and Craig [451] | 2008 | Australia | ✓ |  |  |  | ✓ |  |  |  |  |  |  |  |  |  |  | ✓ |  |  | ✓ | ✓ |  |  |  |  |  |  | ✓ |  |  | 2005 | 2007 | 2 |
| Toohey and Rock [452] | 2015 | Canada | ✓ |  |  |  | ✓ |  |  |  |  |  | ✓ |  |  |  |  |  | ✓ |  |  | ✓ |  |  |  |  |  |  | ✓ |  |  | 2006 | 2012 | 6 |
| Tyrrell [453] | 2000 | Australia |  | ✓ |  |  | ✓ |  |  |  | ✓ |  |  |  |  |  |  |  |  |  |  | ✓ |  |  |  |  |  |  | ✓ |  |  | 1927 | 1937 | 10 |
| Ulijaszek and McLennan [454] | 2016 | UK | ✓ |  |  |  | ✓ |  |  |  |  |  |  | ✓ |  |  |  |  |  |  |  |  |  |  |  |  |  | ✓ | ✓ |  |  | 1997 | 2015 | 18 |
| Van Den Bulck, Simons and Gorp [455] | 2008 | US |  |  | ✓ |  | ✓ |  |  | ✓ |  |  |  |  |  |  |  |  |  |  | ✓ |  |  |  |  |  |  |  | ✓ |  |  | 2003 | 2004 | 1 |
| van der Wardt, Taal, Rasker and Wiegman [456] | 1999 | Netherlands | ✓ |  |  |  | ✓ |  |  |  |  |  |  |  | ✓ | ✓ | ✓ | ✓ |  |  | ✓ | ✓ | ✓ |  |  |  |  |  | ✓ |  |  | 1992 | 1993 | 1 |
| van Hoof, de Jong, Fennis and Gosselt [458] | 2009 | Netherlands |  |  | ✓ |  | ✓ | ✓ |  | ✓ |  |  |  |  |  |  |  |  |  |  | ✓ |  |  |  |  |  |  |  | ✓ | ✓ |  | 2004 | 2004 | <1 |
| van Hoof, van Noordenburg and de Jong [457] | 2008 | Netherlands |  | ✓ |  |  | ✓ |  | ✓ | ✓ |  |  |  |  |  |  |  |  |  |  |  |  |  |  |  |  | ✓ |  | ✓ |  |  | 2006 | 2006 | <1 |
| van Kleef, van Trijp and Luning [459] | 2005 | Netherlands |  | ✓ |  |  |  | ✓ |  |  |  | ✓ |  |  |  |  |  |  |  |  |  |  |  |  |  |  |  | ✓ |  | ✓ |  | N/A | N/A | N/A |
| Wackowski, Lewis and Hrywna [461] | 2011 | US | ✓ |  |  |  | ✓ |  |  |  | ✓ |  |  |  |  |  |  |  |  |  |  | ✓ |  |  |  |  |  |  | ✓ |  |  | 2005 | 2007 | 2 |
| Wackowski, Lewis, Delnevo and Ling [460] | 2013 | US | ✓ |  |  |  | ✓ |  |  |  | ✓ |  |  |  |  |  |  |  |  |  |  | ✓ |  |  |  |  |  |  | ✓ |  |  | 2006 | 2010 | 4 |
| Wakefield, Brennan, Durkin, McLeod and Smith [464] | 2011 | Australia | ✓ |  |  |  | ✓ |  |  |  | ✓ |  |  |  |  |  |  |  |  |  |  | ✓ |  |  |  |  |  |  | ✓ |  |  | 2001 | 2006 | 5 |
| Wakefield, Brennan, Durkin, McLeod and Smith [465] | 2012 | Australia | ✓ |  |  |  | ✓ |  |  |  | ✓ |  |  |  |  |  |  |  |  |  |  | ✓ |  |  |  |  |  |  | ✓ |  |  | 2004 | 2007 | 3 |
| Wakefield, McLeod and Smith [462] | 2003 | Australia | ✓ |  |  |  | ✓ |  |  |  | ✓ |  |  |  |  |  |  |  |  |  |  | ✓ |  |  |  |  |  |  | ✓ |  |  | 2002 | 2002 | <1 |
| Wakefield, Smith and Chapman [463] | 2005 | Australia | ✓ |  |  |  | ✓ |  |  |  | ✓ |  |  |  |  |  |  |  |  |  |  | ✓ |  |  |  |  |  |  | ✓ |  |  | 2001 | 2001 | <1 |
| Wallack and Dorfman [466] | 1992 | US |  | ✓ |  |  | ✓ |  |  |  |  | ✓ |  |  |  |  |  |  | ✓ |  | ✓ |  |  |  |  |  |  |  | ✓ |  |  | 1989 | 1989 | <1 |
| Wallington, Blake, Taylor-Clark and Viswanath [468] | 2010 | US | ✓ |  |  |  |  |  | ✓ |  |  |  |  |  |  |  |  |  | ✓ | ✓ |  |  |  |  |  |  |  |  |  |  | ✓ | N/A | N/A | N/A |
| Wallington, Blake, Taylor-Clark and Viswanath [467] | 2010 | US | ✓ |  |  |  |  |  | ✓ |  |  |  |  |  |  |  |  |  | ✓ |  |  |  |  |  |  |  |  |  |  |  | ✓ | N/A | N/A | N/A |
| Walter [469] | 2010 | UK | ✓ |  |  |  | ✓ |  |  |  |  |  |  |  | ✓ |  |  |  |  |  |  | ✓ | ✓ |  |  |  |  |  | ✓ |  |  | 2009 | 2009 | <1 |
| Warner [470] | 1985 | US |  | ✓ |  |  | ✓ |  |  |  | ✓ |  |  |  |  |  |  |  |  |  |  |  | ✓ |  |  |  |  |  | ✓ |  |  | 1929 | 1984 | 55 |
| Warsh and Tinkler [471] | 2007 | US | ✓ |  |  |  | ✓ |  |  |  | ✓ |  |  |  |  |  |  |  |  |  |  |  | ✓ |  |  |  |  |  | ✓ |  |  | 1920 | 1960 | 40 |
| Webb, Baker and Rodriguez de Ybarra [472] | 2010 | US |  | ✓ |  |  |  | ✓ |  |  | ✓ |  | ✓ |  |  |  |  |  |  |  |  |  |  |  |  |  |  |  |  | ✓ |  | N/A | N/A | N/A |
| Weeks, Friedenberg, Southwell and Slater [473] | 2012 | US | ✓ |  |  |  | ✓ |  |  |  |  |  |  |  | ✓ |  |  |  |  |  | ✓ | ✓ |  |  |  |  | ✓ |  | ✓ | ✓ |  | 2008 | 2009 | 1 |
| Weeks, Verhoef and Scott [474] | 2007 | Canada | ✓ |  |  |  | ✓ |  |  |  |  |  |  |  | ✓ |  |  |  |  |  |  | ✓ | ✓ |  |  |  |  |  | ✓ |  |  | 1990 | 2005 | 16 |
| Wellard, Hughes, Tsang, Watson and Chapman [475] | 2015 | Australia |  | ✓ |  |  | ✓ |  |  |  |  | ✓ |  |  |  |  |  |  |  |  |  |  |  |  |  |  |  | ✓ | ✓ |  |  | 2013 | 2013 | <1 |
| Wenger, Malone and Bero [476] | 2001 | US | ✓ |  |  |  | ✓ |  |  |  | ✓ |  |  |  |  |  |  |  |  |  |  | ✓ |  |  |  |  |  |  | ✓ |  |  | 1987 | 1997 | 10 |
| Wilkin, Gonzalez and Tannebaum [477] | 2015 | US | ✓ |  |  |  | ✓ |  |  |  |  | ✓ | ✓ | ✓ | ✓ | ✓ | ✓ | ✓ | ✓ |  | ✓ |  |  |  |  |  |  |  | ✓ |  |  | 2005 | 2006 | 1 |
| Williams and Schmidt [478] | 2014 | US |  | ✓ |  |  | ✓ |  |  | ✓ |  |  |  |  |  |  |  |  |  |  |  |  |  |  |  |  | ✓ |  | ✓ |  |  | 2010 | 2010 | <1 |
| Willis and Knobloch-Westerwick [479] | 2014 | US | ✓ |  |  |  | ✓ |  |  |  |  |  | ✓ | ✓ |  |  |  |  |  |  |  |  | ✓ |  |  |  |  |  | ✓ |  |  | 2010 | 2010 | <1 |
| Wilson, Signal, Nicholls and Thomson [480] | 2006 | New Zealand |  | ✓ |  |  | ✓ |  |  |  |  | ✓ |  |  |  |  |  |  |  |  | ✓ |  |  |  |  |  |  |  | ✓ |  |  | 2005 | 2005 | <1 |
| Wilson, Sloane, Gunasekara and Thomson [481] | 2011 | New Zealand |  |  | ✓ |  | ✓ |  |  |  | ✓ |  |  |  |  |  |  |  |  |  | ✓ |  |  |  | ✓ |  |  |  | ✓ |  |  | 2005 | 2010 | 5 |
| Wise and Brewer [482] | 2010 | US | ✓ |  |  |  | ✓ | ✓ |  |  |  | ✓ |  |  |  |  |  |  |  |  |  | ✓ |  |  |  |  |  |  | ✓ | ✓ |  | 2005 | 2007 | 2 |
| Wong and McMurray [483] | 2002 | Australia |  | ✓ |  |  |  | ✓ |  |  | ✓ |  |  |  |  |  |  |  |  |  |  |  |  |  |  |  |  |  |  | ✓ |  | N/A | N/A | N/A |
| Wood, Patterson, Katikireddi and Hilton [484] | 2014 | UK | ✓ |  |  |  | ✓ |  |  | ✓ |  |  |  |  |  |  |  |  |  |  |  | ✓ |  |  |  |  |  |  | ✓ |  |  | 2005 | 2012 | 7 |
| Wyllie, Baxter and Kulczynski [485] | 2015 | Australia |  | ✓ |  |  |  | ✓ |  |  |  | ✓ |  |  |  |  |  |  | ✓ |  |  |  |  |  |  |  |  |  |  | ✓ |  | N/A | N/A | N/A |
| Yanovitzky and Blitz [486] | 2000 | US | ✓ |  |  |  | ✓ | ✓ |  |  |  |  |  |  | ✓ |  |  |  |  |  |  | ✓ |  |  |  |  |  |  | ✓ | ✓ |  | 1989 | 1991 | 2 |
| Yao, Jiang, Grana, Ling and Glantz [487] | 2016 | China |  | ✓ |  |  | ✓ |  |  |  | ✓ |  |  |  |  |  |  |  |  |  |  |  |  |  |  |  | ✓ |  | ✓ |  |  | 2013 | 2013 | <1 |
| Ye and Ward [488] | 2010 | US |  |  | ✓ |  | ✓ |  |  |  |  |  |  |  |  |  |  |  | ✓ |  | ✓ |  |  |  |  |  |  |  | ✓ |  |  | 2000 | 2007 | 7 |
| Yeh and Jewell [489] | 2015 | US | ✓ |  |  |  |  | ✓ |  |  |  |  |  |  |  |  |  |  | ✓ |  |  |  |  |  |  |  |  | ✓ |  | ✓ |  | N/A | N/A | N/A |
| Yi, Xu, Zhao and Li [490] | 2012 | China | ✓ |  |  |  | ✓ |  |  |  |  |  |  | ✓ |  |  |  |  |  |  | ✓ |  |  |  |  |  |  |  | ✓ |  |  | 1982 | 2009 | 27 |
| Yoo and Kim [491] | 2012 | US |  |  |  | ✓ | ✓ |  |  |  |  |  |  | ✓ |  |  |  |  |  |  |  |  |  |  |  |  | ✓ |  | ✓ |  |  | 2010 | 2010 | <1 |
| Yoon and Lam [492] | 2013 | China |  | ✓ |  |  | ✓ |  |  | ✓ |  |  |  |  |  |  |  |  |  |  |  |  |  |  |  |  | ✓ |  | ✓ |  |  | 2008 | 2012 | 4 |
| Zhao and Pechmann [493] | 2007 | US |  | ✓ |  |  |  | ✓ |  |  | ✓ |  |  |  |  |  |  |  |  |  | ✓ |  |  |  |  |  |  |  |  | ✓ |  | N/A | N/A | N/A |
| Zharekhina and Kubacki [494] | 2015 | Australia |  | ✓ |  |  | ✓ |  |  | ✓ |  |  |  |  |  |  |  |  |  |  | ✓ |  |  |  |  |  | ✓ |  | ✓ |  |  | 2000 | 2013 | 13 |
| Zwarun [495] | 2006 | US |  | ✓ |  |  | ✓ |  |  | ✓ |  |  |  |  |  |  |  |  |  |  | ✓ |  |  |  |  |  |  |  | ✓ |  |  | 2000 | 2002 | 2 |
| Zwier [496] | 2009 | Dutch |  | ✓ |  |  | ✓ |  |  |  |  | ✓ |  |  |  |  |  |  |  |  |  |  | ✓ |  |  |  |  |  | ✓ |  |  | 1990 | 2008 | 18 |

**References**

[1] Abbatangelo-Gray J, Byrd-Bredbenner C, Austin SB. Health and nutrient content claims in food advertisements on Hispanic and mainstream prime-time television. J Nutr Educ Behav. 2008; 40: 348-354.

[2] Abel GA, Lee SJ, Weeks JC. Direct-to-consumer advertising in oncology: a content analysis of print media. J Clin Oncol. 2007; 25: 1267-1271.

[3] Acevedo-Garcia D, Barbeau E, Bishop JA, Pan J, Emmons KM. Undoing an epidemiological paradox: the tobacco industry's targeting of US Immigrants. Am J Public Health. 2004; 94: 2188-2193.

[4] Ackerson LK, Viswanath K. Media attention and public perceptions of cancer and eastern equine encephalitis. J Commun Health. 2010; 35: 409-416.

[5] Akintola O, Lavis JN, Hoskins R. Print media coverage of primary healthcare and related research evidence in South Africa. Health Res Policy Sy. 2015; 13.

[6] Al-Naggar RA, Al-Jashamy K. Breast cancer coverage in the media in malaysia: a qualitative content analysis of star newspaper articles. Asian Pac J Cancer P. 2011; 12: 3397-3401.

[7] Alvy LM, Calvert SL. Food marketing on popular children's web sites: a content analysis. J Am Diet Assoc. 2008; 108: 710-713.

[8] Andersson CM, Bjaras G, Tillgren P, Ostenson C-G. Local media monitoring in process evaluation. experiences from the Stockholm Diabetes Prevention Programme. J Health Commun. 2007; 12: 269-283.

[9] Andsager JL, Chen L, Miles S, Smith CC, Nothwehr F. Nutrition information in community newspapers: goal framing, story origins, and topics. Health Commun. 2015; 30: 1013-1021.

[10] Andsager JL, Powers A. Framing women's health with a sense-making approach: magazine coverage of breast cancer and implants. Health Commun. 2001; 13: 163-185.

[11] Arora R. Message framing and credibility: application in dental services. Health Marketing Quarterly. 2000; 18: 29-44.

[12] Asbeek Brusse ED, Fransen ML, Smit EG. Educational storylines in entertainment television: audience reactions toward persuasive strategies in medical dramas. J Health Commun. 2015; 20: 396-405.

[13] Athanasopoulou C, Sakellari E. Facebook and Health Information: Content Analysis of Groups Related to Schizophrenia. Stud Health Technol Inform. 2015; 213: 255-258.

[14] Atkin CK, Smith SW, McFeters C, Ferguson V. A comprehensive analysis of breast cancer news coverage in leading media outlets focusing on environmental risks and prevention. J Health Commun. 2008; 13: 3-19.

[15] Atkinson AM, Sumnall H, Measham F. Depictions of alcohol use in a UK government partnered online social marketing campaign: Hollyoaks 'The morning after the night before'. Drugs: Education, Prevention & Policy. 2011; 18: 454-467.

[16] Aubrey JS. Looking good versus feeling good: an investigation of media frames of health advice and their effects on women's body-related self-perceptions. Sex Roles. 2010; 63: 50-63.

[17] Aubrey JS, Hahn R. Health versus appearance versus body competence: A content analysis investigating frames of health advice in women's health magazines. J Health Commun. 2016; 21: 496-503.

[18] Austin EW, Pinkleton B, Fujioka Y. Assessing prosocial message effectiveness: effects of message quality, production quality, and persuasiveness. J Health Commun. 1999; 4: 195-210.

[19] Azar D, White V, Bland S, Livingston M, Room R, Chikritzhs T, Durkin S, Gilmore W, Wakefield M. 'Something's brewing': the changing trends in alcohol coverage in Australian newspapers 2000-2011. Alcohol Alcoholism. 2014; 49: 336-342.

[20] Babooram M, Mullan BA, Sharpe L. Children's understandings of mediated health campaigns for childhood obesity. Nutrition & Food Science. 2010; 40: 289-298.

[21] Bach LE, Shelton SC, Moreland-Russell S, Israel K. Smoke-free workplace ballot campaigns: case studies from Missouri and lessons for policy and media advocacy. Am J Health Promot. 2013; 27: e124-133.

[22] Backstrom L. From the freak show to the living room: Cultural representations of dwarfism and obesity. Sociol Forum. 2012; 27: 682-707.

[23] Baek TH, Mayer M. Sexual imagery in cigarette advertising before and after the master settlement agreement. Health Commun. 2010; 25: 747-757.

[24] Balbach ED, Glantz SA. Tobacco information in two grade school newsweeklies: a content analysis. Am J Public Health. 1995; 85: 1650-1653.

[25] Balbach ED, Herzberg A, Barbeau EM. Political coalitions and working women: how the tobacco industry built a relationship with the Coalition of Labor Union Women. Journal of Epidemiology and Community Health. 2006; 60 Suppl 2: 27-32.

[26] Bannon K, Schwartz MB. Impact of nutrition messages on children's food choice: pilot study. Appetite. 2006; 46: 124-129.

[27] Bansal R, John S, Ling PM. Cigarette advertising in Mumbai, India: targeting different socioeconomic groups, women, and youth. Tob Control. 2005; 14: 201-206.

[28] Barker ME, McNeir K, Sameer S, Russell J. Food, nutrition and slimming messages in British women's magazines, 1950-1998. J Hum Nutr Diet. 2014; 27 Suppl 2: 124-134.

[29] Barry CL, Brescoll VL, Gollust SE. Framing childhood obesity: How individualizing the problem affects public support for prevention. Polit Psychol. 2013; 34: 327-349.

[30] Barry CL, Jarlenski M, Grob R, Schlesinger M, Gollust SE. News media framing of childhood obesity in the United States from 2000 to 2009. Pediatrics. 2011; 128: 132-145.

[31] Basil MD, Basil DZ, Schooler C. Cigarette advertising to counter New Year's resolutions. J Health Commun. 2000; 5: 161-174.

[32] Baskin ML, Herbey I, Williams R, Ard JD, Ivankova N, Odoms-Young A. Caregiver perceptions of the food marketing environment of African-American 3-11-year-olds: a qualitative study. Public Health Nutr. 2013; 16: 2231-2239.

[33] Bassett-Gunter RL, Martin Ginis KA, Latimer-Cheung AE. Do you want the good news or the bad news? Gain- versus loss-framed messages following health risk information: the effects on leisure time physical activity beliefs and cognitions. Health Psychol. 2013; 32: 1188-1198.

[34] Beaudoin CE. Exploring antismoking ads: appeals, themes, and consequences. J Health Commun. 2002; 7: 123-137.

[35] Bell K. Science, policy and the rise of 'thirdhand smoke' as a public health issue. Health Risk Soc. 2014; 16: 154-170.

[36] Bell RA, Berger CR, Cassady D, Townsend MS. Portrayals of food practices and exercise behavior in popular American films. J Nutr Educ Behav. 2005; 37: 27-32.

[37] Belstock SA, Connolly GN, Carpenter CM, Tucker L. Using alcohol to sell cigarettes to young adults: a content analysis of cigarette advertisements. J Am Coll Health. 2008; 56: 383-389.

[38] Berenbaum E, Latimer-Cheung AE. Examining the link between framed physical activity ads and behavior among women. J Sport Exerc Psychol. 2014; 36: 271-280.

[39] Bergamini E, Demidenko E, Sargent JD. Trends in tobacco and alcohol brand placements in popular US movies, 1996 through 2009. JAMA Pediatr. 2013; 167: 634-639.

[40] Berry TR, McCarville RE, Rhodes RE. Getting to know the competition: a content analysis of publicly and corporate funded physical activity advertisements. J Health Commun. 2008; 13: 169-180.

[41] Berry TR, Stearns JA, Courneya KS, McGannon KR, Norris CM, Rodgers WM, Spence JC. Women's perceptions of heart disease and breast cancer and the association with media representations of the diseases. J Public Health. 2016; 38: e496-e503.

[42] Berry TR, Wharf-Higgins J, Naylor PJ. SARS wars: an examination of the quantity and construction of health information in the news media. Health Commun. 2007; 21: 35-44.

[43] Beullens K, Schepers A. Display of alcohol use on Facebook: a content analysis. Cyberpsych Beh Soc N. 2013; 16: 497-503.

[44] Bie B, Tang L. Representation of autism in leading newspapers in China: a content analysis. Health Commun. 2015; 30: 884-893.

[45] Bigman CA. Social comparison framing in health news and its effect on perceptions of group risk. Health Commun. 2014; 29: 267-280.

[46] Bissonnette-Maheux V, Provencher V, Lapointe A, Dugrenier M, Dumas A-A, Pluye P, Straus S, Gagnon M-P, Desroches S. Exploring women's beliefs and perceptions about healthy eating blogs: a qualitative study. J Med Internet Res. 2015; 17: e87.

[47] Blake KD, Kaufman AR, Lorenzo J, Augustson EM. A descriptive study of television news coverage of tobacco in the United States: frequency of topics, frames, exemplars, and efficacy. J Health Commun. 2015; 20: 1415-1421.

[48] Bleakley A, Romer D, Jamieson PE. Violent film characters' portrayal of alcohol, sex, and tobacco-related behaviors. Pediatrics. 2014; 133: 71-77.

[49] Boepple L, Ata RN, Rum R, Thompson JK. Strong is the new skinny: a content analysis of fitspiration websites. Body Image. 2016; 17: 132-135.

[50] Boepple L, Thompson JK. A content analysis of healthy living blogs: evidence of content thematically consistent with dysfunctional eating attitudes and behaviors. Int J Eat Disord. 2014; 47: 362-367.

[51] Boessen S, Maarse H. The impact of the treaty basis on health policy legislation in the European Union: a case study on the tobacco advertising directive. BMC Health Serv Res. 2008; 8: 77.

[52] Bonfiglioli C, Hattersley L, King L. Australian print news media coverage of sweet, non-alcoholic drinks sends mixed health messages. Aust NZ J Publ Heal. 2011; 35: 325-330.

[53] Bonfiglioli CMF, Smith BJ, King LA, Chapman SF, Holding SJ. Choice and voice: obesity debates in television news. Med J Australia. 2007; 187: 442-445.

[54] Bonilha Dubugras MT, Evans-Lacko S, de Jesus Mari J. A two-year cross-sectional study on the information about schizophrenia divulged by a prestigious daily newspaper. J Nerv Ment Dis. 2011; 199: 659-665.

[55] Borra ST, Earl R, Hogan EH. Paucity of nutrition and food safety 'news you can use' reveals opportunity for dietetics practitioners. J Am Diet Assoc. 1998; 98: 190-193.

[56] Bosone L, Martinez F, Kalampalikis N. When the model fits the frame: the impact of regulatory fit on efficacy appraisal and persuasion in health communication. Pers Soc Psychol Bull. 2015; 41: 526-539.

[57] Bouman M. Turtles and Peacocks: Collaboration in Entertainment-Education Television. Commun Theory. 2002; 12: 225-244.

[58] Bragg MA, Liu PJ, Roberto CA, Sarda V, Harris JL, Brownell KD. The use of sports references in marketing of food and beverage products in supermarkets. Public Health Nutr. 2013; 16: 738-742.

[59] Brannstrom I, Lindblad I-B. Mass communication and health promotion: The power of the media and public opinion. Health Commun. 1994; 6: 21-36.

[60] Brown-Johnson CG, Sanders-Jackson A, Prochaska JJ. Online comments on smoking bans in psychiatric hospitals units. J Dual Diagn. 2014; 10: 204-211.

[61] Brownson RC, Mack NE, Meegama NI, Pratt M, Brownson CA, Dean C, Dabney S, Luke DA. Changes in newspaper coverage of cardiovascular health issues in conjunction with a community-based intervention. Health Educ Res. 1996; 11: 479-486.

[62] Buis LR, Carpenter S. Health and medical blog content and its relationships with blogger credentials and blog host. Health Commun. 2009; 24: 703-710.

[63] Byrd-Bredbenner C. An internationally shared health frame of reference created by a television program: The Simpsons, a content analysis of health messages. Health Education. 2004; 104: 18-24.

[64] Byrne S, Niederdeppe J, Avery RJ, Cantor J. "When diet and exercise are not enough": an examination of lifestyle change inefficacy claims in direct-to-consumer advertising. Health Commun. 2013; 28: 800-813.

[65] Caburnay CA, Kreuter MW, Cameron G, Luke DA, Cohen E, McDaniels L, Wohlberg M, Atkins P. Black newspapers as a tool for cancer education in African American communities. Ethn Dis. 2008; 18: 488-495.

[66] Cai J, Yang L, Liu Z, Ma Z, Liu Y. Comprehensive analysis of cancer coverage in important Chinese newspapers between 2000 and 2007. Support Care Cancer. 2009; 17: 329-332.

[67] Campo S, Mastin T. Placing the burden on the individual: overweight and obesity in African American and mainstream women's magazines. Health Commun. 2007; 22: 229-240.

[68] Cardador MT, Hazan AR, Glantz SA. Tobacco industry smokers' rights publications: a content analysis. Am J Public Health. 1995; 85: 1212-1217.

[69] Carew C, Kutcher S, Wei Y, McLuckie A. Using digital and social media metrics to develop mental health approaches for youth. Adolescent Psychiat. 2014; 4: 116-121.

[70] Carroll B, Freeman B. 'The secret shame': a content analysis of online news reporting of a celebrity admitting smoking while pregnant. Health Promot J Aust. 2015; 26: 4-9.

[71] Carter SM. From legitimate consumers to public relations pawns: the tobacco industry and young Australians. Tob Control. 2003; 12 Suppl 3: iii71-78.

[72] Cash SJ, Thelwall M, Peck SN, Ferrell JZ, Bridge JA. Adolescent suicide statements on MySpace. Cyberpsych Beh Soc N. 2013; 16: 166-174.

[73] Castonguay J. Portraying physical activity in food advertising targeting children. Health Education. 2015; 115: 534-553.

[74] Castonguay J, McKinley C, Kunkel D. Health-related messages in food advertisements targeting children. Health Education. 2013; 113: 420-432.

[75] Champion D, Chapman S. Framing pub smoking bans: an analysis of Australian print news media coverage, March 1996-March 2003. J Epidemiol Commun Health. 2005; 59: 679-684.

[76] Chan C, Patch C, Williams P. Australian consumers are sceptical about but influenced by claims about fat on food labels. Eur J Clin Nutr. 2005; 59: 148-151.

[77] Chang C. Personal values, advertising, and smoking motivation in Taiwanese adolescents. J Health Commun. 2005; 10: 621-634.

[78] Chang C. Changing smoking attitudes by strengthening weak antismoking beliefs: Taiwan as an example. J Health Commun. 2006; 11: 769-788.

[79] Chang C. Ideal self-image congruency as a motivator for smoking: the moderating effects of personality traits. Health Commun. 2007; 22: 1-12.

[80] Chapman K, Nicholas P, Supramaniam R. How much food advertising is there on Australian television? Health Promot Int. 2006; 21: 172-180.

[81] Chen JY, Eborall H, Armstrong N. Stakeholders' positions in the breast screening debate, and media coverage of the debate: a qualitative study. Crit Public Health. 2014; 24: 62-72.

[82] Choi K, Fabian L, Jansen J, Lenk K, Forster J. Young adults' support for adult-ratings for movies depicting smoking and for restrictions on tobacco magazine advertising. World Medical and Health Policy. 2013; 5: 335-346.

[83] Choi W, Kim H. Health claims for food products advertised on Korean television and their regulation: a content analysis. J Health Commun. 2011; 16: 925-940.

[84] Christenson P, Roberts DF, Bjork N. Booze, drugs, and pop music: trends in substance portrayals in the billboard top 100-1968-2008. Subst Use Misuse. 2012; 47: 121-129.

[85] Chu K-H, Unger JB, Allem J-P, Pattarroyo M, Soto D, Cruz TB, Yang H, Jiang L, Yang CC. Diffusion of messages from an electronic cigarette brand to potential users through Twitter. PLoS ONE. 2015; 10.

[86] Chung JE. Antismoking campaign videos on YouTube and audience response: application of social media assessment metrics. Comput Hum Behav. 2015; 51: 114-121.

[87] Clarke J, van Amerom G. Mass print media depictions of cancer and heart disease: community versus individualistic perspectives? Health Soc Care Community. 2008; 16: 96-103.

[88] Clarke J, van Amerom G, Binns J. Gender and heart disease in mass print media: 1991, 1996, 2001. Women Health. 2007; 45: 17-35.

[89] Clarke JN. Cancer, heart disease, and AIDS: what do the media tell us about these diseases? Health Commun. 1992; 4: 105-120.

[90] Clarke JN. The case of the missing person: Alzheimer's disease in mass print magazines 1991-2001. Health Commun. 2006; 19: 269-276.

[91] Clarke JN, Everest MM. Cancer in the mass print media: fear, uncertainty and the medical model. Soc Sci Med. 2006; 62: 2591-2600.

[92] Clarke JN, Mosleh D. Risk and the Black American child: representations of children's mental health issues in three popular African American magazines. Health Risk Soc. 2015; 17: 1-14.

[93] Cobb NK, Mays D, Graham AL. Sentiment analysis to determine the impact of online messages on smokers' choices to use varenicline. J Natl Cancer I. 2013; 2013: 224-230.

[94] Cohen EL, Caburnay CA, Luke DA, Rodgers S, Cameron GT, Kreuter MW. Cancer coverage in general-audience and Black newspapers. Health Commun. 2008; 23: 427-435.

[95] Cohen EL, Shumate MD, Gold A. Anti-smoking media campaign messages: Theory and practice. Health Commun. 2007; 22: 91-102.

[96] Cokkinides V, Kirkland D, Andrews K, Sullivan K, Lichtenfeld JL. A profile of skin cancer prevention media coverage in 2009. J Am Acad Dermatol 2012; 67: 570-575.

[97] Cole-Lewis H, Perotte A, Galica K, Dreyer L, Griffith C, Schwarz M, Yun C, Patrick H, Coa K, Augustson E. Social network behavior and engagement within a smoking cessation Facebook page. J Med Internet Res. 2016; 18: e205.

[98] Cole-Lewis H, Pugatch J, Sanders A, Varghese A, Posada S, Yun C, Schwarz M, Augustson E. Social listening: a content analysis of e-cigarette discussions on Twitter. J Med Internet Res. 2015; 17: e243.

[99] Cole-Lewis H, Varghese A, Sanders A, Schwarz M, Pugatch J, Augustson E. Assessing electronic cigarette-related tweets for sentiment and content using supervised machine learning. J Med Internet Res. 2015; 17: e208.

[100] Coleman R, Thorson E, Wilkins L. Testing the effect of framing and sourcing in health news stories. J Health Commun. 2011; 16: 941-954.

[101] Commers MJ, Visser G, De Leeuw E. Representations of preconditions for and determinants of health in the Dutch press. Health Promot Int. 2000; 15: 321-332.

[102] Connor SM. Food-related advertising on preschool television: building brand recognition in young viewers. Pediatrics. 2006; 118: 1478-1485.

[103] Cooper CP, Burgoon M, Roter DL. An expectancy-value analysis of viewer interest in television prevention news stories. Health Commun. 2001; 13: 227-240.

[104] Cooper CP, Roter DL, Langlieb AM. Using entertainment television to build a context for prevention news stories. Prev Med. 2000; 31: 225-231.

[105] Cortese DK, Lewis MJ, Ling PM. Tobacco industry lifestyle magazines targeted to young adults. J Adolescent Health. 2009; 45: 268-280.

[106] Covello VT, Peters RG. Women's perceptions of the risks of age-related diseases, including breast cancer: Reports from a 3-year research study. Health Commun. 2002; 14: 377-395.

[107] Cranwell J, Murray R, Lewis S, Leonardi-Bee J, Dockrell M, Britton J. Adolescents' exposure to tobacco and alcohol content in YouTube music videos. Addiction. 2015; 110: 703-711.

[108] Culp J, Bell RA, Cassady D. Characteristics of food industry web sites and "advergames" targeting children. J Nutr Educ Behav. 2010; 42: 197-201.

[109] Dal Cin S, Worth KA, Dalton MA, Sargent JD. Youth exposure to alcohol use and brand appearances in popular contemporary movies. Addiction. 2008; 103: 1925-1932.

[110] Daykin N, Irwin R, Kimberlee R, Orme J, Plant M, McCarron L, Rahbari M. Alcohol, young people and the media: a study of radio output in six radio stations in England. J Public Health. 2009; 31: 105-112.

[111] De Brun A, McCarthy M, McKenzie K, McGloin A. Examining the media portrayal of obesity through the lens of the Common Sense Model of Illness Representations. Health Commun. 2015; 30: 430-440.

[112] De Brun A, McKenzie K, McCarthy M, McGloin A. The emergence and portrayal of obesity in The Irish Times: content analysis of obesity coverage, 1997-2009. Health Commun. 2012; 27: 389-398.

[113] De la Torre-Diez I, Diaz-Pernas FJ, Anton-Rodriguez M. A content analysis of chronic diseases social groups on Facebook and Twitter. Telemed J E Health. 2012; 18: 404-408.

[114] DeJong W. When the tobacco industry controls the news: KKR, RJR Nabisco, and the Weekly Reader Corporation. Tob Control. 1996; 5: 142-148.

[115] DeJong W, Atkin CK. A review of national television PSA campaigns for preventing alcohol-impaired driving, 1987-1992. J Public Health Policy. 1995; 16: 59-80.

[116] DeJong W, Hoffman KD. A content analysis of television advertising for the Massachusetts Tobacco Control Program media campaign, 1993-1996. J Public Health Man. 2000; 6: 27-39.

[117] Dewe M, Ogden J, Coyle A. The cigarette box as an advertising vehicle in the United Kingdom: a case for plain packaging. J Health Psychol. 2015; 20: 954-962.

[118] Dixon H, Warne C, Scully M, Dobbinson S, Wakefield M. Agenda-setting effects of sun-related news coverage on public attitudes and beliefs about tanning and skin cancer. Health Commun. 2014; 29: 173-181.

[119] Dobias KS, Moyer CA, McAchran SE, Katz SJ, Sonnad SS. Mammography messages in popular media: implications for patient expectations and shared clinical decision-making. Health Expect. 2001; 4: 127-135.

[120] Dodgson JE, Tarrant M, Thompson JT, Young B. An analysis of infant feeding content found within the Hong Kong print media. J Hum Lact. 2008; 24: 317-325.

[121] Donaldson EA, Cohen JE, Truant PL, Rutkow L, Kanarek NF, Barry CL. News media framing of New York City's sugar-sweetened beverage portion-size cap. Am J Public Health. 2015; 105: 2202-2209.

[122] Donelle L, Booth RG. Health tweets: an exploration of health promotion on twitter. Online J Issues Nurs. 2012; 17: 4.

[123] Donelle L, Hoffman-Goetz L, Clarke JN. Portrayal of genetic risk for breast cancer in ethnic and non-ethnic newspapers. Women Health. 2004; 40: 93-111.

[124] Donelle L, Hoffman-Goetz L, Clarke JN. Ethnicity, genetics, and breast cancer: media portrayal of disease identities. Ethnic Health. 2005; 10: 185-197.

[125] Doub AE, Small M, Birch L. An exploratory analysis of child feeding beliefs and behaviors included in food blogs written by mothers of preschool-aged children. J Nutr Educ Behav. 2016; 48: 93-103.

[126] Duckworth K, Halpern JH, Schutt RK, Gillespie C. Use of schizophrenia as a metaphor in U.S. newspapers. Psychiatr Serv. 2003; 54: 1402-1404.

[127] Durrant R, Wakefield M, McLeod K, Clegg-Smith K, Chapman S. Tobacco in the news: an analysis of newspaper coverage of tobacco issues in Australia, 2001. Tob Control. 2003; 12 Suppl 2: ii75-81.

[128] Durrant RH, Rome ES, Rich M, Allred E, Emans SJ, Woods ER. Tobacco and alcohol use behaviors portrayed in music videos: a content analysis. Am J Public Health. 1997; 87: 1131-1135.

[129] Earl R, Wellman N. Nutrition news may sidetrack the elderly in their nutrition efforts. J Nutr Elder. 1997; 16: 27-36.

[130] Easson A, Agarwal A, Duda S, Bennett K. Portrayal of Youth Suicide in Canadian News. J Can Acad Child Adolesc Psychiatry. 2014; 23: 167-173.

[131] Eisenberg ME, Carlson-McGuire A, Gollust SE, Neumark-Sztainer D. A content analysis of weight stigmatization in popular television programming for adolescents. Int J Eat Disorder. 2015; 48: 759-766.

[132] Eisenberg ME, Larson NI, Gollust SE, Neumark-Sztainer D. Snacking on television: a content analysis of adolescents' favorite shows. Prev Chronic Dis. 2016; 13: E66.

[133] Elstad EA, Sheridan SL, Lee JGL, Rini C, Earp JA, Brewer NT. Have screening harms become newsworthy? News coverage of prostate and colorectal cancer screening since the 2008 USPSTF recommendation changes. J Behav Med. 2014; 37: 1242-1251.

[134] Emond JA, Smith ME, Mathur SJ, Sargent JD, Gilbert-Diamond D. Children's food and beverage promotion on television to parents. Pediatrics. 2015; 136: 1095-1102.

[135] Eslick GD, Eslick MG. Smoking and The Simpsons. Med J Australia. 2009; 190: 637-639.

[136] Eyal K, Te'eni-Harari T. High on attractiveness, low on nutrition: An over-time comparison of advertising food products on Israeli television. Health Commun. 2016; 31: 988-997.

[137] Fagan J, Lyons S, Smyth BP. Content analysis of newspaper reports on alcohol-related deaths. Alcohol Alcoholism. 2014; 49: 479-483.

[138] Faulkner G, Finlay S-J, Roy SC. Get the news on physical activity research: a content analysis of physical activity research in the Canadian print media. J Phys Act Health. 2007; 4: 180-192.

[139] Fishman J, Ten Have T, Casarett D. Cancer and the media: how does the news report on treatment and outcomes? Arch Intern Med. 2010; 170: 515-518.

[140] Fishman JM, Ten Have T, Casarett D. Is public communication about end-of-life care helping to inform all? Cancer news coverage in African American versus mainstream media. Cancer. 2012; 118: 2157-2162.

[141] Fogarty AS, Chapman S. Framing and the marginalisation of evidence in media reportage of policy debate about alcopops, Australia 2008-2009: implications for advocacy. Drug Alcohol Rev. 2011; 30: 569-576.

[142] Fogarty AS, Chapman S. Advocates, interest groups and Australian news coverage of alcohol advertising restrictions: content and framing analysis. BMC Public Health. 2012; 12: 727.

[143] Fogarty AS, Chapman S. Australian television news coverage of alcohol, health and related policies, 2005 to 2010: implications for alcohol policy advocates. Aust NZ J Publ Heal. 2012; 36: 530-536.

[144] Foley NM, O'Mahony M, Lehane EA, Cil T, Corrigan MA. A qualitative content analysis of breast cancer narratives hosted through the medium of social media. Br J Med Med Res. 2015; 6: 474-483.

[145] Folta SC, Goldberg JP, Economos C, Bell R, Meltzer R. Food advertising targeted at school-age children: a content analysis. J Nutr Educ Behav. 2006; 38: 244-248.

[146] Foss KA, Southwell BG. Infant feeding and the media: the relationship between Parents' Magazine content and breastfeeding, 1972-2000. Int Breastfeed J. 2006; 1.

[147] Fosu I, Wicks JL, Warren R, Wicks RH. What's on the menu? Disclaimers, emotional appeals and production techniques in food advertising on child-rated programs in the United States. Journal of Children and Media. 2013; 7: 334-348.

[148] Franko DL, Rodgers RF, Lovering M, Fernandes C, Alfieri A, Matsumoto A, Accomando K, Thompson-Brenner H. Time trends in cover images and article content in Latina magazine: potential implications for body dissatisfaction in Latina women. Journal of Latina/o Psychology. 2013; 1: 243-254.

[149] Frederick DA, Saguy AC, Sandhu G, Mann T. Effects of competing news media frames of weight on antifat stigma, beliefs about weight and support for obesity-related public policies. Int J Obes. 2016; 40: 543-549.

[150] Freeman B. Tobacco plain packaging legislation: a content analysis of commentary posted on Australian online news. Tob Control. 2011; 20: 361-366.

[151] Freeman B, Chapman S, Storey P. Banning smoking in cars carrying children: an analytical history of a public health advocacy campaign. Aust NZ J Publ Heal. 2008; 32: 60-65.

[152] Frerichs L, Andsager JL, Campo S, Aquilino M, Dyer CS. Framing Breastfeeding and Formula-Feeding Messages in Popular U.S. Magazines. Women Health. 2006; 44: 95-118.

[153] Friedman DB, Laditka SB, Laditka JN, Price AE. A content analysis of cognitive health promotion in popular magazines. Int J Aging Hum Dev. 2011; 73: 253-281.

[154] Fu KW, Yip PSF. Changes in reporting of suicide news after the promotion of the WHO media recommendations. Suicide Life Threat Behav. 2008; 38: 631-636.

[155] Galcheva SV, Iotova VM, Stratev VK. Television food advertising directed towards Bulgarian children. Arch Dis Child. 2008; 93: 857-861.

[156] Gantz W, Wang Z. Coverage of cancer in local television news. J Cancer Educ. 2009; 24: 65-72.

[157] Gase LN, Montes C, Robles B, Tyree R, Kuo T. Media outlet and consumer reactions to promotional activities of the Choose Health LA Restaurants program in Los Angeles County. J Public Health Man. 2016; 22: 231-244.

[158] Gearhart S, Craig C, Steed C. Network news coverage of obesity in two time periods: an analysis of issues, sources, and frames. Health Commun. 2012; 27: 653-662.

[159] Geyzen A. Popular discourse on nutrition, health and indulgence in Flanders, 1945-1960. Appetite. 2011; 56: 278-283.

[160] Ghaznavi J, Taylor LD. Bones, body parts, and sex appeal: an analysis of #thinspiration images on popular social media. Body Image. 2015; 14: 54-61.

[161] Godbold Kean L, Prividera LC. Communicating about race and health: a content analysis of print advertisements in African American and general readership magazines. Health Commun. 2007; 21: 289-297.

[162] Gold AL, Cohen EL, Shumate M. Proscriptive models and evidence in antismoking advertising. Health Commun. 2008; 23: 259-269.

[163] Gollust SE, Lantz PM. Communicating population health: print news media coverage of type 2 diabetes. Soc Sci Med. 2009; 69: 1091-1098.

[164] Gollust SE, Lantz PM, Ubel PA. The polarizing effect of news media messages about the social determinants of health. Am J Public Health. 2009; 99: 2160-2167.

[165] Gould MS, Kleinman MH, Lake AM, Forman J, Midle JB. Newspaper coverage of suicide and initiation of suicide clusters in teenagers in the USA, 1988&#x2013;96: a retrospective, population-based, case-control study. Lancet Psychiatry. 1: 34-43.

[166] Goulden R, Corker E, Evans-Lacko S, Rose D, Thornicroft G, Henderson C. Newspaper coverage of mental illness in the UK, 1992-2008. BMC Public Health. 2011; 11: 796.

[167] Graham AL, Fang Y, Moreno JL, Streiff SL, Villegas J, Munoz RF, Tercyak KP, Mandelblatt JS, Vallone DM. Online advertising to reach and recruit Latino smokers to an internet cessation program: impact and costs. J Med Internet Res. 2012; 14: e116.

[168] Grana RA, Ling PM. "Smoking revolution": a content analysis of electronic cigarette retail websites. Am J Prev Med. 2014; 46: 395-403.

[169] Granner ML, Sharpe PA, Burroughs EL, Fields R, Hallenbeck J. Newspaper content analysis in evaluation of a community-based participatory project to increase physical activity. Health Educ Res. 2010; 25: 656-667.

[170] Greenberg M, Wartenberg D. Understanding mass media coverage of disease clusters. A J Epidemiol. 1990; 132: S192-195.

[171] Gruber EL, Thau HM, Hill DL, Fisher DA, Grube JW. Alcohol, tobacco and illicit substances in music videos: a content analysis of prevalence and genre. J Adolescent Health. 2005; 37: 81-83.

[172] Guenther L, Froehlich K, Milde J, Heidecke G, Ruhrmann G. Effects of valenced media frames of cancer diagnoses and therapies: quantifying the transformation and establishing of evaluative schemas. Health Commun. 2015; 30: 1055-1064.

[173] Gugsa F, Karmarkar E, Cheyne A, Yamey G. Newspaper coverage of maternal health in Bangladesh, Rwanda and South Africa: a quantitative and qualitative content analysis. BMJ Open. 2016; 6: e008837.

[174] Gunderson MD, Clements D, Benjamin Neelon SE. Nutritional quality of foods marketed to children in Honduras. Appetite. 2014; 73: 1-6.

[175] Guran T, Turan S, Akcay T, Degirmenci F, Avci O, Asan A, Erdil E, Majid A, Bereket A. Content analysis of food advertising in Turkish television. J Paediatr Child. 2010; 46: 427-430.

[176] Haddock CK, Hoffman K, Taylor JE, Schwab L, Poston WSC, Lando HA. An analysis of messages about tobacco in the Military Times magazines. Nicotine Tob Res. 2008; 10: 1191-1197.

[177] Haddock CK, Parker LC, Taylor JE, Poston WSC, Lando H, Talcott GW. An analysis of messages about tobacco in military installation newspapers. Am J Public Health. 2005; 95: 1458-1463.

[178] Hale TM, Pathipati AS, Zan S, Jethwani K. Representation of health conditions on Facebook: content analysis and evaluation of user engagement. J Med Internet Res. 2014; 16: 18-33.

[179] Hamad R, Pomeranz JL, Siddiqi A, Basu S. Large-scale automated analysis of news media: a novel computational method for obesity policy research. Obesity. 2015; 23: 296-300.

[180] Hampl JS, Wharton CM, Taylor CA, Winham DM, Block JL, Hall R. Primetime television impacts on adolescents' impressions of bodyweight, sex appeal, and food and beverage consumption. Nutr Bull. 2004; 29: 92-98.

[181] Hanewinkel R, Wiborg G. Smoking in contemporary German television programming. Int J Public Health. 2007; 52: 308-312.

[182] Hanewinkel R, Wiborg G. Smoking in a popular German television crime series 1985-2004. Prev Med. 2008; 46: 596-598.

[183] Harris JK, Moreland-Russell S, Choucair B, Mansour R, Staub M, Simmons K. Tweeting for and against public health policy: response to the Chicago Department of Public Health's electronic cigarette Twitter campaign. J Med Internet Res. 2014; 16: e238.

[184] Harris JK, Mueller NL, Snider D, Haire-Joshu D. Local health department use of twitter to disseminate diabetes information, United States. Prev Chronic Dis. 2013; 10: E70.

[185] Harris JL, LoDolce M, Dembek C, Schwartz MB. Sweet promises: candy advertising to children and implications for industry self-regulation. Appetite. 2015; 95: 585-590.

[186] Hartman MA, Nierkens V, Cremer SW, Verhoeff A, Stronks K. Is channel segmentation necessary to reach a multiethnic population with weight-related health promotion? An analysis of use and perception of communication channels. Ethnic Health. 2015; 20: 194-208.

[187] Harwood EM, Witson JC, Fan DP, Wagenaar AC. Media advocacy and underage drinking policies: a study of Louisiana news media from 1994 through 2003. Health Promot Pract. 2005; 6: 246-257.

[188] Hawkins KW, Linvill DL. Public health framing of news regarding childhood obesity in the United States. Health Commun. 2010; 25: 709-717.

[189] Hayes M, Ross IE, Gasher M, Gutstein D, Dunn JR, Hackett RA. Telling stories: news media, health literacy and public policy in Canada. Soc Sci Med. 2007; 64: 1842-1852.

[190] He S, Shen Q, Yin X, Xu L, Lan X. Newspaper coverage of tobacco issues: an analysis of print news in Chinese cities, 2008-2011. Tob Control. 2014; 23: 345-352.

[191] Hellman M, Karlsson T. In fear of a reversal back to the spirits-drinking era-The 2004 decrease of Finnish alcohol taxes in public discourse. Nord Stud Alcohol Dr. 2012; 29: 57-77.

[192] Hellyer NE, Haddock-Fraser J. Reporting diet-related health issues through newspapers: portrayal of cardiovascular disease and Type 2 diabetes. Health Educ Res. 2011; 26: 13-25.

[193] Henderson J, Coveney J, Ward P, Taylor A. Governing childhood obesity: framing regulation of fast food advertising in the Australian print media. Soc Sci Med. 2009; 69: 1402-1408.

[194] Henderson L, Kitzinger J, Green J. Representing infant feeding: content analysis of British media portrayals of bottle feeding and breast feeding. BMJ. 2000; 321: 1196-1198.

[195] Henderson VR, Kelly B. Food advertising in the age of obesity: content analysis of food advertising on general market and african american television. J Nutr Educ Behav. 2005; 37: 191-196.

[196] Heneghan MK, Hazan C, Halpern AC, Oliveria SA. Skin cancer coverage in a national newspaper: a teachable moment. J Cancer Educ. 2007; 22: 99-104.

[197] Heuer CA, McClure KJ, Puhl RM. Obesity stigma in online news: a visual content analysis. J Health Commun. 2011; 16: 976-987.

[198] Higgins JW, Naylor PJ, Berry T, O'Connor B, McLean D. The health buck stops where? Thematic framing of health discourse to understand the context for CVD prevention. J Health Commun. 2006; 11: 343-358.

[199] Hilbert A, Ried J. Obesity in print: an analysis of daily newspapers. Obes Facts. 2009; 2: 46-51.

[200] Hill JM, Radimer KL. Health and nutrition messages in food advertisements: A comparative content analysis of young and mature Australian women's magazines. J Nutr Educ. 1996; 28: 313-320.

[201] Hilton S, Hunt K. Coverage of Jade Goody's cervical cancer in UK newspapers: a missed opportunity for health promotion? BMC Public Health. 2010; 10.

[202] Hilton S, Patterson C, Teyhan A. Escalating coverage of obesity in UK newspapers: the evolution and framing of the "obesity epidemic" from 1996 to 2010. Obesity. 2012; 20: 1688-1695.

[203] Hilton S, Wood K, Bain J, Patterson C, Duffy S, Semple S. Newsprint coverage of smoking in cars carrying children: a case study of public and scientific opinion driving the policy debate. BMC Public Health. 2014; 14: 1116.

[204] Hilton S, Wood K, Patterson C, Katikireddi SV. Implications for alcohol minimum unit pricing advocacy: what can we learn for public health from UK newsprint coverage of key claim-makers in the policy debate? Soc Sci Med. 2014; 102: 157-164.

[205] Himes SM, Thompson JK. Fat stigmatization in television shows and movies: A content analysis. Obesity. 2007; 15: 712-718.

[206] Hinnant A, Oh H, Caburnay CA, Kreuter MW. What makes African American health disparities newsworthy? An experiment among journalists about story framing. Health Educ Res. 2011; 26: 937-947.

[207] Hoffman-Goetz L, Shannon C, Clarke JN. Chronic disease coverage in Canadian Aboriginal newspapers. J Health Commun. 2003; 8: 475-488.

[208] Hoffner C, Ye J. Young adults' responses to news about sunscreen and skin cancer: the role of framing and social comparison. Health Commun. 2009; 24: 189-198.

[209] Holland K, Blood RW, Thomas SL, Lewis S. Challenging stereotypes and legitimating fat: an analysis of obese people's views on news media reporting guidelines and promoting body diversity. J Sociol. 2015; 51: 431-445.

[210] Holland KE, Blood RW, Thomas SI, Lewis S, Komesaroff PA, Castle DJ. 'Our girth is plain to see': An analysis of newspaper coverage of Australia's Future 'Fat Bomb'. Health Risk Soc. 2011; 13: 31-46.

[211] Holmes-Rovner M, Charles S. The mammography screening controversy: who and what is heard in the press? Patient Educ Couns. 2003; 51: 75-81.

[212] Holmes BJ. Media coverage of Canada's obesity epidemic: illustrating the subtleties of surveillance medicine. Crit Public Health. 2009; 19: 223-233.

[213] Holton A, Lee N, Coleman R. Commenting on health: A framing analysis of user comments in response to health articles online. J Health Commun. 2014; 19: 825-837.

[214] Huang L, Mehta K, Wong M. Television food advertising in Singapore: the nature and extent of children's exposure. Health Promot Int. 2012; 27: 187-196.

[215] Hubbell AP, Dearing JW. Local newspapers, community partnerships, and health improvement projects: their roles in a comprehensive community initiative. J Commun Health. 2003; 28: 363-376.

[216] Hurley RJ, Kosenko KA, Brashers D. Uncertain terms: message features of online cancer news. Commun Monogr. 2011; 78: 370-390.

[217] Hurley RJ, Riles JM, Sangalang A. Online cancer news: trends regarding article types, specific cancers, and the cancer continuum. Health Commun. 2014; 29: 41-50.

[218] Jahns L, Payne CR, Whigham LD, Johnson LK, Scheett AJ, Hoverson BS, Kranz S. Foods advertised in US weekly supermarket sales circulars over one year: a content analysis. Nutr J. 2014; 13: 95.

[219] Jarlenski M, Barry CL. News media coverage of trans fat: health risks and policy responses. Health Commun. 2013; 28: 209-216.

[220] Jawad M, Bakir AM, Ali M, Jawad S, Akl EA. Key health themes and reporting of numerical cigarette-waterpipe equivalence in online news articles reporting on waterpipe tobacco smoking: a content analysis. Tob Control. 2015; 24: 43-47.

[221] Jenkin GL, Signal L, Thomson G. Framing obesity: the framing contest between industry and public health at the New Zealand inquiry into obesity. Obes Rev. 2011; 12: 1022-1030.

[222] Jenssen BP, Klein JD, Salazar LF, Daluga NA, DiClemente RJ. Exposure to tobacco on the internet: content analysis of adolescents' internet use. Pediatrics. 2009; 124: e180-186.

[223] Jeong M, Gilmore JS, Bleakley A, Jordan A. Local news media framing of obesity in the context of a sugar-sweetened beverage reduction media campaign. J Nutr Educ Behav. 2014; 46: 583-588.

[224] Jernigan DH, Wright PA. Media advocacy: lessons from community experiences. J Public Health Policy. 1996; 17: 306-330.

[225] Johnson CA, Johnson BE. Medicine on British television: a content analysis. J Commun Health. 1993; 18: 25-35.

[226] Jones-Webb R, Baranowski S, Fan D, Finnegan J, Wagenaar AC. Content analysis of coverage of alcohol control policy issues in black-oriented and mainstream newspapers in the U.S. J Public Health Policy. 1997; 18: 49-66.

[227] Jones K, Merrick J, Beasley C. A content analysis of oral health messages in Australian mass media. Aust Dent J. 2016; 61: 16-20.

[228] Kaczynski AT, Havitz ME, McCarville RE. Altering perceptions through repositioning: an exercise in framing. Leisure Sciences. 2005; 27: 241-261.

[229] Kalin SR, Fung TT. Comparison of child obesity prevention and control content in mainstream and Spanish-language US parenting magazines. J Acad Nutr Diet. 2013; 113: 133-140.

[230] Kang J, Lin CA. Effects of message framing and visual-fear appeals on smoker responses to antismoking ads. J Health Commun. 2015; 20: 647-655.

[231] Kang S, Gearhart S, Bae H-S. Coverage of Alzheimer's disease from 1984 to 2008 in television news and information talk shows in the United States: an analysis of news framing. Am J Alzheimers Dis. 2010; 25: 687-697.

[232] Karupaiah T, Chinna K, Mee LH, Mei LS, Noor MI. What's on Malaysian television? A survey on food advertising targeting children. Asia Pac J Clin Nutr. 2008; 17: 483-491.

[233] Kees J. Advertising framing effects and consideration of future consequences. J Consum Aff. 2011; 45: 7-32.

[234] Keller SK, Schulz PJ. Distorted food pyramid in kids programmes: a content analysis of television advertising watched in Switzerland. Eur J Public Health. 2011; 21: 300-305.

[235] Kelly B, Chapman K. Food references and marketing to children in Australian magazines: a content analysis. Health Promot Int. 2007; 22: 284-291.

[236] Kennedy GE, Bero LA. Print media coverage of research on passive smoking. Tob Control. 1999; 8: 254-260.

[237] Kent MP, Dubois L, Kent EA, Wanless AJ. Internet marketing directed at children on food and restaurant websites in two policy environments. Obesity. 2013; 21: 800-807.

[238] Kent MP, Dubois L, Wanless A. Food marketing on children's television in two different policy environments. Int J Pediatr Obes. 2011; 6: e433-441.

[239] Kerr RL. A beer a minute in Texas football: Heavy drinking and the heroizing of the antihero in Friday Night Lights. Int Rev Sociol Sport. 2014; 49: 451-467.

[240] Kesten JM, Cohn S, Ogilvie D. The contribution of media analysis to the evaluation of environmental interventions: the commuting and health in Cambridge study. BMC Public Health. 2014; 14: 482.

[241] Kim AE, Kumanyika S, Shive D, Igweatu U, Kim SH. Coverage and framing of racial and ethnic health disparities in US newspapers, 1996-2005. Am J Public Health. 2010; 100: S224-S231.

[242] Kim E, Hou J, Han JY, Himelboim I. Predicting retweeting behavior on breast cancer social networks: network and content characteristics. J Health Commun. 2016; 21: 479-486.

[243] Kim K, Paek H-J, Lynn J. A content analysis of smoking fetish videos on YouTube: regulatory implications for tobacco control. Health Commun. 2010; 25: 97-106.

[244] Kim M, Lennon SJ. Content Analysis of diet advertisements: a cross-national comparison of Korean and U.S. women's magazines. Clothing & Textiles Research Journal. 2006; 24: 345-362.

[245] Kim SH, Willis LA. Talking about obesity: news framing of who is responsible for causing and fixing the problem. J Health Commun. 2007; 12: 359-376.

[246] Kim SJ, Niederdeppe J. Emotional expressions in antismoking television advertisements: consequences of anger and sadness framing on pathways to persuasion. J Health Commun. 2014; 19: 692-709.

[247] Klos LA, Greenleaf C, Paly N, Kessler MM, Shoemaker CG, Suchla EA. Losing weight on reality TV: a content analysis of the weight loss behaviors and practices portrayed on the biggest loser. J Health Commun. 2015; 20: 639-646.

[248] Knox E, Biddle S, Esliger DW, Piggin J, Sherar L. Accounting for sitting and moving: An analysis of sedentary behavior in mass media campaigns. J Phys Act Health. 2015; 12: 1198-1204.

[249] Konfortion J, Jack RH, Davies EA. Coverage of common cancer types in UK national newspapers: a content analysis. BMJ Open. 2014; 4: e004677.

[250] Kostygina G, Hahn EJ, Rayens MK. 'It's about the smoke, not the smoker': messages that motivate rural communities to support smoke-free policies. Health Educ Res. 2014; 29: 58-71.

[251] Krauss MJ, Sowles SJ, Moreno M, Zewdie K, Grucza RA, Bierut LJ, Cavazos-Rehg PA. Hookah-related Twitter chatter: a content analysis. Prev Chronic Dis. 2015; 12: E121.

[252] Krishen AS, Bui M. Fear advertisements: Influencing consumers to make better health decisions. Int J Advert. 2015; 34: 533-548.

[253] Kromm EE, Smith KC, Singer RF. Survivors on cancer: the portrayal of survivors in print news. J Cancer Surviv. 2007; 1: 298-305.

[254] Kuiper NM, Frantz KE, Cotant M, Babb S, Jordan J, Phelan M. Newspaper coverage of implementation of the Michigan smoke-free law: lessons learned. Health Promot Pract. 2013; 14: 901-908.

[255] Kunkel DL, Castonguay JS, Filer CR. Evaluating industry self-regulation of food marketing to children. Am J Prev Med. 2015; 49: 181-187.

[256] Kurko T, Linden K, Kolstela M, Pietila K, Airaksinen M. Is nicotine replacement therapy overvalued in smoking cessation? Analysis of smokers' and quitters' communication in social media. Health Expect. 2015; 18: 2962-2977.

[257] Kye SY, Kwon JH, Kim Y-C, Shim M, Kim JH, Cho H, Jung KW, Park K. Cancer risk factors in Korean news media: a content analysis. Asian Pac J Cancer Prev. 2015; 16: 731-736.

[258] Lando HA, Michaud ME, Poston WSC, Jahnke SA, Williams L, Haddock CK. Banning cigarette smoking on US Navy submarines: a case study. Tob Control. 2015; 24: e188-e192.

[259] Lapinski MK. StarvingforPerfect.com: a theoretically based content analysis of pro-eating disorder Web sites. Health Commun. 2006; 20: 243-253.

[260] Larson S, Long M, Slater MD, Bettinghaus EP, Read A. A content analysis of cancer survivorship coverage in a representative sample of US news outlets. J Cancer Educ. 2009; 24: 291-296.

[261] Lascu D-N, Manrai AK, Manrai LA, Amissah FB. Online marketing of food products to children: the effects of national consumer policies in high-income countries. Young Consumers. 2013; 14: 19-40.

[262] Lavack AM. Message content of alcohol moderation TV commercials: impact of corporate versus nonprofit sponsorship. Health Marketing Quarterly. 1999; 16: 15-31.

[263] Lawhon M, Herrick C. Alcohol control in the news: the politics of media representations of alcohol policy in South Africa. J Health Polit Policy Law. 2013; 38: 987-1021.

[264] Lee C-J, Long M, Slater MD, Song W. Comparing local TV news with national TV news in cancer coverage: an exploratory content analysis. J Health Commun. 2014; 19: 1330-1342.

[265] Lee H, Lee Y, Park S-A, Willis E, Cameron GT. What are Americans seeing? Examining the message frames of local television health news stories. Health Commun. 2013; 28: 846-852.

[266] Lee H, Len-Rios ME. Defining obesity: second-level agenda setting attributes in black newspapers and general audience newspapers. J Health Commun. 2014; 19: 1116-1129.

[267] Lee JGL, Agnew-Brune CB, Clapp JA, Blosnich JR. Out smoking on the big screen: tobacco use in LGBT movies, 2000-2011. Tob Control. 2014; 23: e156-158.

[268] Lee M, Choi Y, Quilliam ET, Cole RT. Playing with food: Content analysis of food advergames. J Consum Aff. 2009; 43: 129-154.

[269] Lee MJ, Gispanski L. Portrayals of eating and drinking in popular American TV programs: a comparison of scripted and unscripted shows. J Health Commun. 2016; 21: 593-599.

[270] Lee S, Cheng I. Assessing the TARES as an ethical model for antismoking ads. J Health Commun. 2010; 15: 55-75.

[271] Lemal M, Custers K, Bulck Jvd. The portrayal of health problems in entertainment television: implications for risk perception and health promotion. Int J Public Health. 2010; 3: 77-86.

[272] Lemmens PH, Vaeth PA, Greenfield TK. Coverage of beverage alcohol issues in the print media in the United States, 1985-1991. Am J Public Health. 1999; 89: 1555-1560.

[273] Leshner G, Bolls P, Wise K. Motivated processing of fear appeal and disgust images in televised anti-tobacco ads. Journal of Media Psychology: Theories, Methods, and Applications. 2011; 23: 77-89.

[274] Leshner G, Cheng IH. The effects of frame, appeal, and outcome extremity of antismoking messages on cognitive processing. Health Commun. 2009; 24: 219-227.

[275] Lewis MK, Hill AJ. Food advertising on British children's television: a content analysis and experimental study with nine-year olds. Int J Obes. 1998; 22: 206-214.

[276] Lima JC, Siegel M. The tobacco settlement: an analysis of newspaper coverage of a national policy debate, 1997-98. Tob Control. 1999; 8: 247-253.

[277] Lindsay S, Thomas S, Lewis S, Westberg K, Moodie R, Jones S. Eat, drink and gamble: marketing messages about 'risky' products in an Australian major sporting series. BMC Public Health. 2013; 13: 719.

[278] Lingas EO. Promoting health: media advocacy opportunities in English- and Spanish-language masthead editorials. Am J Health Promot. 2013; 27: 200-204.

[279] Liu Y, Liu M, Xiao H, Cai J, Xu W. A content analysis of news coverage of skin cancer in China newspapers. Health Commun. 2010; 25: 647-649.

[280] LoDolce ME, Harris JL, Schwartz MB. Sugar as part of a balanced breakfast? What cereal advertisements teach children about healthy eating. J Health Commun. 2013; 18: 1293-1309.

[281] Long M, Slater MD, Lysengen L. US news media coverage of tobacco control issues. Tob Control. 2006; 15: 367-372.

[282] Love LA, Sterns JA, Spreen TH, Wysocki AF. When consumers diet, should producers care? An examination of low-carb dieting and U.S. orange juice consumption. Journal of Food Distribution Research. 2006; 37: 68-76.

[283] Ludwick R, Rushing B, Biordi DL. Breast cancer and the older woman: information and images. Health Care Women In. 1994; 15: 235-242.

[284] Ma GX, Fleisher L, Gonzalez E, Edwards RL. Improving cancer awareness among Asian Americans using targeted and culturally appropriate media: a case study. Home Health Care Manag Pract. 2004; 17: 39-44.

[285] MacKenzie R, Chapman S, Holding S. Framing responsibility: coverage of lung cancer among smokers and non-smokers in Australian television news. Aust NZ J Publ Heal. 2011; 35: 66-70.

[286] MacKenzie R, Chapman S, Holding S, McGeechan K. 'A matter of faith, not science': analysis of media coverage of prostate cancer screening in Australian news media 2003-2006. J R Soc Med. 2007; 100: 513-521.

[287] MacKenzie R, Chapman S, Holding S, Stiven A. "No respecter of youth": Over-representation of young women in Australian television coverage of breast cancer. J Cancer Educ. 2010; 25: 565-570.

[288] Mackenzie R, Chapman S, Johnson N, McGeechan K, Holding S. The newsworthiness of cancer in Australian television news. Med J Australia. 2008; 189: 155-158.

[289] MacKenzie R, Imison M, Chapman S, Holding S. Mixed messages and a missed opportunity: Australian news media coverage of Clare Oliver's campaign against solaria. Med J Australia. 2008; 189: 371-374.

[290] Mackenzie R, Johnson N, Chapman S, Holding S. Smoking-related disease on Australian television news: inaccurate portrayals may contribute to public misconceptions. Aust NZ J Publ Heal. 2009; 33: 144-146.

[291] MacLean A, Sweeting H, Walker L, Patterson C, Raisanen U, Hunt K. "It's not healthy and it's decidedly not masculine": a media analysis of UK newspaper representations of eating disorders in males. BMJ Open. 2015; 5: e007468.

[292] Magzamen S, Charlesworth A, Glantz SA. Print media coverage of California's smokefree bar law. Tob Control. 2001; 10: 154.

[293] Maher A, Wilson N, Signal L. Advertising and availability of 'obesogenic' foods around New Zealand secondary schools: a pilot study. N Z Med J. 2005; 118: U1556.

[294] Mainland M, Shaw S, Prier A. Fearing fat: exploring the discursive links between childhood obesity, parenting, and leisure. J Leisure Res. 2015; 47: 202-219.

[295] Major LH. Break it to me harshly: the effects of intersecting news frames in lung cancer and obesity coverage. J Health Commun. 2009; 14: 174-188.

[296] Malone RE, Wenger LD, Bero LA. High school journalists' perspectives on tobacco. J Health Commun. 2002; 7: 139-156.

[297] Manganello JA, Clegg Smith K, Sudakow K, Summers AC. A content analysis of food advertisements appearing in parenting magazines. Public Health Nutr. 2013; 16: 2188-2196.

[298] Mannien J, van den Brandhof WE, McIntyre E, Hiller JE. Breastfeeding articles in the Australian press: 1996-1999. Breastfeeding Review. 2002; 10: 5-10.

[299] Martin-Biggers J, Yorkin M, Aljallad C, Ciecierski C, Akhabue I, McKinley J, Hernandez K, Yablonsky C, Jackson R, Quick V *et al*. What foods are US supermarkets promoting? A content analysis of supermarket sales circulars. Appetite. 2013; 62: 160-165.

[300] Martinez R, Johnston-Robledo I, Ulsh HM, Chrisler JC. Singing "the baby blues": a content analysis of popular press articles about postpartum affective disturbances. Women Health. 2000; 31: 37-56.

[301] Mastin T, Campo S. Conflicting Messages: Overweight and Obesity Advertisements and Articles in Black Magazines. Howard Journal of Communications. 2006; 17: 265-285.

[302] McCauley MP, Blake KD, Meissner HI, Viswanath K. The social group influences of US health journalists and their impact on the newsmaking process. Health Educ Res. 2013; 28: 339-351.

[303] McCaw BA, McGlade KJ, McElnay JC. Online health information - what the newspapers tell their readers: a systematic content analysis. BMC Public Health. 2014; 14: 1316.

[304] McDaniel PA, Offen N, Yerger V, Forsyth S, Malone RE. "Tired of watching customers walk out the door because of the smoke": a content analysis of media coverage of voluntarily smokefree restaurants and bars. BMC Public Health. 2015; 15: 761.

[305] McGee R, Bang S, Marsh L. Newspaper coverage of tobacco control in New Zealand. Aust NZ J Publ Heal. 2014; 38: 265-269.

[306] McGee R, Ketchel J. Tobacco imagery on New Zealand television 2002-2004. Tob Control. 2006; 15: 412-414.

[307] McGee R, Ketchel J, Reeder AI. Alcohol imagery on New Zealand television. Subst Abuse Treat Prev Policy. 2007; 2: 6.

[308] McHiza ZJ, Temple NJ, Steyn NP, Abrahams Z, Clayford M. Content analysis of television food advertisements aimed at adults and children in South Africa. Public Health Nutr. 2013; 16: 2213-2220.

[309] McIntosh WA. The symbolization of eggs in American culture: a sociologic analysis. J Am Coll Nutr. 2000; 19: 532S-539S.

[310] McLeod K, Wakefield M, Chapman S, Smith KC, Durkin S. Changes in the news representation of smokers and tobacco-related media advocacy from 1995 to 2005 in Australia. Journal of Epidemiology and Community Health. 2009; 63: 215-220.

[311] McWhirter JE, Hoffman-Goetz L. Coverage of skin cancer and recreational tanning in North American magazines before and after the landmark 2006 International Agency for Research on Cancer report. BMC Public Health. 2015; 15.

[312] McWhirter JE, Hoffman-Goetz L. North American magazine coverage of skin cancer and recreational tanning before and after the WHO/IARC 2009 classification of indoor tanning devices as carcinogenic. J Cancer Educ. 2015; 30: 477-481.

[313] McWhirter JE, Hoffman-Goetz L. Skin deep: coverage of skin cancer and recreational tanning in Canadian women's magazines (2000-2012). Can J Public Health. 2015; 106: e236-e243.

[314] McWhirter JE, Hoffman-Goetz L. Coverage of skin cancer risk factors and UV behaviors in popular U.S. magazines from 2000 to 2012. J Cancer Educ. 2016; 31: 382-388.

[315] McWhirter JE, Hoffman-Goetz L, Clarke JN. Can you see what they are saying? Breast cancer images and text in Canadian women's and fashion magazines. J Cancer Educ. 2012; 27: 383-391.

[316] Mejia P, Dorfman L, Cheyne A, Nixon L, Friedman L, Gottlieb M, Daynard R. The origins of personal responsibility rhetoric in news coverage of the tobacco industry. Am J Public Health. 2014; 104: 1048-1051.

[317] Mekemson C, Glik D, Titus K, Myerson A, Shaivitz A, Ang A, Mitchell S. Tobacco use in popular movies during the past decade. Tob Control. 2004; 13: 400-402.

[318] Menashe CL, Siegel M. The power of a frame: an analysis of newspaper coverage of tobacco issues: United States, 1985-1996. J Health Commun. 1998; 3: 307-325.

[319] Mercurio R, Eliott JA. Trick or treat? Australian newspaper portrayal of complementary and alternative medicine for the treatment of cancer. Support Care Cancer. 2011; 19: 67-80.

[320] Mocarski R, Bissell K. Edutainment's impact on health promotion: Viewing the biggest loser through the social cognitive theory. Health Promot Pract. 2016; 17: 107-115.

[321] Moodie C, Angus K, Ford A. The importance of cigarette packaging in a 'dark' market: the 'Silk Cut' experience. Tob Control. 2014; 23: 274-278.

[322] Moore ES, Rideout VJ. The online marketing of food to children: Is it just fun and games? J Public Policy Mark. 2007; 26: 202-220.

[323] Moreno MA, Briner LR, Williams A, Brockman L, Walker L, Christakis DA. A content analysis of displayed alcohol references on a social networking web site. J Adolescent Health. 2010; 47: 168-175.

[324] Moreno MA, Christakis DA, Egan KG, Brockman LN, Becker T. Associations between displayed alcohol references on Facebook and problem drinking among college students. Arch Pediat Adol Med. 2012; 166: 157-163.

[325] Moreno MA, Kacvinsky L, Pumper M, Wachowski L, Whitehill JM. Associations between social media displays and event-specific alcohol consumption by college students. WMJ. 2013; 112: 251-256.

[326] Morgenstern M, Schoeppe F, Campbell J, Braam MWG, Stoolmiller M, Sargent JD. Content themes of alcohol advertising in U.S. television: latent class analysis. Alcohol Clin Exp Res. 2015; 39: 1766-1774.

[327] Moriarty CM, Jensen JD, Stryker JE. Frequently cited sources in cancer news coverage: a content analysis examining the relationship between cancer news content and source citation. Cancer Causes Control. 2010; 21: 41-49.

[328] Morrison SD, Sutton SF, Mebane FE. Public health news frames in North Carolina newspaper coverage of the 100% Tobacco-Free Schools campaign? Sometimes. N C Med J. 2006; 67: 255-259.

[329] Moshrefzadeh A, Rice W, Pederson A, Okoli CTC. A content analysis of media coverage of the introduction of a smoke-free bylaw in Vancouver parks and beaches. Int J Environ Res Public Health. 2013; 10: 4444-4453.

[330] Movahhed T, Seifi S, Rashed Mohassel A, Dorri M, Khorakian F, Mohammadzadeh Z. Content analysis of Islamic Republic of Iran television food advertising related to oral health: appeals and performance methods. J Res Health Sci 2014; 14: 205-209.

[331] Myhre SL, Saphir MN, Flora JA, Howard KA, Gonzalez EM. Alcohol coverage in California newspapers: frequency, prominence, and framing. J Public Health Policy. 2002; 23: 172-190.

[332] Myrick JG, Holton AE, Himelboim I, Love B. #Stupidcancer: exploring a typology of social support and the role of emotional expression in a social media community. Health Commun. 2016; 31: 596-605.

[333] Nagelhout GE, Putte Bvd, Vries Hd, Crone M, Fong GT, Willemsen MC. The influence of newspaper coverage and a media campaign on smokers' support for smoke-free bars and restaurants and on secondhand smoke harm awareness: findings from the International Tobacco Control (ITC) Netherlands Survey. Tob Control. 2012; 21: 24-29.

[334] Nagler RH, Bigman CA, Ramanadhan S, Ramamurthi D, Viswanath K. Prevalence and framing of health disparities in local print news: implications for multilevel interventions to address cancer inequalities. Cancer Epidemiol Biomarkers Prev. 2016; 25: 603-612.

[335] Nan X, Zhao X, Yang B, Iles I. Effectiveness of cigarette warning labels: Examining the impact of graphics, message framing, and temporal framing. Health Commun. 2015; 30: 81-89.

[336] Nelson DE, Pederson LL, Mowery P, Bailey S, Sevilimedu V, London J, Babb S, Pechacek T. Trends in US newspaper and television coverage of tobacco. Tob Control. 2015; 24: 94-99.

[337] Nicholls J. Everyday, everywhere: alcohol marketing and social media--current trends. Alcohol Alcoholism. 2012; 47: 486-493.

[338] Nicholson RA, Kreuter MW, Lapka C, Wellborn R, Clark EM, Sanders-Thompson V, Jacobsen HM, Casey C. Unintended effects of emphasizing disparities in cancer communication to African-Americans. Cancer Epidem Biomar. 2008; 17: 2946-2953.

[339] Niederdeppe J, Davis KC, Farrelly MC, Yarsevich J. Stylistic features, need for sensation, and confirmed recall of national smoking prevention advertisements. J Commun. 2007; 57: 272-292.

[340] Niederdeppe J, Farrelly MC, Wenter D. Media advocacy, tobacco control policy change and teen smoking in Florida. Tob Control. 2007; 16: 47-52.

[341] Niederdeppe J, Fowler EF, Goldstein K, Pribble J. Does local television news coverage cultivate fatalistic beliefs about cancer prevention? J Commun. 2010; 60: 230-253.

[342] Niederdeppe J, Gollust SE, Jarlenski MP, Nathanson AM, Barry CL. News coverage of sugar-sweetened beverage taxes: pro- and antitax arguments in public discourse. Am J Public Health. 2013; 103: e92-98.

[343] Niederdeppe J, Lee T, Robbins R, Kim HK, Kresovich A, Kirshenblat D, Standridge K, Clarke CE, Jensen J, Fowler EF. Content and effects of news stories about uncertain cancer causes and preventive behaviors. Health Commun. 2014; 29: 332-346.

[344] Niederkrotenthaler T, Voracek M, Herberth A, Till B, Strauss M, Etzersdorfer E, Eisenwort B, Sonneck G. Role of media reports in completed and prevented suicide: Werther v. Papageno effects. Br J Psychiatry. 2010; 197: 234-243.

[345] Nixon L, Mejia P, Cheyne A, Wilking C, Dorfman L, Daynard R. "We're part of the solution": evolution of the food and beverage industry's framing of obesity concerns between 2000 and 2012. Am J Public Health. 2015; 105: 2228-2236.

[346] No E, Kelly B, Devi A, Swinburn B, Vandevijvere S. Food references and marketing in popular magazines for children and adolescents in New Zealand: a content analysis. Appetite. 2014; 83: 75-81.

[347] O'Connor A, Casey P. What it says in the papers: an audit. Ir J Psychol Med. 2001; 18: 68-71.

[348] Okuhara T, Ishikawa H, Okada H, Kiuchi T. Identification of gain- and loss-framed cancer screening messages that appeared in municipal newsletters in Japan. BMC Research Notes. 2014; 7: 896.

[349] Ortiz SE, Zimmerman FJ, Adler GJ, Jr. Increasing public support for food-industry related, obesity prevention policies: the role of a taste-engineering frame and contextualized values. Soc Sci Med. 2016; 156: 142-153.

[350] Ortiz SE, Zimmerman FJ, Gilliam FD. Weighing in: the taste-engineering frame in obesity expert discourse. Am J Public Health. 2015; 105: 554-559.

[351] Outley CW, Taddese A. A content analysis of health and physical activity messages marketed to African American children during after-school television programming. Arch Pediat Adol Med. 2006; 160: 432-435.

[352] Paek H-J, Hove T, Jeon J. Social media for message testing: a multilevel approach to linking favorable viewer responses with message, producer, and viewer influence on YouTube. Health Commun. 2013; 28: 226-236.

[353] Paek H-J, Kim S, Hove T, Huh JY. Reduced harm or another gateway to smoking? Source, message, and information characteristics of E-cigarette videos on YouTube. J Health Commun. 2014; 19: 545-560.

[354] Paek H-J, Reid LN, Choi H, Jeong HJ. Promoting health (implicitly)? A longitudinal content analysis of implicit health information in cigarette advertising, 1954-2003. J Health Commun. 2010; 15: 769-787.

[355] Paek H-J, Reid LN, Jeong HJ, Choi H, Krugman D. Five decades of promotion techniques in cigarette advertising: a longitudinal content analysis. Health Marketing Quarterly. 2012; 29: 1-17.

[356] Paek HJ, Kim KS, Hove T. Content analysis of antismoking videos on YouTube: message sensation value, message appeals, and their relationships with viewer responses. Health Educ Res. 2010; 25: 1085-1099.

[357] Page RM, Brewster A. Frequency of promotional strategies and attention elements in children's food commercials during children's programming blocks on US broadcast networks. Young Consumers. 2007; 8: 184-196.

[358] Park H, Reber BH. Using public relations to promote health: a framing analysis of public relations strategies among health associations. J Health Commun. 2010; 15: 39-54.

[359] Park H, Reber BH, Chon M. Tweeting as health communication: health organizations' use of Twitter for health promotion and public engagement. J Health Commun. 2016; 21: 188-198.

[360] Park S-Y, Morton CR. The role of regulatory focus, social distance, and involvement in anti-high-risk drinking advertising: A construal-level theory perspective. J Advertising. 2015; 44: 338-348.

[361] Parke H, Ashcroft R, Brown R, Marteau TM, Seale C. Financial incentives to encourage healthy behaviour: an analysis of U.K. media coverage. Health Expect. 2013; 16: 292-304.

[362] Patterson C, Katikireddi SV, Wood K, Hilton S. Representations of minimum unit pricing for alcohol in UK newspapers: a case study of a public health policy debate. J Public Health. 2015; 37: 40-49.

[363] Patterson C, Semple S, Wood K, Duffy S, Hilton S. A quantitative content analysis of UK newsprint coverage of proposed legislation to prohibit smoking in private vehicles carrying children. BMC Public Health. 2015; 15: 760.

[364] Pendleton LL, Smith C, Roberts JL. Drinking on television: a content analysis of recent alcohol portrayal. Brit J Addict. 1991; 86: 769-774.

[365] Peng W, Tang L. Health content in Chinese newspapers. J Health Commun. 2010; 15: 695-711.

[366] Phoutthakeo P, Otsuka K, Ito C, Sayamoungkhoun P, Kounnavong S, Jimba M. Cross-border promotion of formula milk in Lao People's Democratic Republic. J Paediatr Child. 2014; 50: 51-56.

[367] Pinsky I, Silva MTA. A frequency and content analysis of alcohol advertising on Brazilian television. J Stud Alcohol. 1999; 60: 394-399.

[368] Poston WSC, Haddock CK, Jahnke SA, Hyder ML, Jitnarin N. A content analysis of military commander messages about tobacco and other health issues in military installation newspapers: What do military commanders say about tobacco? Mil Med. 2015; 180: 708-717.

[369] Potter B, Sheeshka J, Valaitis R. Content analysis of infant feeding messages in a Canadian women's magazine, 1945 to 1995. J Nutr Educ. 2000; 32: 196-203.

[370] Potvin Kent M, Dubois L, Wanless A. Self-regulation by industry of food marketing is having little impact during children's preferred television. Int J Pediatr Obes. 2011; 6: 401-408.

[371] Potvin Kent M, Dubois L, Wanless A. A nutritional comparison of foods and beverages marketed to children in two advertising policy environments. Obesity. 2012; 20: 1829-1837.

[372] Potvin Kent M, Martin CL, Kent EA. Changes in the volume, power and nutritional quality of foods marketed to children on television in Canada. Obesity. 2014; 22: 2053-2060.

[373] Pounders KR, Lee S, Mackert M. Matching temporal frame, self-view, and message frame valence: Improving persuasiveness in health communications. J Advertising. 2015; 44: 388-402.

[374] Pribble JM, Goldstein KM, Fowler EF, Greenberg MJ, Noel SK, Howell JD. Medical news for the public to use? What's on local TV news. Am J Manag Care. 2006; 12: 170-176.

[375] Pribble JM, Goldstein KM, Majersik JJ, Barsan WG, Brown DL, Morgenstern LB. Stroke information reported on local television news: a national perspective. Stroke. 2006; 37: 1556-1557.

[376] Price A, Grann VR. Portrayal of complementary and alternative medicine for cancer by top online news sites. J Altern Complem Med. 2012; 18: 487-493.

[377] Primack BA, Nuzzo E, Rice KR, Sargent JD. Alcohol brand appearances in US popular music. Addiction. 2012; 107: 557-566.

[378] Puhl R, Peterson JL, Luedicke J. Fighting obesity or obese persons? Public perceptions of obesity-related health messages. Int J Obes. 2013; 37: 774-782.

[379] Puhl RM, Peterson JL, DePierre JA, Luedicke J. Headless, hungry, and unhealthy: a video content analysis of obese persons portrayed in online news. J Health Commun. 2013; 18: 686-702.

[380] Pujazon-Zazik MA, Manasse SM, Orrell-Valente JK. Adolescents' self-presentation on a teen dating web site: a risk-content analysis. J Adolescent Health. 2012; 50: 517-520.

[381] Rachul C, Caulfield T. The media and access issues: content analysis of Canadian newspaper coverage of health policy decisions. Orphanet J Rare Dis. 2015; 10: 102.

[382] Ramanadhan S, Mendez SR, Rao M, Viswanath K. Social media use by community-based organizations conducting health promotion: a content analysis. BMC Public Health. 2013; 13: 1129.

[383] Ramos C, Navas J. Influence of Spanish TV commercials on child obesity. Public Health. 2015; 129: 725-731.

[384] Rayens MK, Butler KM, Wiggins AT, Kostygina G, Langley RE, Hahn EJ. Recall and effectiveness of messages promoting smoke-free policies in rural communities. Nicotine Tob Res. 2016; 18: 1340-1347.

[385] Reinau D, Meier CR, Blumenthal R, Surber C. Skin cancer prevention, tanning and vitamin D: a content analysis of print media in Germany and Switzerland. Dermatol. 2016; 232: 2-10.

[386] Rhoades E, Jernigan DH. Risky messages in alcohol advertising, 2003-2007: results from content analysis. J Adolescent Health. 2013; 52: 116-121.

[387] Rhodes N, Roskos-Ewoldsen D, Eno CA, Monahan JL. The content of cigarette counter-advertising: are perceived functions of smoking addressed? J Health Commun. 2009; 14: 658-673.

[388] Ribisl KM, Lee RE, Henriksen L, Haladjian HH. A content analysis of Web sites promoting smoking culture and lifestyle. Health Educ Behav. 2003; 30: 64-78.

[389] Riker CA, Butler KM, Ricks JM, Record RA, Begley K, Anderson DG, Hahn EJ. Creating effective media messaging for rural smoke-free policy. Public Health Nurs. 2015; 32: 613-624.

[390] Riles JM, Sangalang A, Hurley RJ, Tewksbury D. Framing cancer for online news: Implications for popular perceptions of cancer. J Commun. 2015; 65: 1018-1040.

[391] Rissel C, Bonfiglioli C, Emilsen A, Smith BJ. Representations of cycling in metropolitan newspapers - changes over time and differences between Sydney and Melbourne, Australia. BMC Public Health. 2010; 10.

[392] Roberts M, Pettigrew S, Chapman K, Quester P, Miller C. The advertised diet: an examination of the extent and nature of food advertising on Australian television. Health Promot J Aust. 2013; 24: 137-142.

[393] Robinson T, Callister M, Jankoski T. Portrayal of body weight on children's television sitcoms: a content analysis. Body Image. 2008; 5: 141-151.

[394] Rose ID, Friedman DB, Marquez DX, Fernandez K. What are older Latinos told about physical activity and cognition? A content analysis of a top-circulating magazine. J Aging Health. 2013; 25: 1143-1158.

[395] Roseman MG, Poor M, Stephenson TJ. A content analysis of food references in television programming specifically targeting viewing audiences aged 11 to 14 years. J Nutr Educ Behav. 2014; 46: 20-25.

[396] Saguy AC, Gruys K, Gong S. Social problem construction and national context: news reporting on "overweight" and "obesity" in the United States and France. Soc Probl. 2010; 57: 586-610.

[397] Schneider EP, McGovern EE, Lynch CL, Brown LS. Do food blogs serve as a source of nutritionally balanced recipes? An analysis of 6 popular food blogs. J Nutr Educ Behav. 2013; 45: 696-700.

[398] Schulz PJ, Hartung U. What to eat in the land of cheese and chocolate: a content analysis of Swiss print media messages on a healthy diet. Communication & Medicine. 2011; 8: 99-110.

[399] Scully M, Makin J, Maloney S, Wakefield M. Changes in coverage of sun protection in the news: threats and opportunities from emerging issues. Health Educ Res. 2014; 29: 378-387.

[400] Scully M, Wakefield M, Dixon H. Trends in news coverage about skin cancer prevention, 1993-2006: increasingly mixed messages for the public. Aust NZ J Publ Heal. 2008; 32: 461-466.

[401] Scully P, Macken A, Leddin D, Cullen W, Dunne C, Gorman CO. Food and beverage advertising during children's television programming. Irish J Med Sci. 2015; 184: 207-212.

[402] Segar ML, Updegraff JA, Zikmund-Fisher BJ, Richardson CR. Physical activity advertisements That feature daily well-being improve autonomy and body image in overweight women but not men. J Obes. 2012; 2012: Article-354721.

[403] Seidenberg AB, Rodgers EJ, Rees VW, Connolly GN. Youth access, creation, and content of smokeless tobacco ("dip") videos in social media. J Adolescent Health. 2012; 50: 334-338.

[404] Sepe E, Glantz SA. Bar and club tobacco promotions in the alternative press: targeting young adults. Am J Public Health. 2002; 92: 75-78.

[405] Shea R, Chapman S. Media reports of rural health and safety: a review of articles published in The Land newspaper. Aust J Rural Health. 2001; 9: 206-208.

[406] Shim M, Kim Y-C, Kye SY, Park K. News portrayal of cancer: content analysis of threat and efficacy by cancer type and comparison with incidence and mortality in Korea. J Korean Med Sci. 2016; 31: 1231-1238.

[407] Shugart H. Heavy viewing: Emergent frames in contemporary news coverage of obesity. Health Commun. 2011; 26: 635-648.

[408] Shugart HA. Weight of tradition: culture as a rationale for obesity in contemporary U.S. news coverage. Obes Rev. 2013; 14: 736-744.

[409] Silver Wallace L, Leenders N. Content analysis of prime-time television coverage of physical activity, 1970-2001. Am J Prev Med. 2004; 26: 130-134.

[410] Simunaniemi A-M, Sandberg H, Andersson A, Nydahl M. Laypeople blog about fruit and vegetables for self-expression and dietary influence. Health Commun. 2011; 26: 621-630.

[411] Siu W. Social construction of reality: the tobacco issue. Crit Public Health. 2009; 19: 23-44.

[412] Sixsmith R, Furnham A. A content analysis of British food advertisements aimed at children and adults. Health Promot Int. 2010; 25: 24-32.

[413] Slater MD, Long M, Bettinghaus EP, Reineke JB. News coverage of cancer in the United States: a national sample of newspapers, television, and magazines. J Health Commun. 2008; 13: 523-537.

[414] Sloane K, Wilson N, Imlach Gunasekara F. A content analysis of the portrayal of alcohol in televised music videos in New Zealand: changes over time. Drug Alcohol Rev. 2013; 32: 47-52.

[415] Smith BJ, Bonfiglioli CMF. Physical activity in the mass media: an audience perspective. Health Educ Res. 2015; 30: 359-369.

[416] Smith E. 'It's interesting how few people die from smoking': tobacco industry efforts to minimize risk and discredit health promotion. Eur J Public Health. 2007; 17: 162-170.

[417] Smith E. Corporate image and public health: an analysis of the Philip Morris, Kraft, and Nestle websites. J Health Commun. 2012; 17: 582-600.

[418] Smith E, Offen N, Malone RE. What makes an ad a cigarette ad? Commercial tobacco imagery in the lesbian, gay, and bisexual press. Journal of Epidemiology and Community Health. 2005; 59: 1086-1091.

[419] Smith KC, Cukier S, Jernigan DH. Regulating alcohol advertising: content analysis of the adequacy of federal and self-regulation of magazine advertisements, 2008-2010. Am J Public Health. 2014; 104: 1901-1911.

[420] Smith KC, McLeod K, Wakefield M. Australian letters to the editor on tobacco: triggers, rhetoric, and claims of legitimate voice. Qual Health Res. 2005; 15: 1180-1198.

[421] Smith KC, Wakefield M. Textual analysis of tobacco editorials: how are Key media gatekeepers framing the issues? Am J Health Promot. 2005; 19: 361-368.

[422] Smith KC, Wakefield M, Terry-McElrath Y, Chaloupka FJ, Flay B, Johnston L, Saba A, Siebel C. Relation between newspaper coverage of tobacco issues and smoking attitudes and behaviour among American teens. Tob Control. 2008; 17: 17-24.

[423] Snider S. Revisioning fat lesbian subjects in contemporary lesbian periodicals. J Lesbian Stud. 2010; 14: 174-184.

[424] Soo J, Letona P, Chacon V, Barnoya J, Roberto CA. Nutritional quality and child-oriented marketing of breakfast cereals in Guatemala. Int J Obes. 2016; 40: 39-44.

[425] Spencer RJ, Russell JM, Barker ME. Temporality in British young women's magazines: food, cooking and weight loss. Public Health Nutr. 2014; 17: 2359-2367.

[426] Squiers LB, Holden DJ, Dolina SE, Kim AE, Bann CM, Renaud JM. The public's response to the U.S. Preventive Services Task Force's 2009 Recommendations on Mammography Screening. Am J Prev Med. 2011; 40: 497-504.

[427] Stefanik-Sidener K. Nature, nurture, or that fast food hamburger: media framing of diabetes in the New York Times from 2000 to 2010. Health Commun. 2013; 28: 351-358.

[428] Stellefson M, Chaney B, Ochipa K, Chaney D, Haider Z, Hanik B, Chavarria E, Bernhardt JM. YouTube as a source of chronic obstructive pulmonary disease patient education: a social media content analysis. Chron Resp Dis. 2014; 11: 61-71.

[429] Sterling KL, Fryer CS, Majeed B, Duong MM. Promotion of waterpipe tobacco use, its variants and accessories in young adult newspapers: a content analysis of message portrayal. Health Educ Res. 2015; 30: 152-161.

[430] Stitt C, Kunkel D. Food advertising during children's television programming on broadcast and cable channels. Health Commun. 2008; 23: 573-584.

[431] Story M, Faulkner P. The prime time diet: a content analysis of eating behavior and food messages in television program content and commercials. Am J Public Health. 1990; 80: 738-740.

[432] Street AF. Ask your doctor: the construction of smoking in advertising posters produced in 1946 and 2004. Nurs Inq. 2004; 11: 226-237.

[433] Stryker JE, Fishman J, Emmons KM, Viswanath K. Cancer risk communication in mainstream and ethnic newspapers. Prev Chronic Dis. 2009; 6: A23.

[434] Stryker JE, Moriarty CM, Jensen JD. Effects of newspaper coverage on public knowledge about modifiable cancer risks. Health Commun. 2008; 23: 380-390.

[435] Stryker JE, Solky BA, Emmons KM. A content analysis of news coverage of skin cancer prevention and detection, 1979 to 2003. Arch Dermatol. 2005; 141: 491-496.

[436] Sukumaran A, Diwakar MP, Shastry SM. A content analysis of advertisements related to oral health in children's Tamil television channels--a preliminary report. Int J Paediatr Dent. 2012; 22: 232-238.

[437] Sun Y, Krakow M, John KK, Liu M, Weaver J. Framing obesity: how news frames shape attributions and behavioral responses. J Health Commun. 2016; 21: 139-147.

[438] Syed-Abdul S, Fernandez-Luque L, Jian W-S, Li Y-C, Crain S, Hsu M-H, Wang Y-C, Khandregzen D, Chuluunbaatar E, Nguyen PA *et al*. Misleading health-related information promoted through video-based social media: anorexia on YouTube. J Med Internet Res. 2013; 15: 137-149.

[439] Szklo AS, Coutinho ESF. The influence of smokers' degree of dependence on the effectiveness of message framing for capturing smokers for a Quitline. Addict Behav. 2010; 35: 620-624.

[440] Team V, Markovic M. Internet advertising of artificial tanning in Australia. Eur J Cancer Prev. 2006; 15: 371-376.

[441] Thackeray R, Burton SH, Giraud-Carrier C, Rollins S, Draper CR. Using twitter for breast cancer prevention: an analysis of breast cancer awareness month. BMC Cancer. 2013; 13.

[442] Theberge N. A content analysis of print media coverage of gender, women, and physical activity. J Appl Sport Psychol. 1991; 3: 36-48.

[443] Thomas SL, Olds T, Pettigrew S, Yeatman H, Hyde J, Dragovic C. Parent and child interactions with two contrasting anti-obesity advertising campaigns: a qualitative analysis. BMC Public Health. 2014; 14: 151.

[444] Thompson DA, Flores G, Ebel BE, Christakis DA. Comida en venta: After-school advertising on Spanish-language television in the United States. J Pediatr. 2008; 152: 576-581.

[445] Thompson TL, Robinson JD, Cusella LP, Shellabarger S. Women's health problems in soap operas: a content analysis. Womens Health Issues. 2000; 10: 202-209.

[446] Thornley L, Signal L, Thomson G. Does industry regulation of food advertising protect child rights? Crit Public Health. 2010; 20: 25-33.

[447] Thrasher JF, Kim SH, Rose I, Navarro A, Craft MK, Davis KJ, Biggers S. Print media coverage around failed and successful tobacco tax initiatives: the South Carolina experience. Am J Health Promot. 2014; 29: 29-36.

[448] Till B, Niederkrotenthaler T. Surfing for suicide methods and help: content analysis of websites retrieved with search engines in Austria and the United States. J Clin Psychiatry. 2014; 75: 886-892.

[449] Timberlake DS, Pechmann C, Tran SY, Au V. A content analysis of Camel Snus advertisements in print media. Nicotine Tob Res. 2011; 13: 431-439.

[450] Toll BA, Salovey P, O'Malley SS, Mazure CM, Latimer A, McKee SA. Message framing for smoking cessation: the interaction of risk perceptions and gender. Nicotine Tob Res. 2008; 10: 195-200.

[451] Tong A, Chapman S, Sainsbury P, Craig JC. An analysis of media coverage on the prevention and early detection of CKD in Australia. Am J Kidney Dis. 2008; 52: 159-170.

[452] Toohey AM, Rock MJ. Newspaper portrayals, local policies, and dog-supportive public space: who's wagging whom? Anthrozoos. 2015; 28: 549-567.

[453] Tyrrell I. The limits of persuasion: advertising, gender and the culture of Australian smoking. Aust Historical Stud. 2000; 31: 27-48.

[454] Ulijaszek SJ, McLennan AK. Framing obesity in UK policy from the Blair years, 1997-2015: the persistence of individualistic approaches despite overwhelming evidence of societal and economic factors, and the need for collective responsibility. Obes Rev. 2016; 17: 397-411.

[455] Van Den Bulck H, Simons N, Gorp BV. Let's drink and be merry: the framing of alcohol in the prime-time American youth series The OC. J Stud Alcohol Drugs. 2008; 69: 933-940.

[456] van der Wardt EM, Taal E, Rasker JJ, Wiegman O. Media coverage of chronic diseases in the Netherlands. Semin Arthritis Rheum. 1999; 28: 333-341.

[457] van Hoof J, van Noordenburg M, de Jong M. Happy hours and other alcohol discounts in cafes: prevalence and effects on underage adolescents. J Public Health Policy. 2008; 29: 340-352.

[458] van Hoof JJ, de Jong MDT, Fennis BM, Gosselt JF. There's alcohol in my soap: portrayal and effects of alcohol use in a popular television series. Health Educ Res. 2009; 24: 421-429.

[459] van Kleef E, van Trijp HCM, Luning P. Functional foods: health claim-food product compatibility and the impact of health claim framing on consumer evaluation. Appetite. 2005; 44: 299-308.

[460] Wackowski OA, Lewis MJ, Delnevo CD, Ling PM. A content analysis of smokeless tobacco coverage in U.S. newspapers and news wires. Nicotine Tob Res. 2013; 15: 1289-1296.

[461] Wackowski OA, Lewis MJ, Hrywna M. Banning smoking in New Jersey casinos - a content analysis of the debate in print media. Subst Use Misuse. 2011; 46: 882-888.

[462] Wakefield M, McLeod K, Smith KC. Individual versus corporate responsibility for smoking-related illness: Australian press coverage of the Rolah McCabe trial. Health Promot Int. 2003; 18: 297-305.

[463] Wakefield M, Smith KC, Chapman S. Framing of Australian newspaper coverage of a secondhand smoke injury claim: lessons for media advocacy. Crit Public Health. 2005; 15: 53-63.

[464] Wakefield MA, Brennan E, Durkin SJ, McLeod K, Smith KC. Still a burning issue: trends in the volume, content and population reach of newspaper coverage about tobacco issues. Crit Public Health. 2011; 21: 313-325.

[465] Wakefield MA, Brennan E, Durkin SJ, McLeod K, Smith KC. Making news: the appearance of tobacco control organizations in newspaper coverage of tobacco control issues. Am J Health Promot. 2012; 26: 166-171.

[466] Wallack L, Dorfman L. Health messages on television commercials. Am J Health Promot. 1992; 6: 190-196.

[467] Wallington SF, Blake KD, Taylor-Clark K, Viswanath K. Antecedents to agenda setting and framing in health news: an examination of priority, angle, source, and resource usage from a national survey of U.S. health reporters and editors. J Health Commun. 2010; 15: 76-94.

[468] Wallington SF, Blake KD, Taylor-Clark K, Viswanath K. Challenges in covering health disparities in local news media: an exploratory analysis assessing views of journalists. J Commun Health. 2010; 35: 487-494.

[469] Walter T. Jade and the journalists: Media coverage of a young British celebrity dying of cancer. Soc Sci Med. 2010; 71: 853-860.

[470] Warner KE. Tobacco industry response to public health concern: a content analysis of cigarette ads. Health Educ Quart. 1985; 12: 115-127.

[471] Warsh CK, Tinkler P. In Vogue: North American and British representations of women smokers in Vogue, 1920s-1960s. Canadian Bulletin of Medical History. 2007; 24: 9-47.

[472] Webb MS, Baker EA, Rodriguez de Ybarra D. Effects of culturally specific cessation messages on theoretical antecedents of behavior among low-income african american smokers. Psychol Addict Behav. 2010; 24: 333-341.

[473] Weeks BE, Friedenberg LM, Southwell BG, Slater JS. Behavioral consequences of conflict-oriented health news coverage: the 2009 mammography guideline controversy and online information seeking. Health Commun. 2012; 27: 158-166.

[474] Weeks L, Verhoef M, Scott C. Presenting the alternative: cancer and complementary and alternative medicine in the Canadian print media. Support Care Cancer. 2007; 15: 931-938.

[475] Wellard L, Hughes C, Tsang YW, Watson W, Chapman K. Investigating fruit and vegetable claims on Australian food packages. Public Health Nutr. 2015; 18: 2729-2735.

[476] Wenger L, Malone R, Bero L. The cigar revival and the popular press: a content analysis, 1987-1997. Am J Public Health. 2001; 91: 288-291.

[477] Wilkin HA, Gonzalez C, Tannebaum M. Evaluating health storytelling in Spanish-language television from a communication infrastructure theory perspective. Howard Journal of Communications. 2015; 26: 403-421.

[478] Williams RS, Schmidt A. The sales and marketing practices of English-language internet alcohol vendors. Addiction. 2014; 109: 432-439.

[479] Willis LE, Knobloch-Westerwick S. Weighing women down: messages on weight loss and body shaping in editorial content in popular women's health and fitness magazines. Health Commun. 2014; 29: 323-331.

[480] Wilson N, Signal L, Nicholls S, Thomson G. Marketing fat and sugar to children on New Zealand television. Prev Med. 2006; 42: 96-101.

[481] Wilson N, Sloane K, Gunasekara FI, Thomson G. Portrayal of tobacco in televised music videos: content analysis and trends. N Z Med J. 2011; 124: 90-93.

[482] Wise D, Brewer PR. Competing frames for a public health issue and their effects on public opinion. Mass Communication & Society. 2010; 13: 435-457.

[483] Wong CO, McMurray NE. Framing communication: communicating the antismoking message effectively to all smokers. J Community Psychol. 2002; 30: 433-448.

[484] Wood K, Patterson C, Katikireddi SV, Hilton S. Harms to 'others' from alcohol consumption in the minimum unit pricing policy debate: a qualitative content analysis of U.K. newspapers (2005-12). Addiction. 2014; 109: 578-584.

[485] Wyllie J, Baxter S, Kulczynski A. Healthy kids: Examining the effect of message framing and polarity on children's attitudes and behavioral intentions. J Advertising. 2015; 44: 140-150.

[486] Yanovitzky I, Blitz CL. Effect of media coverage and physician advice on utilization of breast cancer screening by women 40 years and older. J Health Commun. 2000; 5: 117-134.

[487] Yao T, Jiang N, Grana R, Ling PM, Glantz SA. A content analysis of electronic cigarette manufacturer websites in China. Tob Control. 2016; 25: 188-194.

[488] Ye Y, Ward KE. The depiction of illness and related matters in two top-ranked primetime network medical dramas in the United States: a content analysis. J Health Commun. 2010; 15: 555-570.

[489] Yeh MA, Jewell RD. The myth/fact message frame and persuasion in advertising: enhancing attitudes toward the mentally ill. J Advertising. 2015; 44: 161-172.

[490] Yi Z, Xu R, Zhao K, Li K. Television news coverage of obesity in China, 1982-2009. Biomed Environ Sci. 2012; 25: 325-333.

[491] Yoo JH, Kim J. Obesity in the new media: a content analysis of obesity videos on YouTube. Health Commun. 2012; 27: 86-97.

[492] Yoon S, Lam T. The illusion of righteousness: corporate social responsibility practices of the alcohol industry. BMC Public Health. 2013; 13.

[493] Zhao G, Pechmann C. The impact of regulatory focus on adolescents' response to antismoking advertising campaigns. J Mark Res. 2007; 44: 671-687.

[494] Zharekhina L, Kubacki K. What messages does social marketing advertising send? A content analysis of advertisements aiming to minimise harm from alcohol consumption. Int J Nonprofit Volunt Sect Mark. 2015; 20: 285-298.

[495] Zwarun L. Ten years and 1 master settlement agreement later: the nature and frequency of alcohol and tobacco promotion in televised sports, 2000 through 2002. Am J Public Health. 2006; 96: 1492-1497.

[496] Zwier S. Medicalisation of food advertising. Nutrition and health claims in magazine food advertisements 1990-2008. Appetite. 2009; 53: 109-113.
